# A Solvent‐Free Approach for the Mechanochemical Upcycling of Bio‐ and Fossil‐Based Polyesters Into Value‐Added Chemicals and Bio‐Based Plasticizers

**DOI:** 10.1002/cssc.70975

**Published:** 2026-08-31

**Authors:** Divya Jain, Nora Jannsen, Riko Siewert, Dzmitry Zaitsau, Hans‐Joachim Drexler, Torsten Beweries

**Affiliations:** ^1^ Leibniz Institute for Catalysis (LIKAT Rostock) Rostock Germany; ^2^ Lehrstuhl für Technische Thermodynamik Universität Rostock Rostock Germany; ^3^ Department Life, Light & Matter, Faculty of Interdisciplinary Research University of Rostock Rostock Germany

**Keywords:** amidation, furan, polyester, transesterification, upcycling

## Abstract

Plastic waste poses a major environmental challenge, yet it also represents a valuable feedstock to produce high‐value chemicals. In this work, we report an ecofriendly and efficient mechanochemical strategy for upcycling of bio‐ and fossil‐based polyesters under mild conditions into synthetically useful building blocks. Bio‐based polyethylene furanoate (PEF), polybutylene furanoate (PBF), and polylactic acid (PLA), as well as fossil‐derived polyethylene terephthalate (PET), were successfully transformed into their corresponding transesterification and amidation products in excellent yields using sodium methoxide as a catalyst. These reactions generate the corresponding diol, methanol, and sodium chloride as byproducts, which can be recovered and reused. Furthermore, using the same protocol, PEF was converted into bio‐based plasticizers, including diethylhexyl furanate (DEHF) and diisoamyl furanoate (DIAF) in excellent yields. Importantly, the method is not limited to pure polymers but is also effective for commercially available PET‐ and PLA‐based packaging materials. The products were isolated by simple aqueous workup and characterized using NMR, IR, HRMS, and XRD techniques. Overall, this mechanochemical route offers a sustainable, cost‐effective, and versatile approach for polyester waste valorization, contributing significantly to a circular plastic economy.

## Graphical abstract

Mechanochemical upcycling of bio‐ and fossil‐based polyesters with alcohols and amines using MeONa as the catalyst selectively produces synthetically useful diesters and diamides, giving only alcohols as byproducts. This green and efficient protocol is readily scalable and can be transferred to the upcycling of polyester‐based postconsumer waste materials.

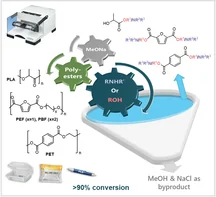

## 1 Introduction

Plastics are widely recognized as highly versatile materials due to their lightweight, durability, flexibility, and low production cost. Global plastic production has experienced an extraordinary surge over the past decades. In 1950, annual output was approximately two million tons. In 2019, it exceeds 450 million tons, reflecting a more than 200‐fold increase [1]. According to a recent survey, an estimated 170 trillion plastic particles are afloat in the world’s oceans, endangering human health, marine life, and entire ecosystems [2]. Plastics are generally classified into fossil‐based plastics, derived from petrochemical sources, and bio‐based plastics, produced from renewable biomass such as starch, cellulose, or vegetable oils. Both types can be engineered to be biodegradable or nonbiodegradable, depending on their chemical structure [3]. Common plastic categories include polyolefins (e.g., polyethylene, polypropylene, polystyrene, and polyvinyl chloride) and condensation polymers (e.g., polyethylene terephthalate [PET], polycarbonates, and polyamides). Despite their undeniable utility, plastics pose serious environmental and health risks.

Polyesters are among the most widely used synthetic and bio‐based polymers owing to their favorable thermal, mechanical, and barrier properties and being used in industrial textiles, medical textiles, home textiles, clothing, etc [4, 5]. Polyethylene furanoate (PEF) as an example for a bio‐based polyester has emerged as a promising ecofriendly alternative to widely used PET (global annual production of 55 million tons in 2021 [6]) with significant potential to reduce greenhouse gas emissions. PEF is of renewable origin and possesses superior barrier properties [7, 8, 9]. The enormous potential of PEF is reflected in recent estimations of its market value (21.3 million USD in 2024), market demand (300 t in 2025), and annual growth rates (82%, corresponding to a market demand of 122.000 t in 2034) [6]. Polylactic acid (PLA) is a biodegradable, bio‐based thermoplastic polyester derived from renewable resources like corn starch or sugarcane that is mainly used for short‐lived and disposable packaging (global annual production of approximately 0.91 million tons in 2024 [10]). Ester linkages of polyesters render them suitable for diverse chemical recycling and upcycling strategies, including hydrolysis, methanolysis, glycolysis, aminolysis, and transesterification, as well as enzymatic and catalytic depolymerization routes. While these methods can effectively recover monomers or generate value‐added products [11, 12, 13, 14, 15, 16, 17, 18, 19], they often require elevated temperatures, pressures, or excess solvents, limiting their environmental and economic sustainability. Recently, mechanochemical approaches have gained attention as a greener alternative, offering solvent‐free or solvent‐minimal conditions for depolymerization and functionalization of synthetic polymers such as polyesters [20, 21, 22]. Mechanochemistry has been shown to be a highly attractive technique for degrading even highly insoluble plastics. In earlier reports, mechanochemical ball milling combined with alkaline hydrolysis has emerged as an efficient approach for the depolymerization of polyesters into their monomer units, enabling rapid and near‐quantitative conversion of waste PET under solvent‐free conditions, with reaction rates governed by milling energy, and demonstrating applicability to bio‐based polyesters such as PEF and polybutylene furanoate (PBF) [23, 24, 25, 26].

**FIGURE 1:**
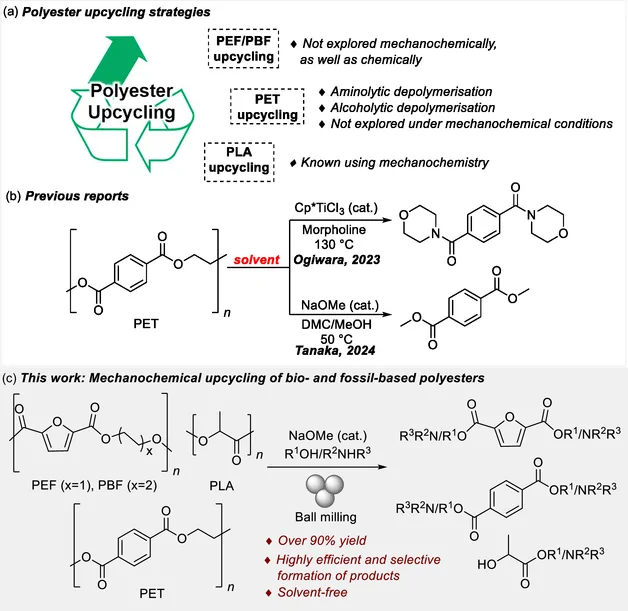
Contextualization of the present work.

Mechanochemical upcycling of polyesters, particularly PET, has predominantly been studied through solvolysis at high‐temperature and high‐pressure conditions (Figure 1) [27, 28, 29, 30, 31]. However, it has so far only rarely been explored mechanochemically. In a recent contribution, Kim, Borchardt and coworkers reported the mechanochemical recycling of a polycarbonate and polyesters (PET and PLA) using methanol [10]. More recently, resonant acoustic mixing (RAM) has been applied to the mechanochemical upcycling of PLA [32]. However, the mechanochemical valorization of bio‐based polyesters such as PEF and PBF remains underdeveloped [26].

Building on our previous work [26], we now demonstrate that sodium methoxide (MeONa) serves as a highly efficient catalyst for the amidation and transesterification of various polyesters, such as PEF, PBF, PET, and PLA. This represents the first report of the mechanochemical amidation and transesterification of bio‐based PEF and PBF. MeONa has previously been reported as a catalyst for the amidation of methyl esters using toluene as a solvent [33] and, more recently, Tanaka and coworkers employed it in PET methanolysis with dimethyl carbonate under solvolysis conditions [34]. In parallel, Aniya and coworkers reported the mechanochemical depolymerization of PET into a plasticizer using an organometallic catalyst [35]. Our protocol extends this reactivity beyond methanolysis, offering a general approach to amidation and transesterification of various polyesters using catalytic amounts of MeONa. These features impart environmental advantages, including high atom economy and reduced waste. Importantly, our method enables the synthesis of bio‐based plasticizers in excellent yield and purity through the transesterification of PEF, highlighting its potential for sustainable polymer degradation and upcycling.

## 2 Results and Discussion

Our studies of mechanochemical upcycling of polyesters started with PEF and PBF, synthesized by polycondensation of dimethyl furanoate and the corresponding diols and used for our previous depolymerization work [22]. Later, we have explored the applicability of our protocol for pure PET and PLA samples and extended our approach for packaging‐based PET (postPET) and PLA materials (postPLA, taken from a plastic pen). The studied polymers were analyzed using size‐exclusion chromatography (SEC), differential scanning calorimetry (DSC), and thermal gravimetric analysis (TGA) (Table 1). A detailed description of the SEC, TGA, and DSC studies can be found in Supporting Information.

**TABLE 1:** Properties of polyesters used in this study.

Polymers	Number average molecular weight *M*_n_, g/mol^a^	Dispersity *Ɖ*, *M*_w_/*M*_n_ a	Degradation temperature *T*_d_, °C^b^	Glass transition temperature *T*_g_, °C^c^	Melting temperature *T*_m_, °C^c^
PEF^d^	21,300	1.82	>250	85	218
PBF^d^	33,400	2.06	>250	46	171
PET	47,200	2.29	>250	80^e^	237
PLA	106,000	1.79	200	55^f^	157
Packaging PET (postPET)	37,400	2.23	>250	—	225, 236^g^
Packaging PLA (postPLA)	57,200	1.94	150	49^f^	120, 139^g^

a Number average molecular weights, obtained from SEC in pentafluorophenol/chloroform (v/v = 1:1).

b Obtained from TGA.

c Obtained from DSC analysis.

d Values from the literature, ref. [26].

e According to an experimental study by Androsch [36], cooling with a 10 K·min^−1^ rate in the DSC technique leads to a high degree of crystallinity of the PET polymer and glass transition could not be observed.

f At 5 K/min.

g Due to multicomponent composition, multiple peaks can be seen with the largest peak at 2365 °C (postPET) and 139 °C (postPLA).

Polymer recycling generally follows two main routes: mechanical reprocessing, which includes remelting and transformation into new consumer products (e.g., packaging or pen components), and chemical recycling via depolymerization to recover valuable chemical building blocks. The selection of the more suitable route strongly depends on the thermal stability and melting behavior of the respective materials, as these properties indicate whether the materials can be thermally reprocessed or not. The thermal properties of virgin PET granulate and postPET are overall very similar. The TGA curves show an almost identical temperature dependence of mass loss for both PET samples, indicating comparable thermal stability. In particular, both PET variants remain thermally stable up to at least 250 °C under the applied conditions. Regarding the melting temperatures, PET shows one melting peak at 237 °C, whereas postPET exhibits two melting peaks: one at 225 and 236 °C.

In contrast to PET, pronounced differences are observed between PLA powder and postPLA. For postPLA, two melting endotherms are detected at 120 and 139 °C. They occur 17–37 °C below the melting temperature 157 °C of the PLA powder. Furthermore, it was observed that the onset melting temperature decreased by approximately 2 °C for PLA powder and 3 °C for postPLA with each DSC run. At the same time, the relative peak areas of the two melting endotherms changed. This indicates a possible slow degradation of the PLA sample in the molten state (e.g., by chain scission). The decrease in glass transition temperature from virgin PLA (55 °C) to postPLA (49 °C) provides further evidence that PLA may undergo degradation during repeated melting. In addition, the degradation temperature from TGA decreases substantially from about 175 °C for the PLA powder to approximately 150 °C for postPLA. The temperature difference between the degradation temperature and the second melting temperature is 11 °C for post‐PLA (*T*_d_ = 150 °C vs. *T*_m,2_ = 139 °C), which is smaller than that for PLA powder. If this gap continues to decrease upon recycling, repeated melt reprocessing would entail an increasing risk of thermal degradation. Together, the reduced melting temperatures and the markedly lower thermal stability indicate significant structural changes in the PLA from the consumer article. Consequently, approaches such as mechanochemical upcycling appear as a promising alternative to remelting and reprocessing, as they can convert structurally compromized polymer waste into valuable building blocks.

### 2.1 Alcoholysis of Polyesters

We began our investigation of the mechanochemical upcycling of polyesters via alcoholysis using 4‐methoxybenzyl alcohol (**2a**) as an aliphatic alcohol component that contains the OMe group as an excellent NMR spectroscopic handle. This naturally occurring colorless liquid is widely used as a fragrance and flavoring agent. PEF (182 mg, 1 mmol based on the repeating unit, 1 equiv.) and **2a** (2 equiv.) were milled together in the presence of various bases for 60–90 min at 30 Hz in a 10 mL stainless steel (SS) milling jar containing two 10 mm SS milling balls. To evaluate the scope for further optimization, we first investigated the use of a stoichiometric amount of Na_2_CO_3_ (3 equiv.) as a base. No depolymerization of PEF was observed after 90 min of milling under these conditions (Table 2, entry 1). We next examined the *t*BuNH_2_/NaCl catalytic system reported in the literature [37] for polyester transesterification; however, no formation of the desired product was detected (Table 2, entry 2). Similarly, organocatalytic systems based on the 1,8‐diazabicyclo [5.4.0]undec‐7‐ene (DBU)/methylsulfonic acid (MSA) salt and pyridinium *p*‐toluenesulfonate (PPTS) failed to promote PEF depolymerization under mechanochemical conditions, and no transesterification product was observed (Table 2, entries 3 and 4). Subsequently, stronger bases such as NaOH and MeONa were evaluated. While NaOH did not afford the expected alcoholysis product (Table 2, entry 5), catalytic amounts of MeONa proved highly effective, facilitating the alcoholysis of PEF in excellent yields. Following these observations, the reaction conditions were further optimized with respect to catalyst loading and milling time (Table 2, entries 6–11). Complete conversion of PEF into the corresponding transesterification product was achieved using 30 mol% MeONa within 60 min. Under these optimized conditions, bis(4‐methoxybenzyl)‐furan‐2,5‐dicarboxylate (**3c**) was isolated in 98% yield after thorough washing of the reaction mixture with water. The workup procedure for the mechanochemical upcycling of polyesters was also optimized. Solid products were isolated by extensive washing with water, whereas liquid products were purified by acidic or basic extraction protocols using ethyl acetate or diethyl ether, depending on the nature of the product and the starting material. These reactions generate corresponding ethylene glycol, methanol, and sodium chloride as byproducts, which can be recovered and reused [26]. In summary, among all the catalytic systems investigated, only catalytic amounts of MeONa efficiently promoted the alcoholysis of PEF, providing the desired transesterification product in excellent yield under solvent‐free mechanochemical conditions. All products were characterized by NMR and IR spectroscopy, high‐resolution mass spectrometry (HRMS), and, where applicable, X‐ray crystallographic analysis (see Supporting Information for details).

**TABLE 2:** Summary of reaction conditions used during optimization of the mechanochemical alcoholysis of PEF.^a^

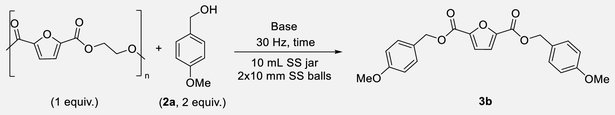				
Entry	Base/catalyst	Time, min	**PEF conversion into product, %** b	**Yield, %** c
1.	Na_2_CO_3_ (3 eq.)	90	0	0
2.	^*t*^BuNH_2_/NaCl (2–4 eq.)	90	0	0
3.	DBU:MSA salt (0.2–0.5 eq.)	60	0	0
4.	PPTS (0.5 eq.)	60	0	0
5.	NaOH (2.5 eq)	90	0	0
6.	MeONa (0.4 eq.)	60^d^	85	81
7.	MeONa (0.4 eq.)	60	99	>98
**8.**	**MeONa (0.3 eq.)**	**60**	** >98**	**98**
9.	MeONa (0.2 eq.)	90	>95	94
10.	None	90	0	0
11.	MeONa (0.3 eq.)	90^e^	>96	96

*Note:* Bold indicates the optimized reaction conditions.

Abbreviations: DBU, 1,8‐diazabicyclo [5.4.0]undec‐7‐ene; MSA, methylsulfonic acid; PPTS, pyridinium *p*‐toluensulfonate; SS, stainless steel.

a Reaction conditions: PEF (182 mg, 1 mmol per repeating unit, 1 equiv.) and 4‐methoxybenzyl alcohol (2 mmol, 2 equiv.) were ball milled in the presence of base/catalyst at 30 Hz.

b Conversion was calculated by measuring the remaining amount of polymer after mechanochemical upcycling reaction.

c Product isolated yields have been calculated upon measuring and calculating the mmols of obtained product and dividing it by mmols of polymer repeating unit used for the reaction.

d 25 Hz ball milling frequency.

e Use of ZrO_2_ jar and milling balls.

**FIGURE 2:**
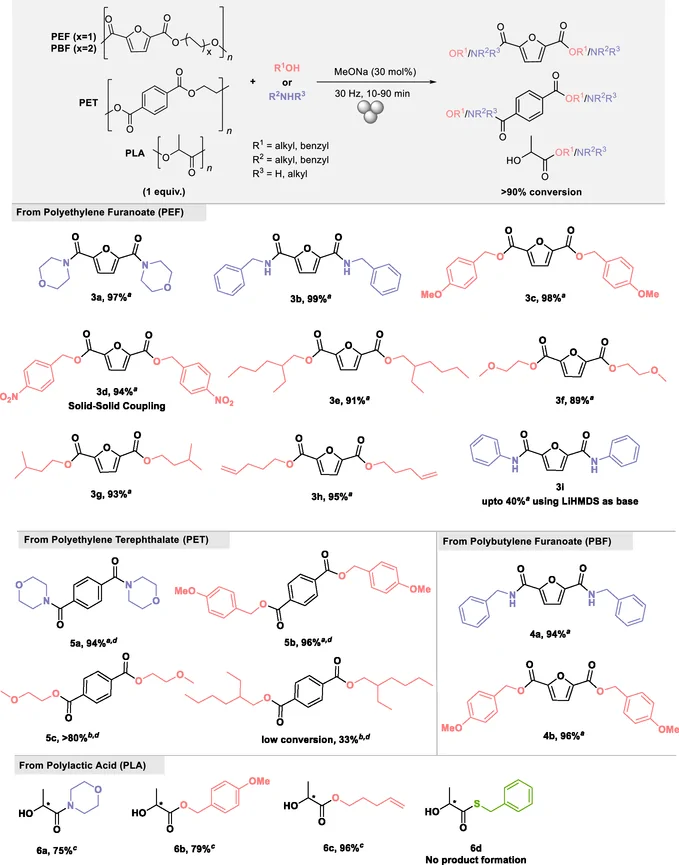
Substrate scope for the mechanochemical upcycling of PEF, PBF, PET, and PLA polyesters. Reaction conditions: 10 mL stainless steel (SS) jar, two 10 mm SS balls, 60–90 min reaction time, and 2.0–2.5 equiv. alcohol or amine (except **2d**, **2e**, and **2g**, where an excess was used; see Supporting Information) for reactions of PEF, PBF, and PET. 1.0–1.2 equiv. amine or alcohol for reactions of PLA. ^a^Isolated yields. ^b^Conversion of polymer into product. ^c^NMR yields. ^d^Use of one 15 mm SS ball instead of two 10 mm SS balls and additional NaCl (2 equiv.) as inert grinding medium. See Supporting Information for more details.

The simplicity of the alcoholysis protocol led us to evaluate the substrate scope using various alcohols in detail (**2a**–**2h**, see Supporting Information), producing several distinct, unknown, and valuable products from PEF (Figure 2). Notably, the amounts of all substrates were systematically optimized to ensure full conversion of the polymer into its respective transesterification products. Furan‐based diesters can be useful as green plasticizers, green solvents, intermediates for fine chemicals and specialty resins, and coating. We first examined our protocol on solid, *p*‐NO_2_ substituted benzyl alcohol that contains an electron‐deficient, potentially reactive functional group. This substrate could be converted quantitatively and produced bis(4‐nitrobenzyl)furan‐2,5‐dicarboxylate (**3d**) with an excellent isolated yield of 94%. Further applicability of the protocol for PEF upcycling via alcoholysis was demonstrated for aliphatic and long‐chain alcohols to produce bis(2‐ethylhexyl)furan‐2,5‐dicarboxylate, also known as diethylhexyl furanoate (DEHF) (**3e**), and diisopentylfuran‐2,5‐dicarboxylate, also known as diisoamyl furanoate (DIAF) (**3g**), in excellent isolated yields of 91% and 93%, respectively. These compounds are discussed as promising, bio‐based, and less toxic [38] alternatives to phthalate‐based plasticizers [39, 40, 41], PEF alcoholysis was also performed with 2‐methoxy ethanol, producing 89% of bis(2‐methoxyethyl)furan‐2,5‐dicarboxylate (**3f**), which can also be useful as a model compound for mechanistic analysis of PEF depolymerization studies [42]. Next, 4‐pentene‐1‐ol produced di(pent‐4‐en‐1‐yl)furan‐2,5‐dicarboxylate (**3h**, 95%), which shows the selectivity of the reaction in the presence of alkene functional groups, which can be useful for further functionalization of the diester product. A decrease in the conversion of polyester into the desired product **3g** and **3h** was observed when we used low‐boiling point alcohols, likely due to partial vaporization of alcohol substrate during ball milling. In these cases, the conversion was improved upon increasing the amount of alcohol to 4 equiv. Of note, phenol and bulky alcohols such as cholesterol (**2h**) did not give the desired product at all.

**FIGURE 3:**
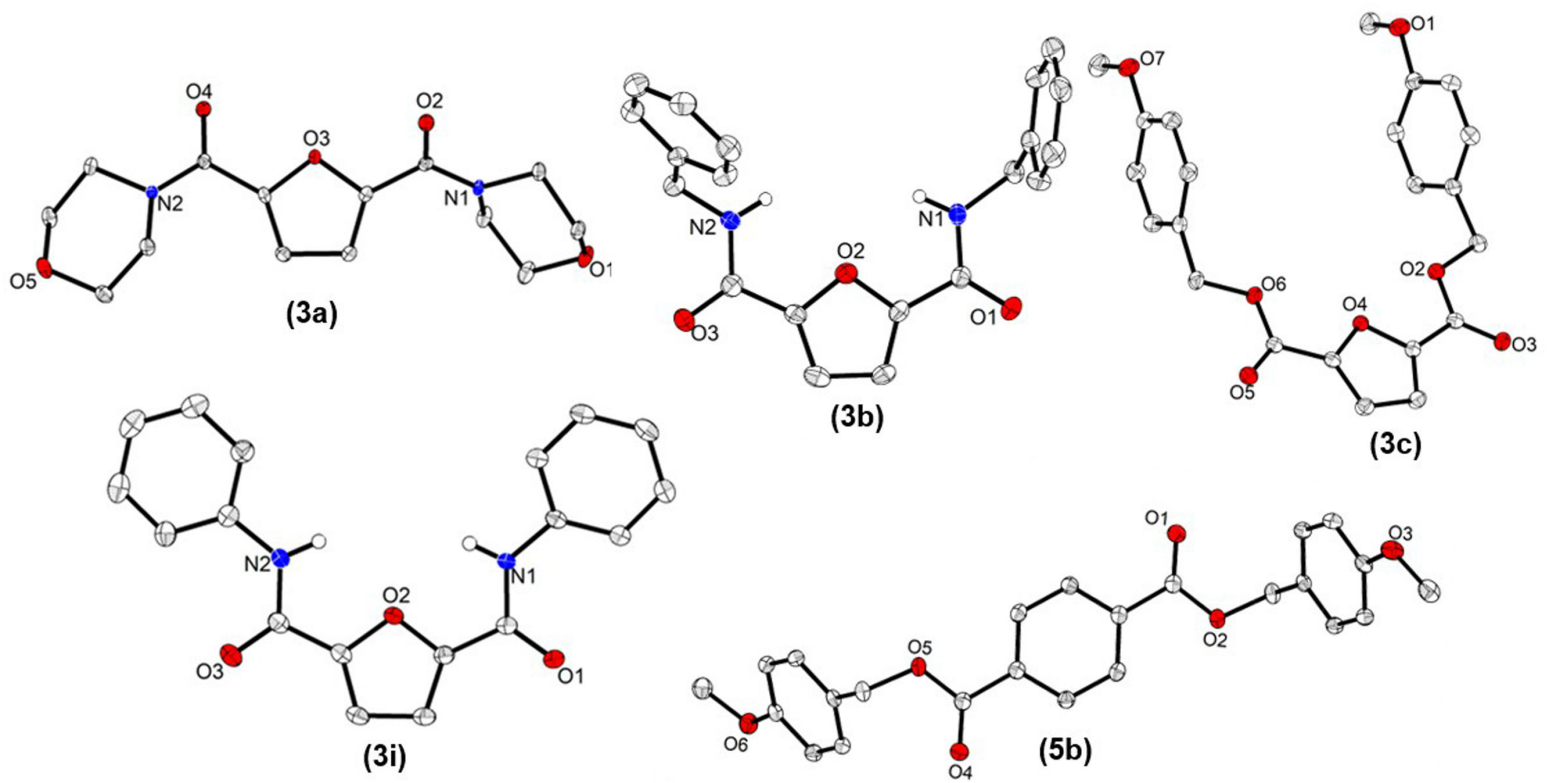
Molecular structures of depolymerization products. Thermal ellipsoids correspond to 30% probability. Hydrogen atoms (except NH) are omitted for clarity.

**FIGURE 4:**
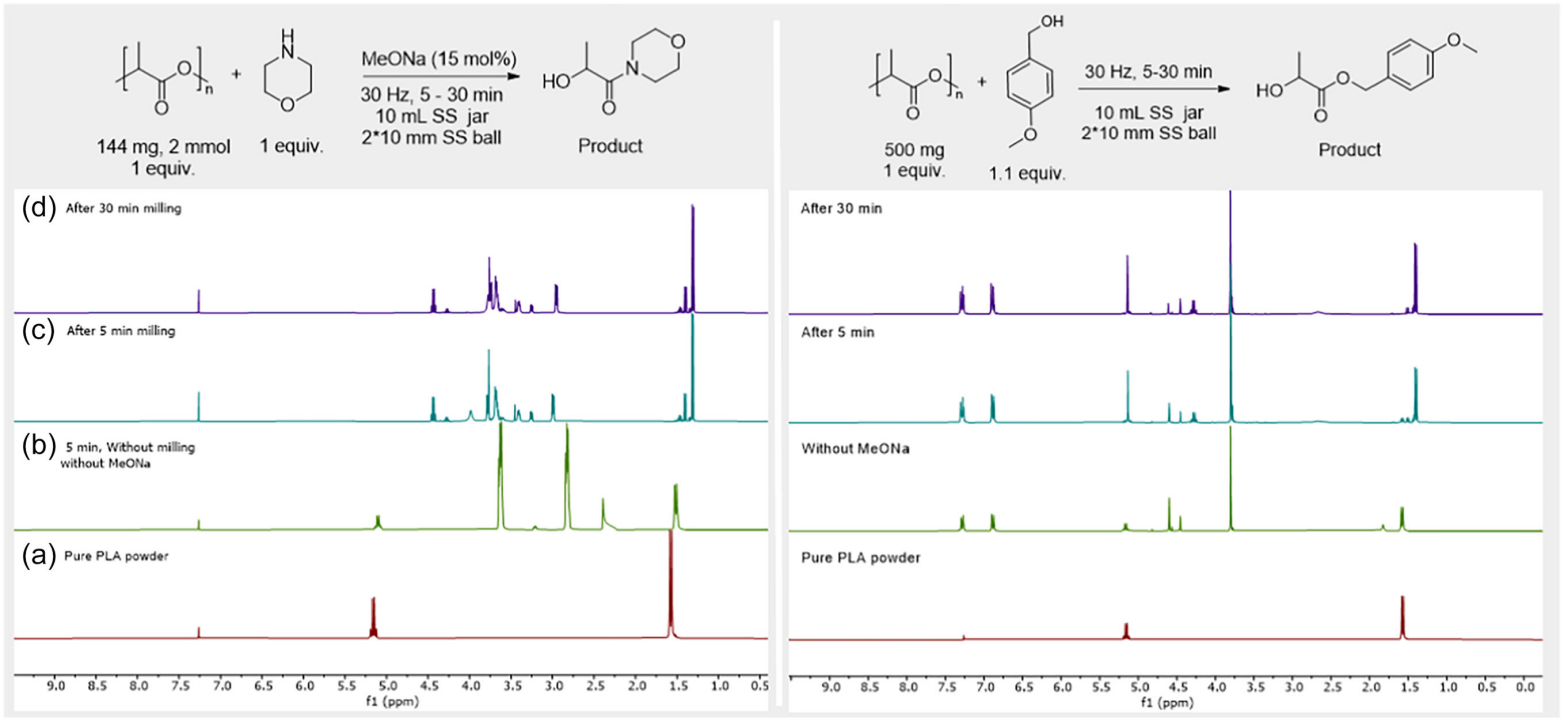
^1^H NMR spectra (297 K, 300 MHz, CDCl_3_) of PLA amidation and alcoholysis control experiments. ^1^H NMR spectrum of (a) pure PLA, (b) reaction mixture without MeONa, (c) reaction mixture with MeONa, showing complete conversion within 5 min, and (d) reaction mixture with MeONa, showing complete conversion within 30 min.

These reaction conditions (Table 2, entry 8) were also found to be suitable for the alcoholysis of the related polyesters PBF, PET, and PLA, producing the corresponding transesterification products in excellent yields. These polyesters and related derivatives have a crucial role for a circular economy and also in biomedical applications due to the presence of the chiral center in PLA. From the alcoholysis of PBF, we have obtained diester **4b** in 96% yield within 60 min using the above‐described optimized milling conditions. In case of PET, both aliphatic and benzyl alcohols were used for the alcoholysis. Of note, when using pure PET granules (192 mg, 1 mmol, 1 equiv.), the addition of NaCl (2 equiv., 2 mmol) as an inert grinding agent and 15 mm SS ball was required due to the hardness of the sample. The reaction of 4‐methoxybenzyl alcohol produced bis(4‐methoxybenzyl) terephthalate (**5b**, 96% yield), and 2‐methoxyethanol converted more than 80% of the polymer into a mixture of alcoholysis products, which contained 90% of the major product **5c** along with some impurities of other byproducts. Surprisingly, a small‐scale PLA alcoholysis reaction with 4‐methoxybenzyl alcohol produced product **6b** in 79% NMR yield within 5–10 min reaction time, followed by completed depolymerization of PLA (Figures 3 and 4). Of note, use of PLA produced complex reaction mixtures of **6a** and **6b**, as indicated by ^1^H NMR spectra, which show the presence of at least two minor byproducts. Using an excess amount of 4‐pentene‐1‐ol (2.5 equiv.), 96% NMR yield of **6c** could be obtained. No formation of **6d** was observed when benzyl thiol was used instead of an alcohol.

### 2.2 Amidation of Polyesters

The optimization of the reaction conditions for aminolytic depolymerization was performed using PEF. Initial trials for PEF amidation with benzyl amine were carried out without using any catalyst, converting up to 60% PEF into depolymerized products, with the corresponding diamide as a major and monoamide as a minor product (Table 3, entry 3 and Figure 2). Both products were isolated using column chromatography and characterized by ^1^H and ^13^C NMR spectroscopy (Figures S25 and S26). To improve the yield of the reaction, ZnBr_2_ was added as a Lewis acid catalyst, which selectively produced 80% diamide product in the presence of NaCl as auxiliary (Table 3, entry 4). Finally, upon using 30 mol% MeONa instead of ZnBr_2_ and without NaCl, all PEF was converted into the corresponding amidation product (Table 3, entry 2), producing N^2^,N^5^‐dibenzylfuran‐2,5‐dicarboxamide (**3b**) in 99% yield after thorough washing of the reaction mixture with water.

**TABLE 3:** Summary of the reaction conditions used during optimization of the mechanochemical amidation of polyesters.^a^

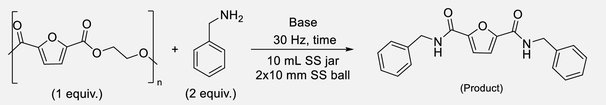					
Entry	Base	Auxiliary	Time, min	PEF conversion into product, %	Yield, %
1.	MeONa (0.2 eq.)	None	60	>90	93
2.	MeONa (0.3 eq.)	None	60	>98	99
3.	None	NaCl (4 equiv.)	90	<60 (mixture of products)	31 (major)
4.	ZnBr_2_ (0.2 eq.)	NaCl (4 equiv.)	90	81%	80

Abbreviation: SS, stainless steel.

a Reaction conditions: PEF (182 mg, 1 mmol per repeating unit, 1 equiv.) and benzyl amine (2 mmol, 2 equiv.) were ball milled in the presence of base at 30 Hz.

This straightforward amidation procedure encouraged us to investigate additional amine substrates (Figure 2). Furan‐based amides are very important as pharmaceutical intermediates for the synthesis of bioactive molecules, and their amide linkage plays an important role in the introduction of heteroaromatic backbones for functional material synthesis and biomaterials [43]. Using the optimized conditions, furan‐2,5‐diylbis(morpholinomethanone) (**3a**) was obtained in excellent yield of 97% using morpholine as a secondary amine partner, which also shows the suitability of the protocol with aliphatic amines. Next, amino acid glycine (**1c**) was used as an amine substrate for the amidation of PEF, but only trace amounts of product were obtained. Aniline (**1d**), as a deactivated aromatic amine, was also tested for amidation; however, this substrate did not work using the standard protocol. However, when using LiHMDS (2 equiv.) as a base instead of MeONa, all PEF was converted, yielding 40% of the corresponding diamide product (**3i**). The mechanochemical upcycling via amidation worked similarly with PBF, PET, and PLA, and we synthesized N^2^,N^5^‐dibenzylfuran‐2,5‐dicarboxamide (**4a**) from PBF, and 1,4‐phenylenebis(morpholinomethanone) (**5a**) from PET, in high yields of 94% and 94%, respectively. Similar to PLA alcoholysis (vide supra), interestingly, a small‐scale PLA amidation reaction with morpholine produced 2‐hydroxy‐1‐morpholinopropan‐1‐one (**6a)** in up to 75% NMR yield within 5–10 min reaction time followed by completed depolymerization of PLA (Figures 2 and 4).

Some of the depolymerization products could also be crystallized from ethanol/acetone mixtures at 7 °C. The corresponding molecular structures were obtained from single‐crystal X‐ray diffraction experiments and are depicted in Figure 3. Bond lengths and angles are in the expected range and confirm the presence of the amidation and alcoholysis products from PEF (**3a–c** and **3i**) and PET (**5b**). For example, the amide units of the amidation products **3a**, **3b,** and **3i** show C═O bond lengths in the range of double bonds (1.217–1.240 Å) and C─N bond lengths in the range of shortened single bonds (1.311–1.358 Å). Similarly, ester groups of compounds **3c** and **5b** show C─O distances in the range of single bonds (1.331–1.342 Å) and shortened double bonds (1.204–1.213 Å), respectively. Of note, whereas PEF‐derived upcycling products crystallizse with syn configuration of the two ester/amide units, dibenzyl phthalate **5b** shows anti orientation of the two ester groups in the solid state.

### 2.3 Control Experiments

To gain further insights into the nature of the mechanochemical transesterification/amidation reactions, we have next performed a set of control experiments. First, in the absence of MeONa, no conversion of the polymer was observed using morpholine and 4‐methoxybenzyl alcohol, respectively (Figure 4). This indicates that this base is required to either deprotonate the alcohol/amine or activate the polyester component through nucleophilic attack at the carbonyl group under these rather mild reaction conditions. This is in line with previous reports of catalytic use of basic additives in the transformation of esters, e.g., for polyester alcoholysis and for transesterification of vegetable oils in solution [44, 45].

Next, selective product formation is already observed after short reaction times of around 5 min. With time, selectivity remains unchanged, and only further conversion of substrates takes place, suggesting the absence of oligomeric depolymerization intermediates (Figure 4) [46, 47].

### 2.4 Upscaling and Application for Upcycling of Packaging Waste

Applicability of the current protocol was also explored for large‐scale upcycling reactions under similar mechanochemical reaction conditions using a 25 mL SS jar and two 15 mm SS balls for 2.50 g PEF, 1.92 g PET, and 2.00 g PLA polymers and alcohols. In all cases, all the polymers were fully converted into corresponding transesterification products with 92% yield of DEHF (**3e**) from PEF, 93% yield of bis(4‐methoxybenzyl) terephthalate (**5b**) from PET, and 71%–75% NMR yield of 2‐hydroxy‐1‐morpholinopropan‐1‐one (**6a**) and 4‐methoxybenzyl 2‐hydroxypropanoate (**6b**) from PLA amidation and transesterification, respectively (see Supporting Information, page S32).

**FIGURE 5:**
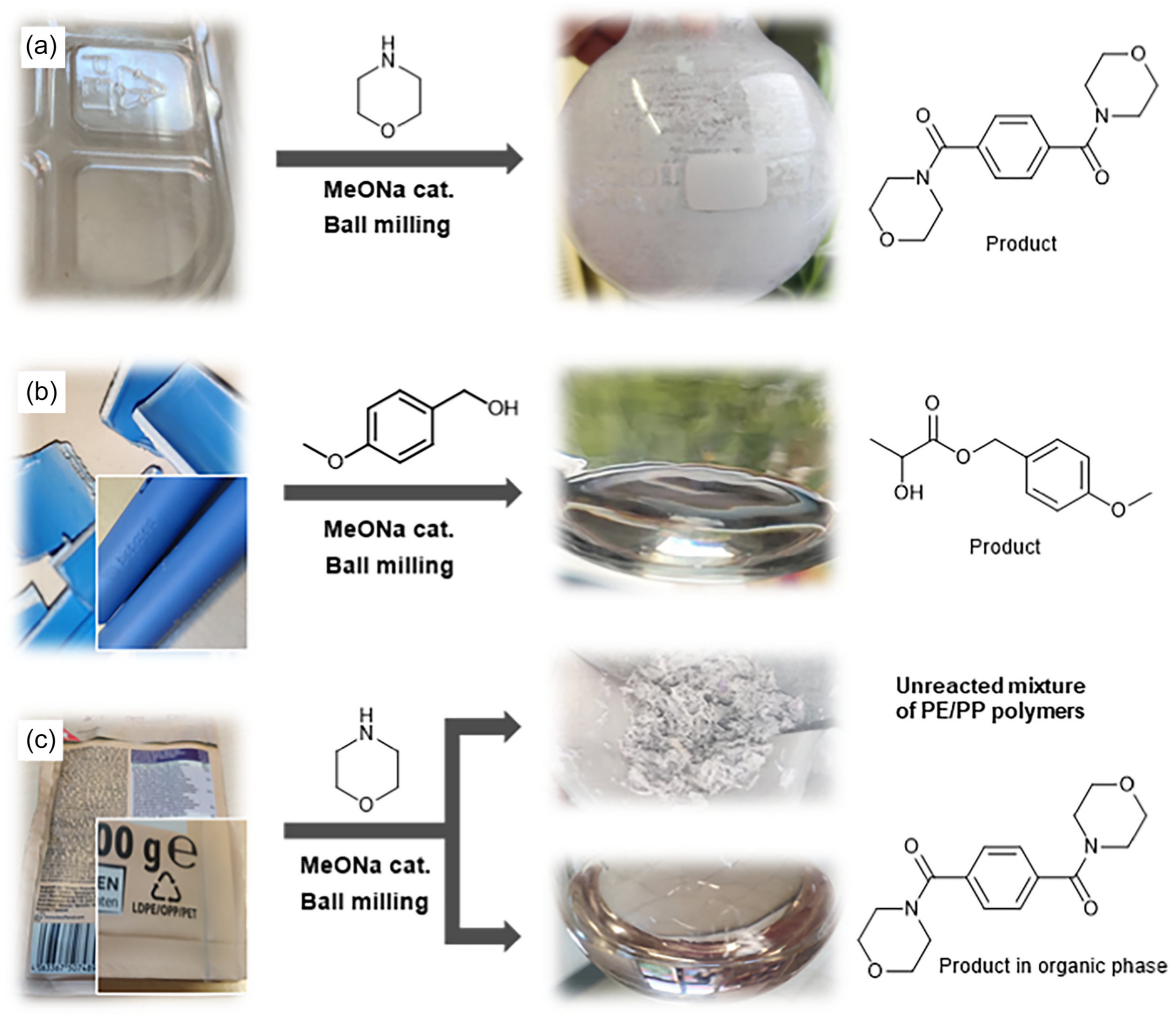
Mechanochemical upcycling of waste packaging materials containing (a) PET (transparent food packaging), (b) PLA (pen), and (c) a polymer blend based on PET, low‐density polyethylene (LDPE), and oriented polypropylene (OPP).

Furthermore, we demonstrated our protocol for the degradation‐upcycling of polyester‐based packaging materials under similar reaction conditions. Using a PET‐based food packaging material (postPET), which according to the SPI Resin Identification Code [48] contained pure PET, a reaction was carried out with morpholine (2.2 equiv.) and 1 g of postPET, producing 1.4 g of the corresponding diamide product **5a** in a yield of 98% (Figure 5a).

The use of a PLA pen (postPLA), containing around 70wt% PLA, in a mechanochemical reaction with 4‐methoxybenzyl alcohol resulted in full conversion into the corresponding alcoholysis product **6b**, obtained as colorless liquid along with some remaining excess amount of alcohol (Figure 5b). Interestingly, the protocol could also be applied for mechanochemical upcycling of a packaging material based on a PET/OPP/LDPE blend. Ball milling of 1.5 g of this waste plastic using morpholine and NaCl as inert surfaces for better grinding of the material resulted in the formation of 220 mg of the corresponding PET amidation product **5a**, which could be readily isolated from dichloromethane after aqueous workup of the reaction mixture (Figure 5c, see Supporting Information for more details).

## 3 Conclusion

In summary, we have presented a versatile mechanochemical protocol for the highly efficient and selective depolymerization‐upcycling of bio‐ and fossil‐based polyesters PEF, PBF, PET, and PLA that uses catalytic amounts of MeONa. Whereas reactions in the presence of amines yield synthetically useful diamides, use of alcohols results in the formation of diesters, some of which show potential for use as sustainable, less toxic alternatives to phthalate‐based plasticizers. The solid‐state upcycling process presented in this paper is readily scalable to gram scale and can also be transferred to the use of post‐consumer plastics such as pure and mixed PET‐based packaging materials or PLA consumer products without any loss of yield or selectivity. We are convinced that these results will make an important contribution to the implementation of sustainable and resource‐efficient recycling and upcycling of widely used polyesters. Current work is focusing on transferring the mechanochemical approach presented here to other bio‐ and fossil‐based polycondensates.

## Funding

This work was supported by the Leibniz‐Gemeinschaft (K522/2023).

## Conflicts of Interest

The authors declare no conflicts of interest.

## Supporting information

The authors have provided detailed experimental, spectroscopic, and crystallographic data, as well as polymer characterization data (NMR, SEC, TGA, and DSC) and cited additional references within the Supporting Information [49, 50, 51, 52, 53, 54, 55].

## Data Availability

The data that support the findings of this study are available from the corresponding author upon reasonable request.
