# Supplementary material for: A Solvent‐Free Approach for the Mechanochemical Upcycling of Bio‐ and Fossil‐Based Polyesters Into Value‐Added Chemicals and Bio‐Based Plasticizers
Source: ChemSusChem. 2026 Aug 31;19(17):e70975. doi: 10.1002/cssc.70975 (PMC13528969; doi:10.1002/cssc.70975)
Supplement: Supplementary file 1 [file CSSC-19-e70975-s001.pdf]

Supporting Information

A Solvent-Free Approach for the Mechanochemical Upcycling of Bio- and Fossil-Based Polyesters into Value-Added Chemicals and Bio-Based Plasticisers

Divya Jain,[a] Nora Jannsen,[a] Riko Siewert,[b,c] Dzmitry Zaitsau,[b,c] Hans-Joachim Drexler,[a] and Torsten Beweries*[a,c]

[a] Leibniz-Institut für Katalyse e.V., Albert-Einstein-Str. 29a, 18059 Rostock, Germany.
Email: torsten.beweries@catalysis.de
[b] Universität Rostock, Lehrstuhl für Technische Thermodynamik, Albert-Einstein-Str. 2, 18059 Rostock, Germany.
[c] Department Life, Light & Matter of the Faculty of Interdisciplinary Research at University of Rostock, 18059, Rostock, Germany.

Table of Contents

General Information 2
Starting materials used for mechanochemical upcycling of polyesters 4
Characterisation of PEF and PBF used in this study 5
Characterisation of PET, PLA, postPET, and postPLA used in this study 7
Optimisation of reaction conditions for PEF upcycling 15
General procedure for the mechanochemical upcycling of polyesters 16
Mechanochemical upcycling of post-consumer plastics 27
Large-scale mechanochemical upcycling of polyesters 32
NMR spectra of depolymerisation products 33
Crystallographic details 49
References 52

General Information

Unless otherwise indicated, all commercially available starting materials and solvents were purchased and used as received without further purification. This study uses the same polyethylene furanoate (PEF) and polybutylene furanoate (PBF) as in our earlier work.[1] Polyethylene terephthalate (PET, white granules) was purchased from BLDpharm and polylactic acid (PLA, powder) was purchased from Magerial. Post-consumer PET and PLA based materials (denoted as postPET and postPLA) were used directly from commercially available products (i.e., food packaging for postPET and LDPE/OPP/PET blend and plastic pen for postPLA). Sodium methoxide was synthesised by stirring sodium metal (1 g) with an excess amount of methanol (20 mL) at room temperature to 50°C in an oven-dried Schlenk flask for one hour, followed by drying the reaction mixture in vacuum. After drying, MeONa was stored under Ar atmosphere and used in open air for the reaction. Formation of undesired polyester hydrolysis products by partial hydrolysis of the MeONa catalyst was not observed.

For PEF and PBF, NMR spectra of were recorded on an Avance III 500 NMR spectrometer (Bruker Biospin, USA) at 303 K in $CDCl_3$/TFA-*d* (v/v = 4:1) as the solvent. Chemical shifts are reported in ppm (δ) and were referenced to residual $CHCl_3$ (δ_H = 7.26 ppm).
SEC analysis was performed using a modular SEC consisting of an HPLC pump (1200 series, Agilent Technologies, USA), separation column PL MiniMIX-D (Agilent Technologies, USA), and differential refractometer (Knauer, Berlin, Germany). PFP/$CHCl_3$ (v/v = 1:2) was used as the eluent with a flow rate of 0.3 mL min^{-1} at a temperature of 45 °C. Narrowly dispersed polystyrene samples were employed as a standard for the calculation of the relative molar masses.
DSC analysis was carried out with a Q2000 (TA Instruments, New Castle, DE, USA) in a temperature range of −80 to 250 °C at a scan rate of 10 K min^{-1} using nitrogen as purge gas. The first heating, cooling and second heating runs were recorded. The examined sample amount was 5 mg.
TGA was carried out with a Q5000 (TA Instruments, New Castle, DE, USA) with a heating rate of 10 K min^{-1} in the temperature range of 40–800 °C with nitrogen as purge gas and a sample quantity of 5 mg.

For PET, PLA, postPLA (pen material), and postPET (packaging material), SEC analysis was performed with the same instrument using PFP/$CHCl_3$ (v/v = 1:1) as the eluent with a flow rate of 0.3 mL min^{-1} at a temperature of 45 °C.
Differential scanning calorimetry (DSC) was carried out using a Mettler Toledo DSC 822e with a Huber TC100MT cooler under N2 atmosphere. The samples for DSC measurements were tightly sealed in Al pans. The experimental curves were recorded during cooling (573 - 193 K) scans at 10 K·min^{-1}, followed by heating (193 - 573 K) scans at heating rates of 1 K·min^{-1}, 2 K·min^{-1}, 5 K·min^{-1} and 10 K·min^{-1}.
The thermal degradation of the was investigated using Setaram Labsys DTA-DSC-TGA 1600. The temperature calibration of the device was tested using high-purity indium sample. The deviation in temperature is below 0.5 K. The samples of the studied polymers were studied using constant heating at 10 K·min^{-1} in open aluminum pans.

NMR spectra of depolymerisation products were recorded on AV300 and AV400 spectrometers (Bruker Biospin, USA) at 297 K in DMSO-d_6 as the solvent. Chemical shifts are reported in ppm (δ) and were referenced to residual DMSO-h_6 (δ_H = 2.50 ppm, δ_C = 39.5 ppm). Approximately 20 mg of the respective compound were dissolved in 0.4 mL of DMSO-d_6 and the respective solutions were used for ^{1}H and ^{13}C NMR analysis.

IR spectra were recorded on a Bruker Alpha FT-IR, ATR spectrometer.

Ball milling experiments were carried out with a *Retsch* MM400 (Retsch GmbH, Retsch-Allee 1-5, 42781 Haan, Deutschland) ball mill using 10 mL jar for small scale reaction and 25 mL stainless steel jar for large-scale reaction. ZrO_2-Y (zirconia dioxide stabilised with Yttria) and stainless-steel milling jars (10 mL), ZrO_2-Y (10 mm) and stainless steel (10 mm, 15 mm) milling balls were used as standard milling equipment. Further details about the ball milling equipment used for the optimised reaction conditions are given below.

**Small scale reaction**	
Length of the Jar	49 mm
Diameter of the Jar	19 mm
Diameter of the ball	10 mm
Weight of one ball	4.0 g
Number of balls	Two 10 mm or one 15 mm

**Large scale reaction**	
Length of the Jar	≈58 mm
Diameter of the Jar	26 mm
Diameter of the ball	15 mm
Weight of one ball	13.1 g
Number of balls	2

Starting materials used for mechanochemical upcycling of polyesters

Polyesters used in this study

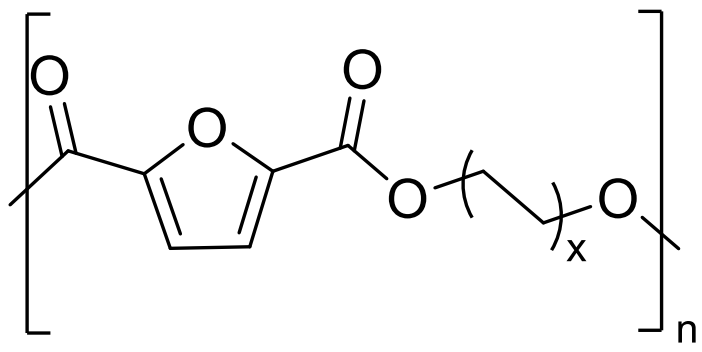

Polyethylene furanoate, PEF (x=1)
Polybutylene furanoate, PBF (x=2)

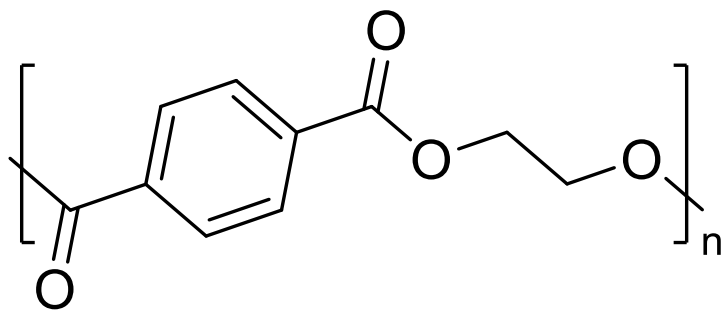

Polyethylene terephthalate (PET) and post-consumer PET-based packaging material (postPET)

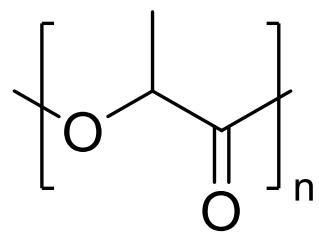

Polylactic acid (PLA) and post-consumer PLA-based material (postPLA)

Amines used in this study

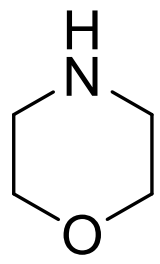

1a
Morpholine

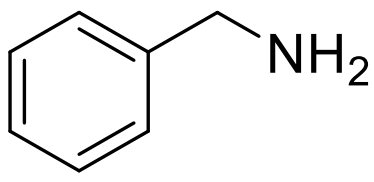

1b
Benzylamine

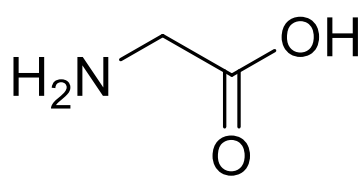

1c
Glycine

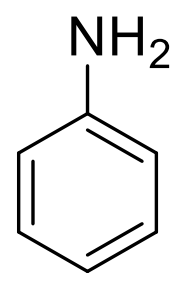

1d
Aniline

Alcohols used in this study

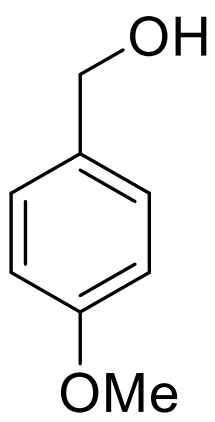

2a
4-Methoxybenzyl alcohol

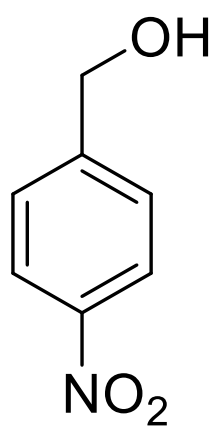

2b
4-Nitrobenzyl alcohol

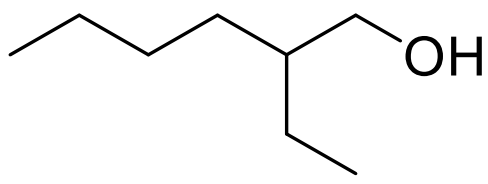

2c
2-Ethylhexan-1-ol

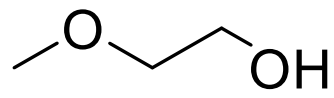

2d
2-Ethoxyethanol

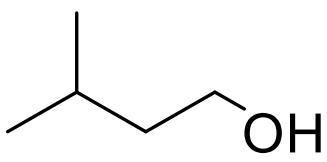

2e
Isoamyl alcohol

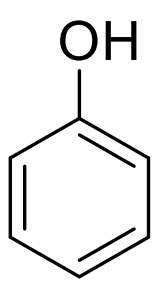

2f
Phenol

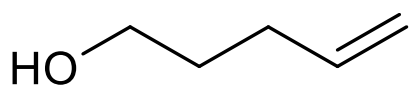

2g
4-Pentene-1-ol

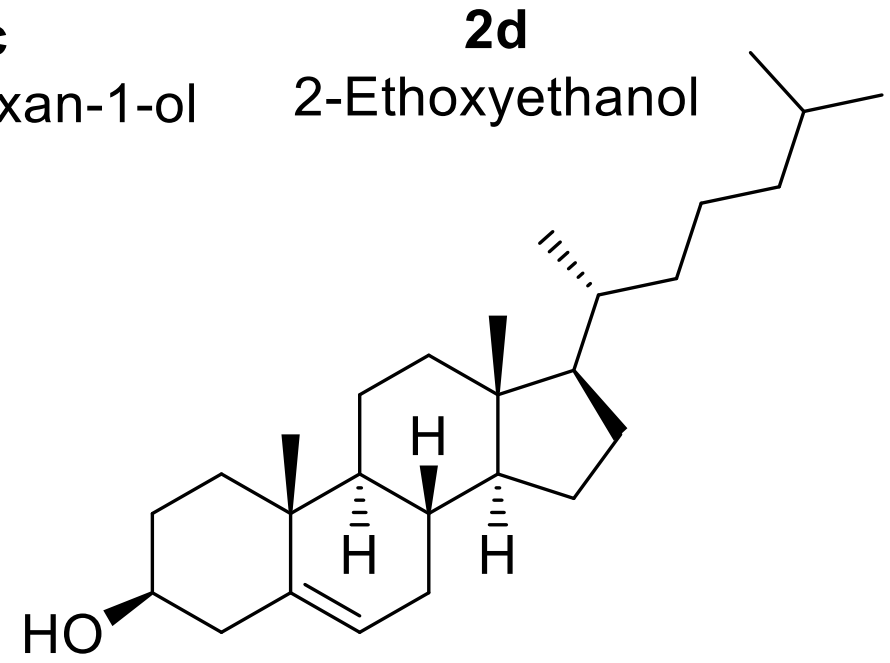

2h
Cholesterol

Figure S1. Polymer materials, amines and alcohols used in this study.

Characterisation of PEF and PBF used in this study

The synthesis of these polymers was reported in a previous study from our group.[1] If not mentioned otherwise, PEF and PBF were used as granules, as obtained from these polymerisation reactions.

PEF

^{1}H NMR ($CDCl_3$/TFA-*d* 4:1, 500 MHz, 303 K): *δ* 3.99 (s, 3H, terminal OC*H*$_3$), 4.72 (s, 4H), 7.32 ppm (s, 2H).
SEC (pentafluorophenol/$CHCl_3$ 1:2, 318 K): M_n = 21,300 g/mol, M_w = 36,900 g/mol, *Đ* = 1.82.

PBF

^{1}H NMR ($CDCl_3$/TFA-*d* 4:1, 500 MHz, 303 K): *δ* 1.95 (s, 4H), 4.00 (s, 3H, terminal OC*H*$_3$), 4.47 (s, 4H), 7.31 ppm (s, 2H).
SEC (pentafluorophenol/$CHCl_3$ 1:2, 318 K): M_n = 33,400 g/mol, M_w = 68,800 g/mol, *Đ* = 2.06.

^{1}H NMR data of PEF and PBF

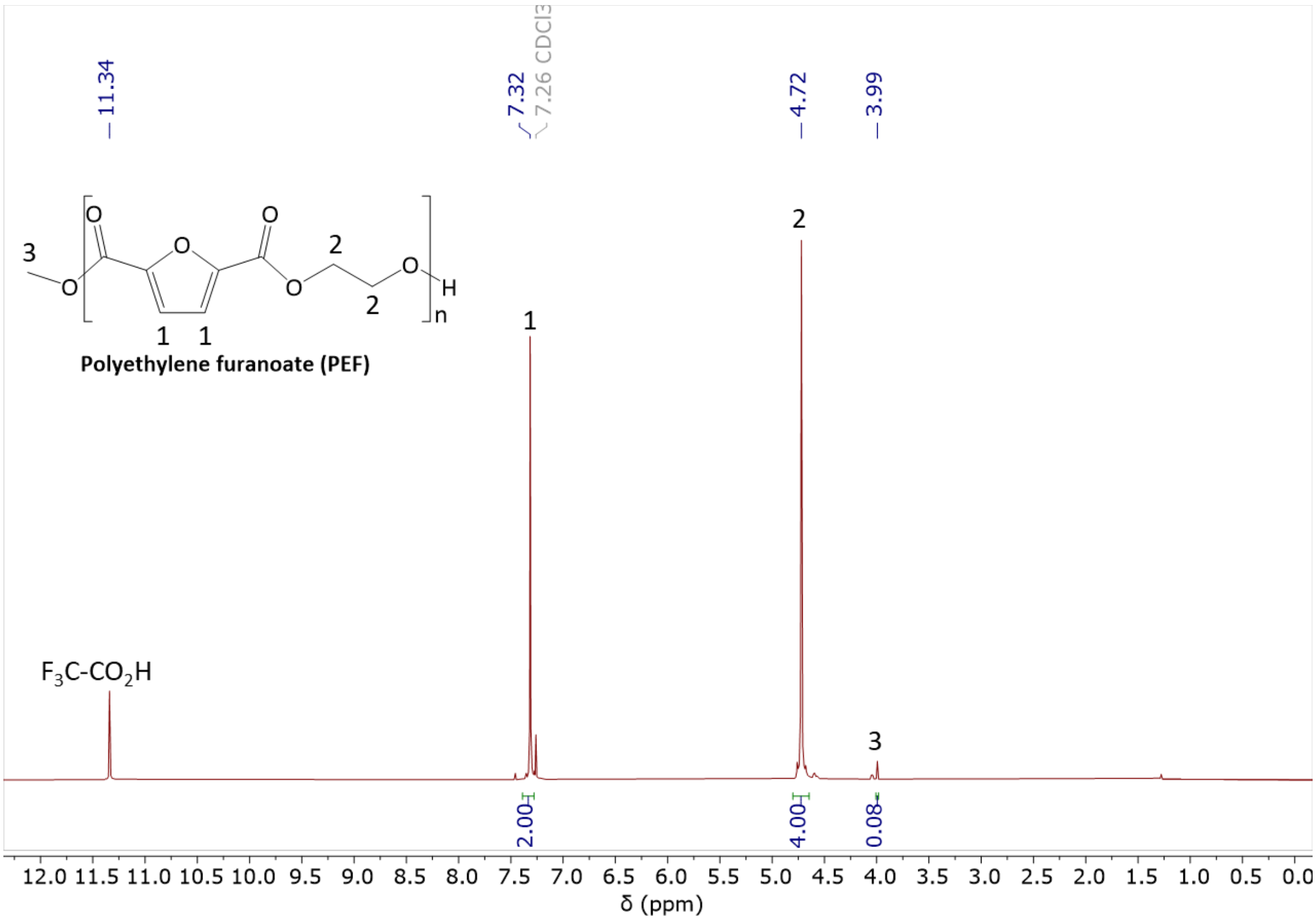

Figure S2. ^{1}H NMR spectrum of PEF ($CDCl_3$/TFA-d_1, v/v = 4:1, 500 MHz, 303 K).

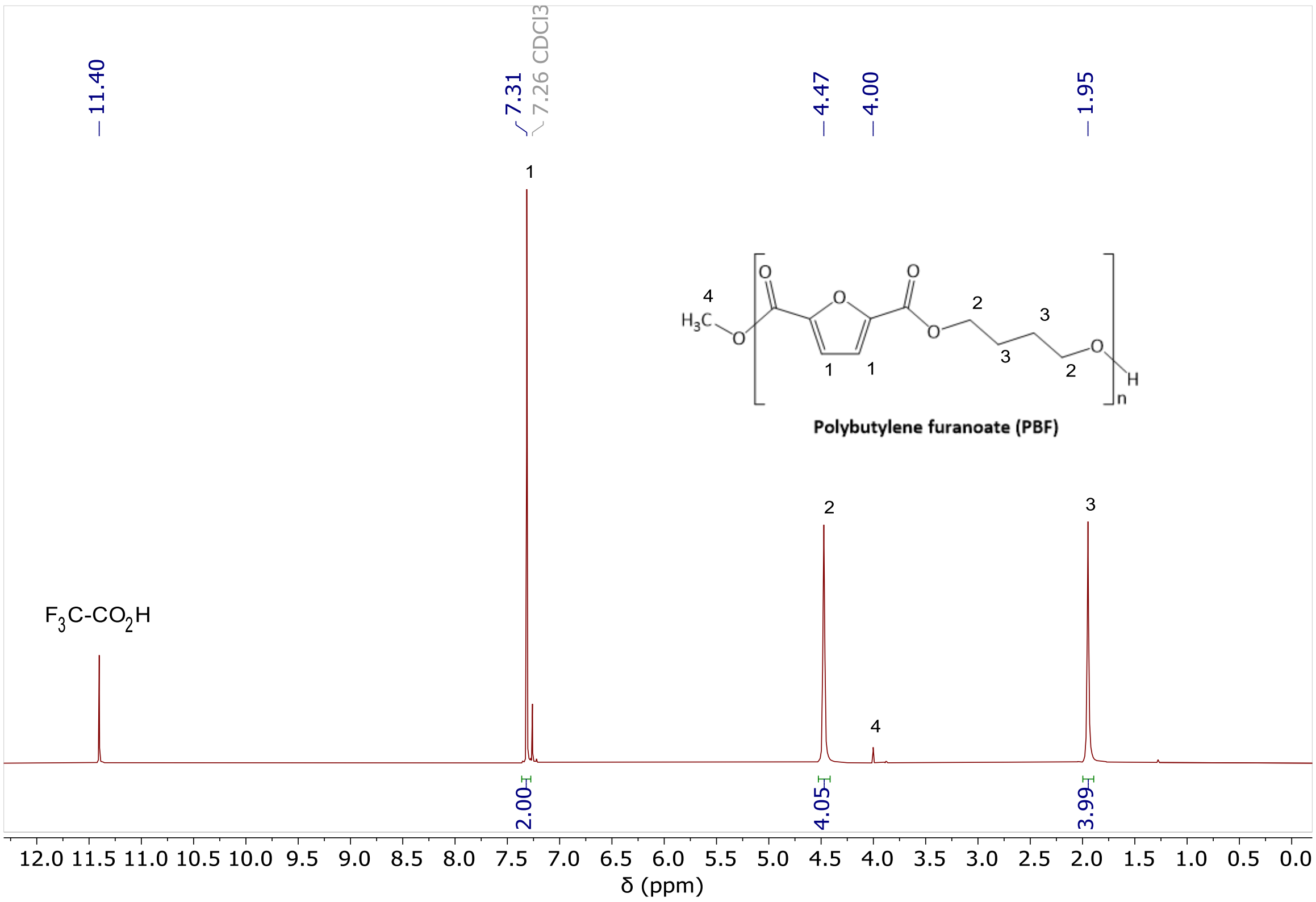

Figure S3. ^{1}H NMR spectrum of PBF ($CDCl_3$/TFA-d_1, v/v = 4:1, 500 MHz, 303 K).

Characterisation of PET, PLA, postPET, and postPLA used in this study

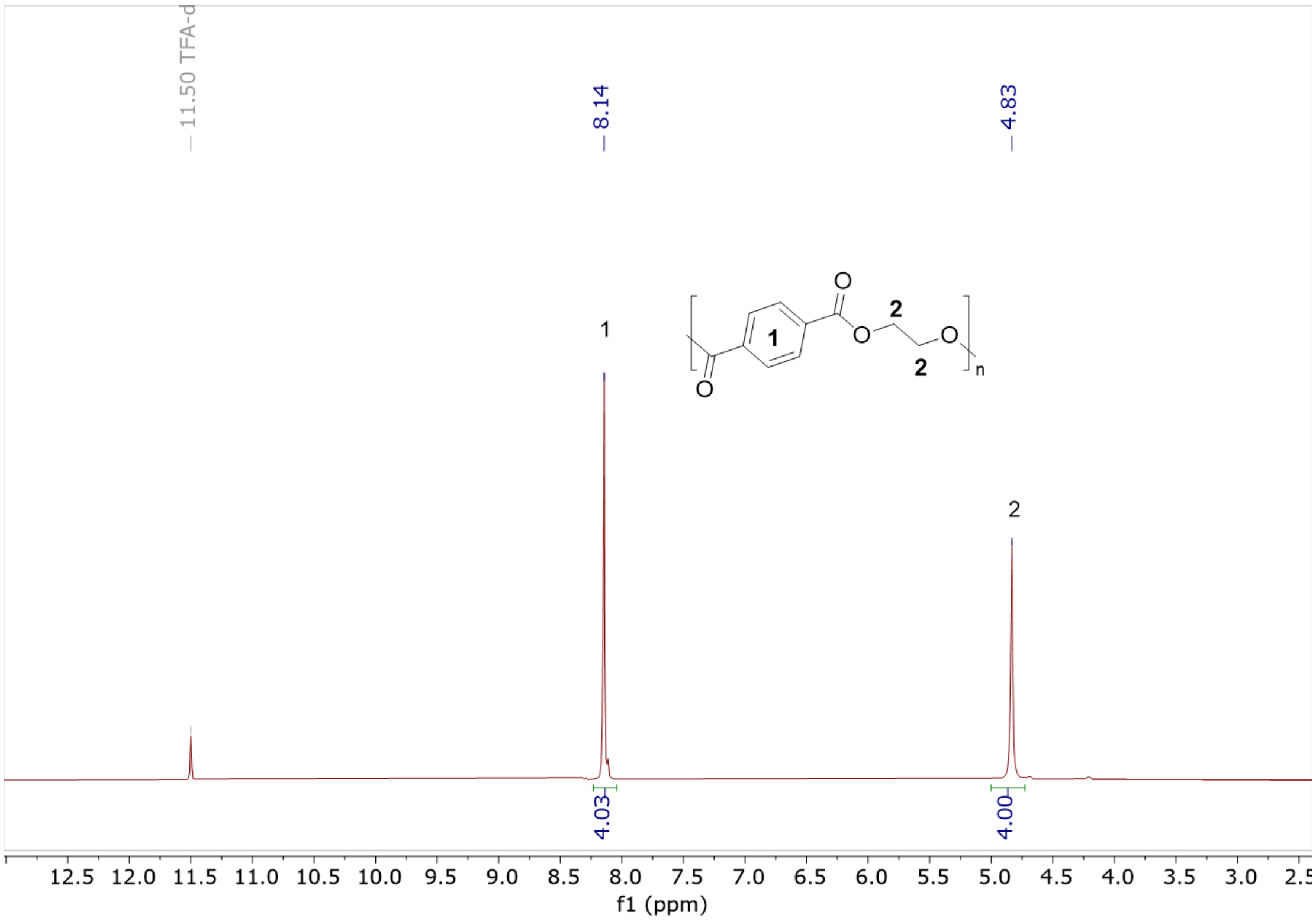

Figure S4. ^{1}H NMR spectrum of pure PET granules (TFA-d_1, 300 MHz, 297 K).

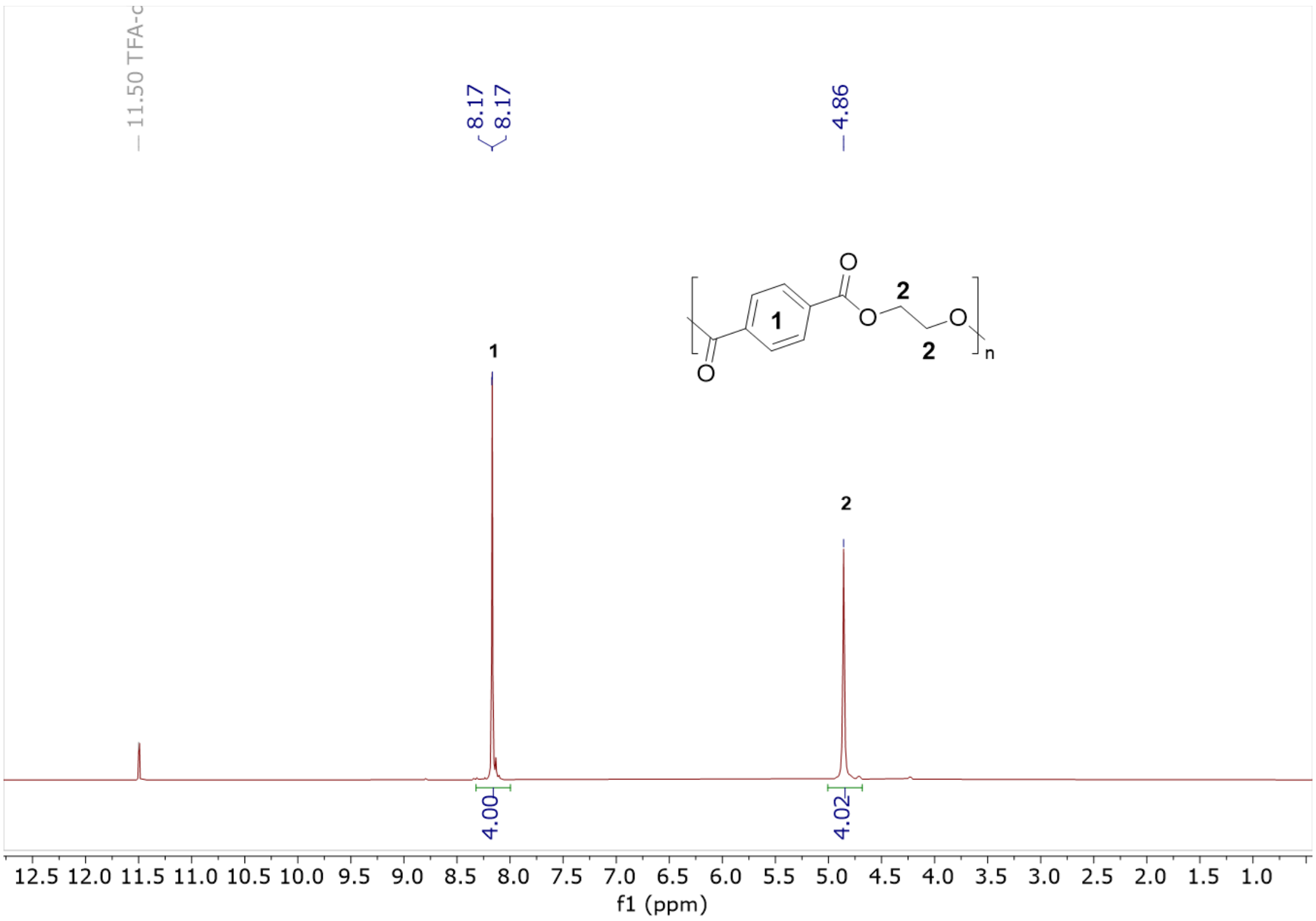

Figure S5. ^{1}H NMR spectrum of postPET (TFA-d_1, 300 MHz, 297 K).

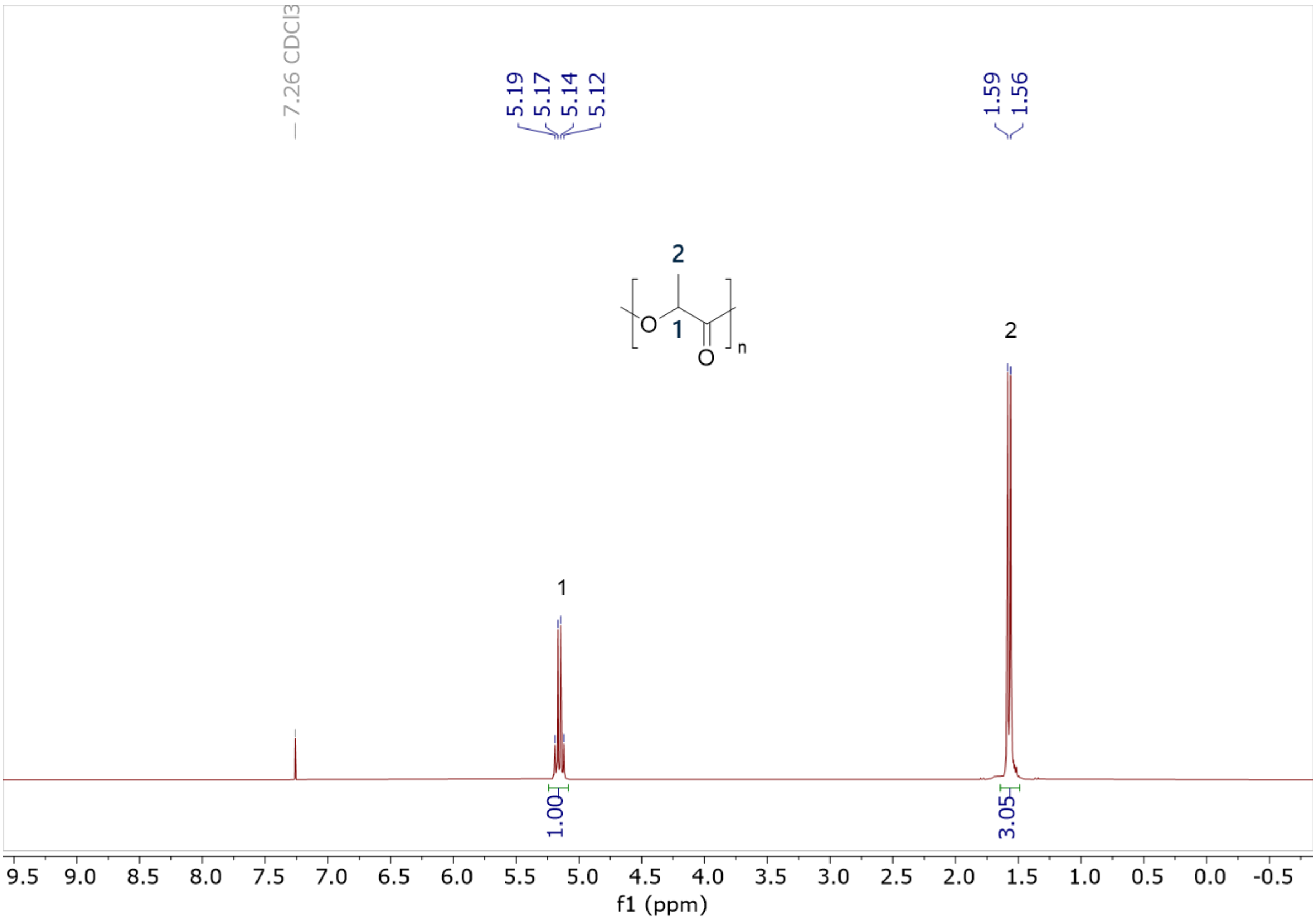

Figure S6. ^{1}H NMR spectrum of pure PLA powder ($CDCl_3$, 300 MHz, 297 K).

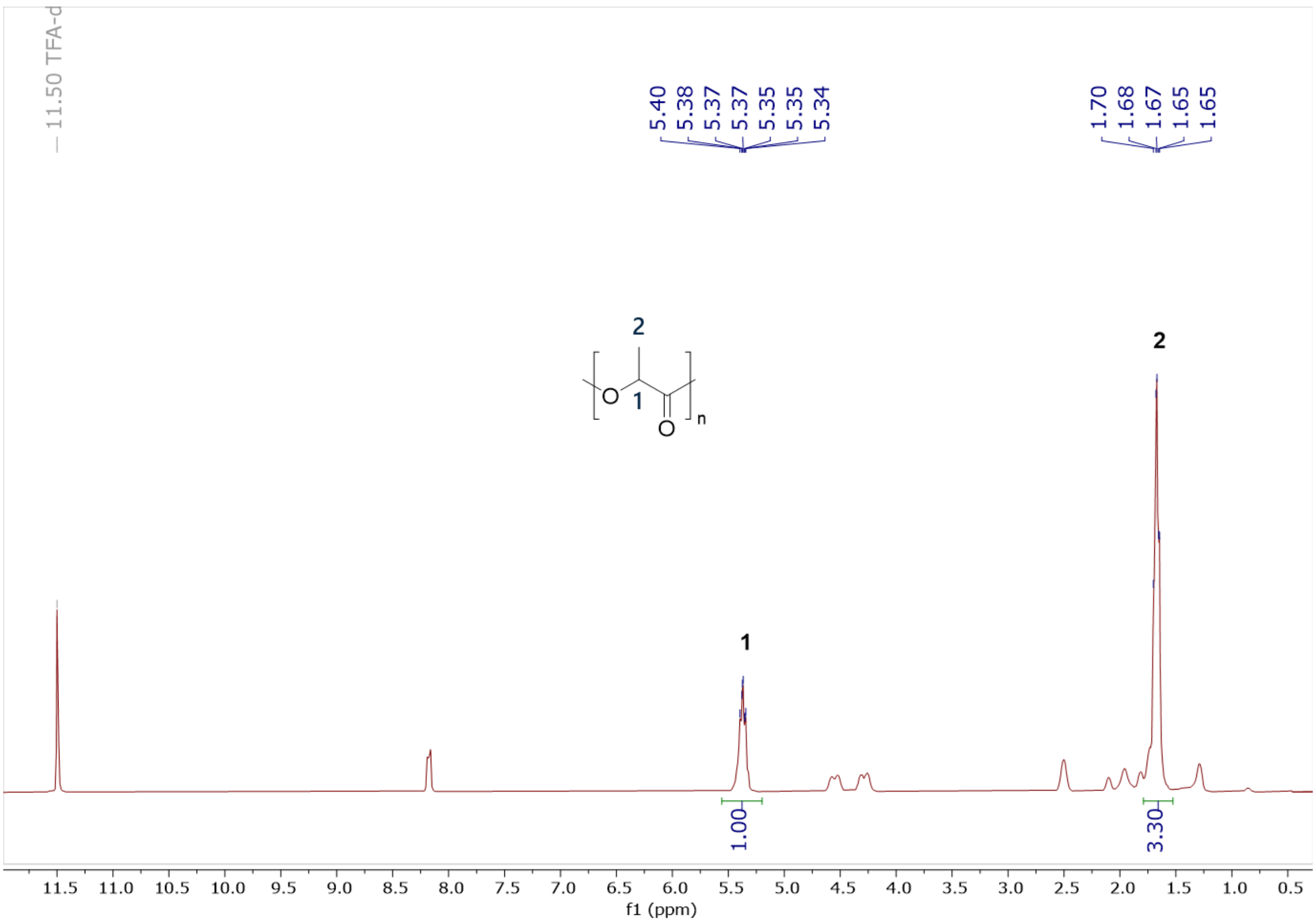

Figure S7. ^{1}H NMR spectrum of postPLA (TFA-d_1, 300 MHz, 297 K).

SEC data of PET, PLA, postPET, and postPLA samples

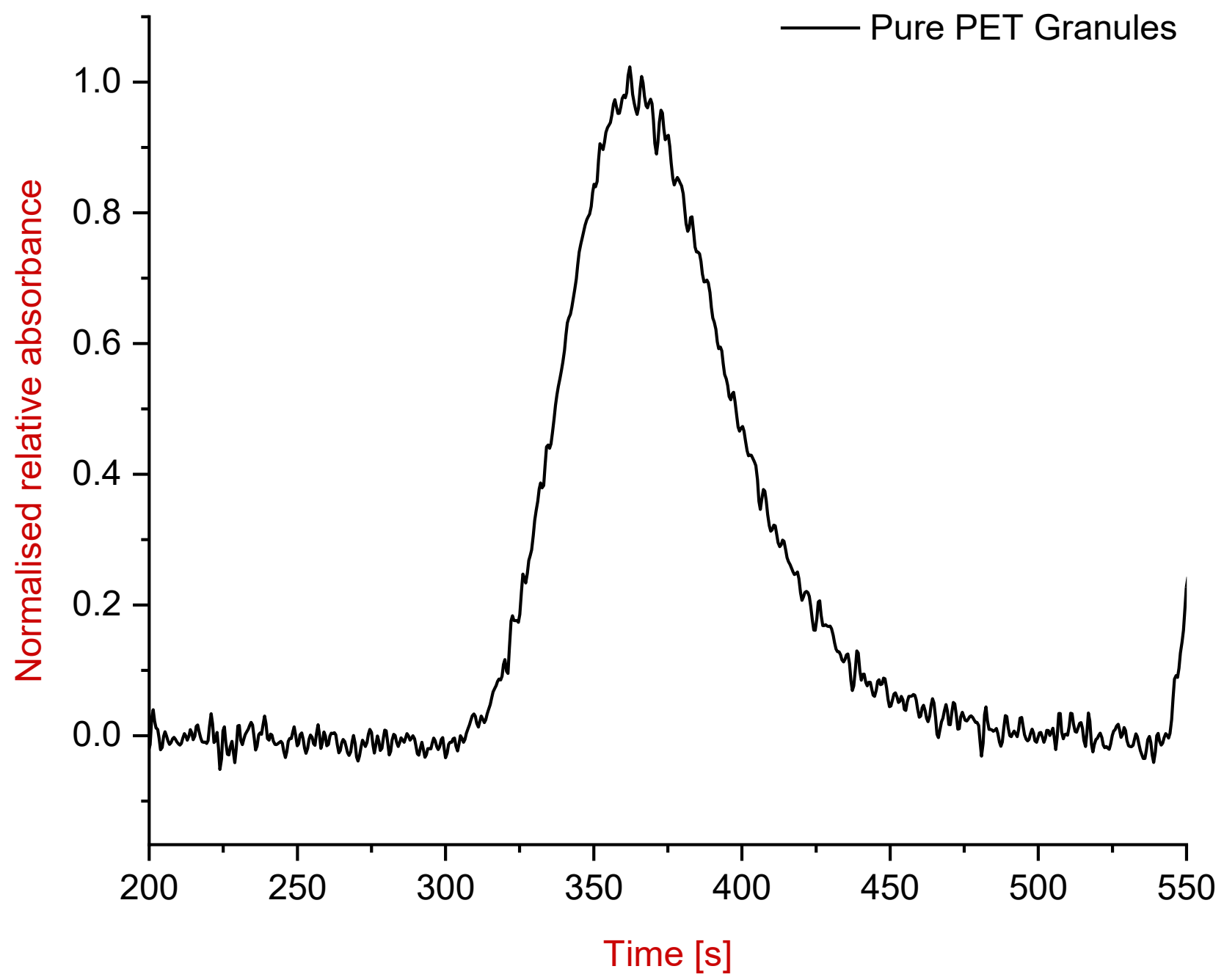

Figure S8. SEC trace of pure PET granules measured in pentafluorophenol/$CHCl_3$ (v/v = 1:1) at 318 K.

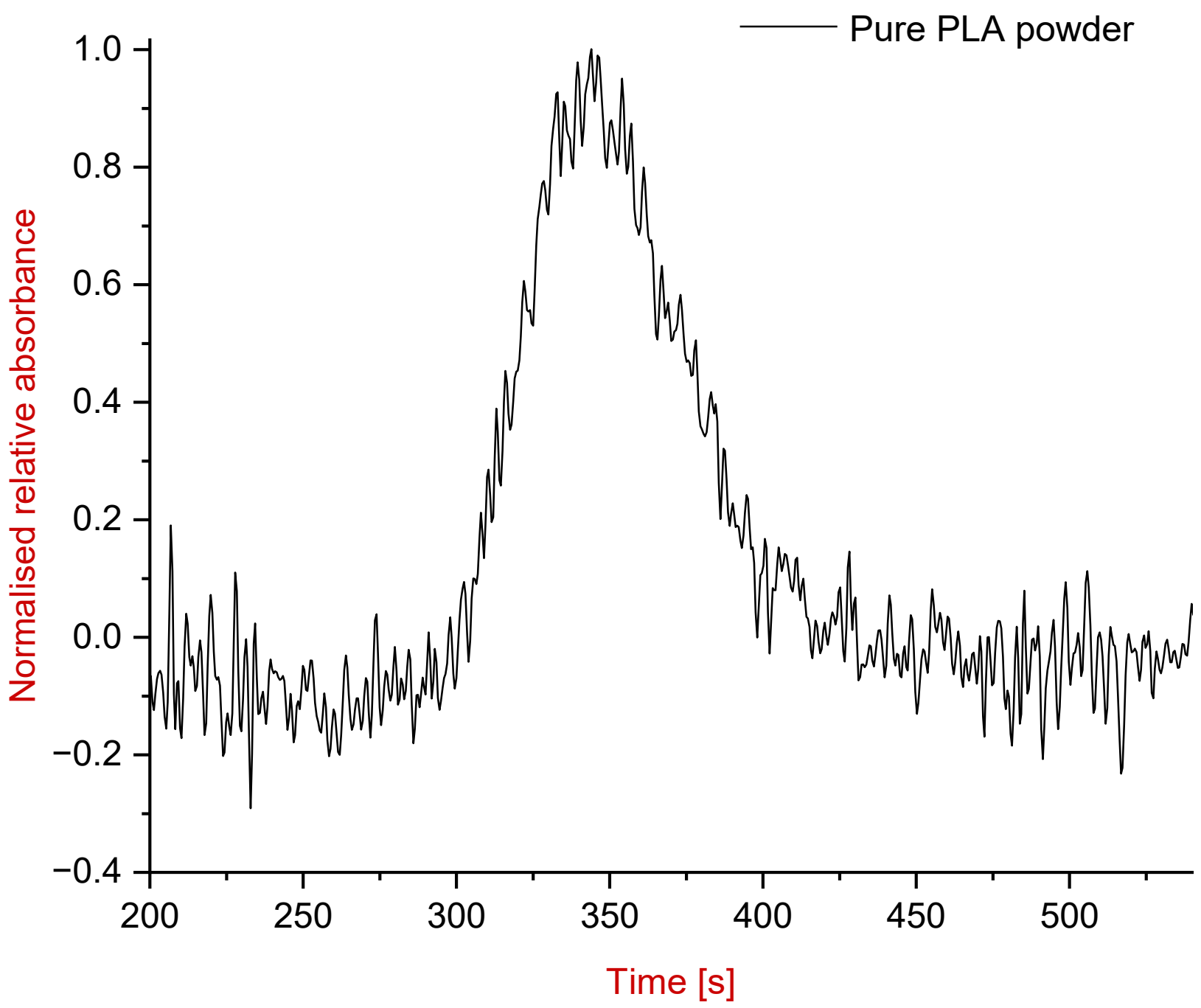

Figure S9. SEC trace of pure PLA powder measured in pentafluorophenol/$CHCl_3$ (v/v = 1:1) at 318 K.

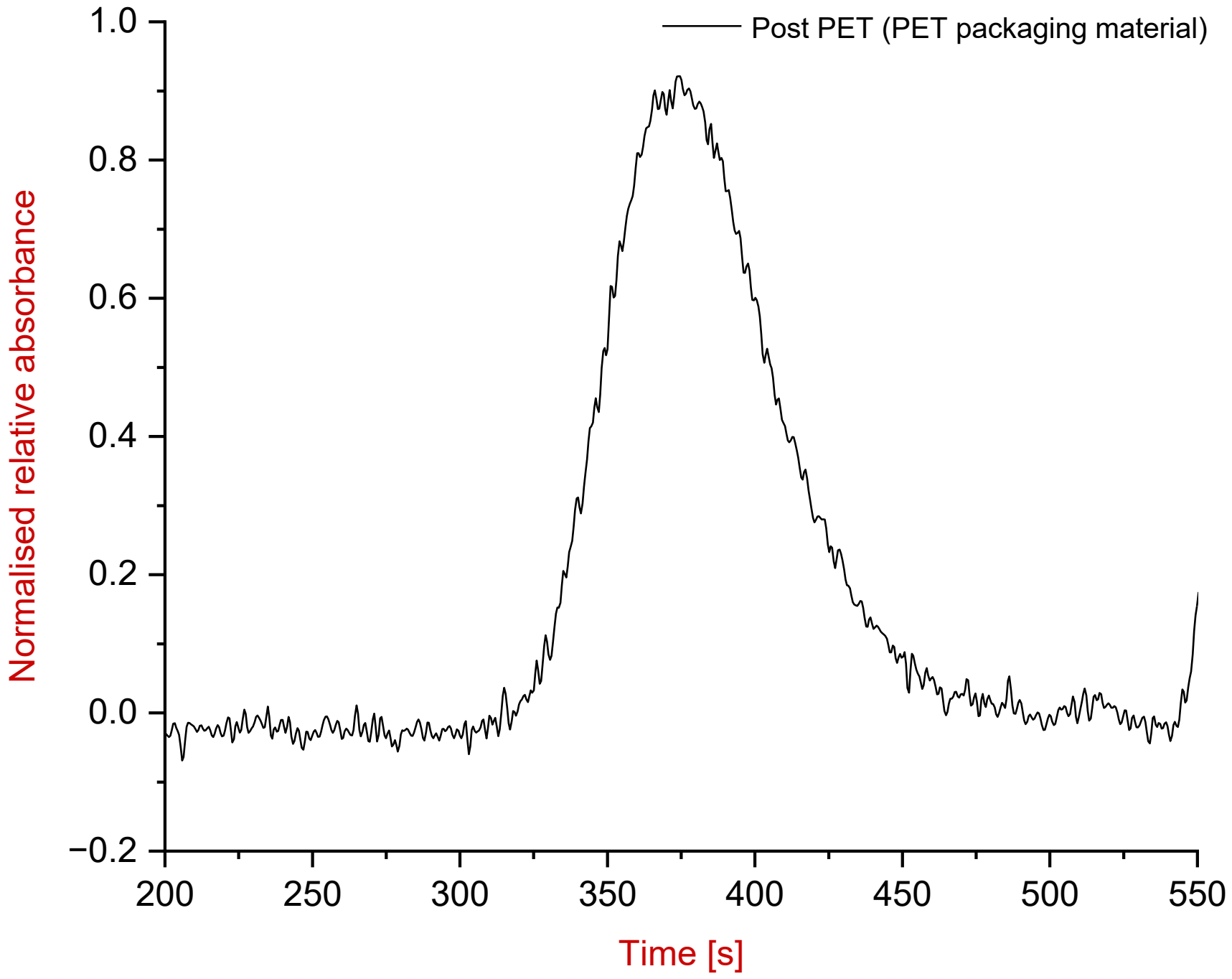

Figure S10. SEC trace of postPET measured in pentafluorophenol/$CHCl_3$ (v/v = 1:1) at 318 K.

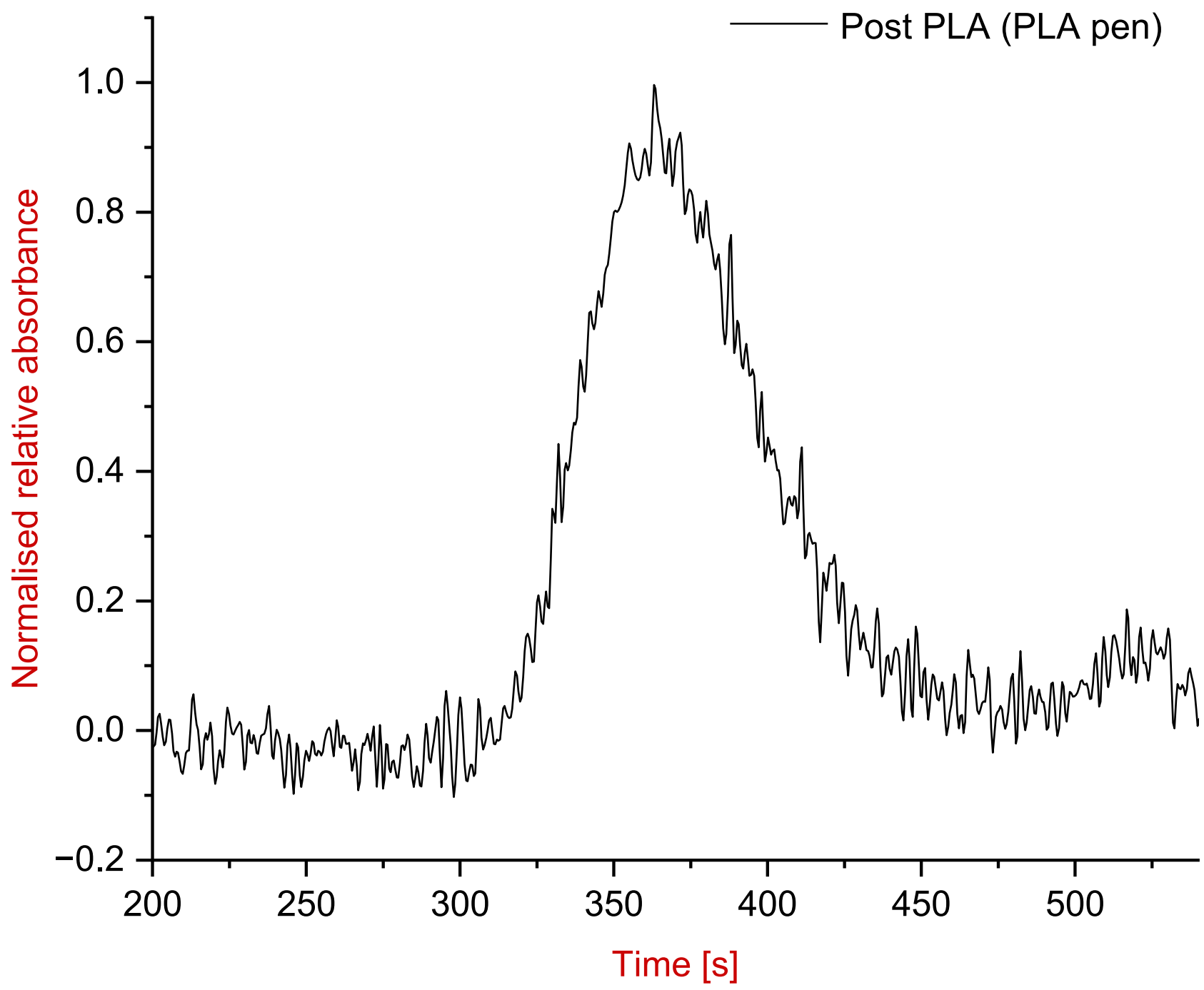

Figure S11. SEC trace of postPLA measured in pentafluorophenol/$CHCl_3$ (v/v = 1:1) at 318 K.

TGA of PET, PLA, postPET, and postPLA samples

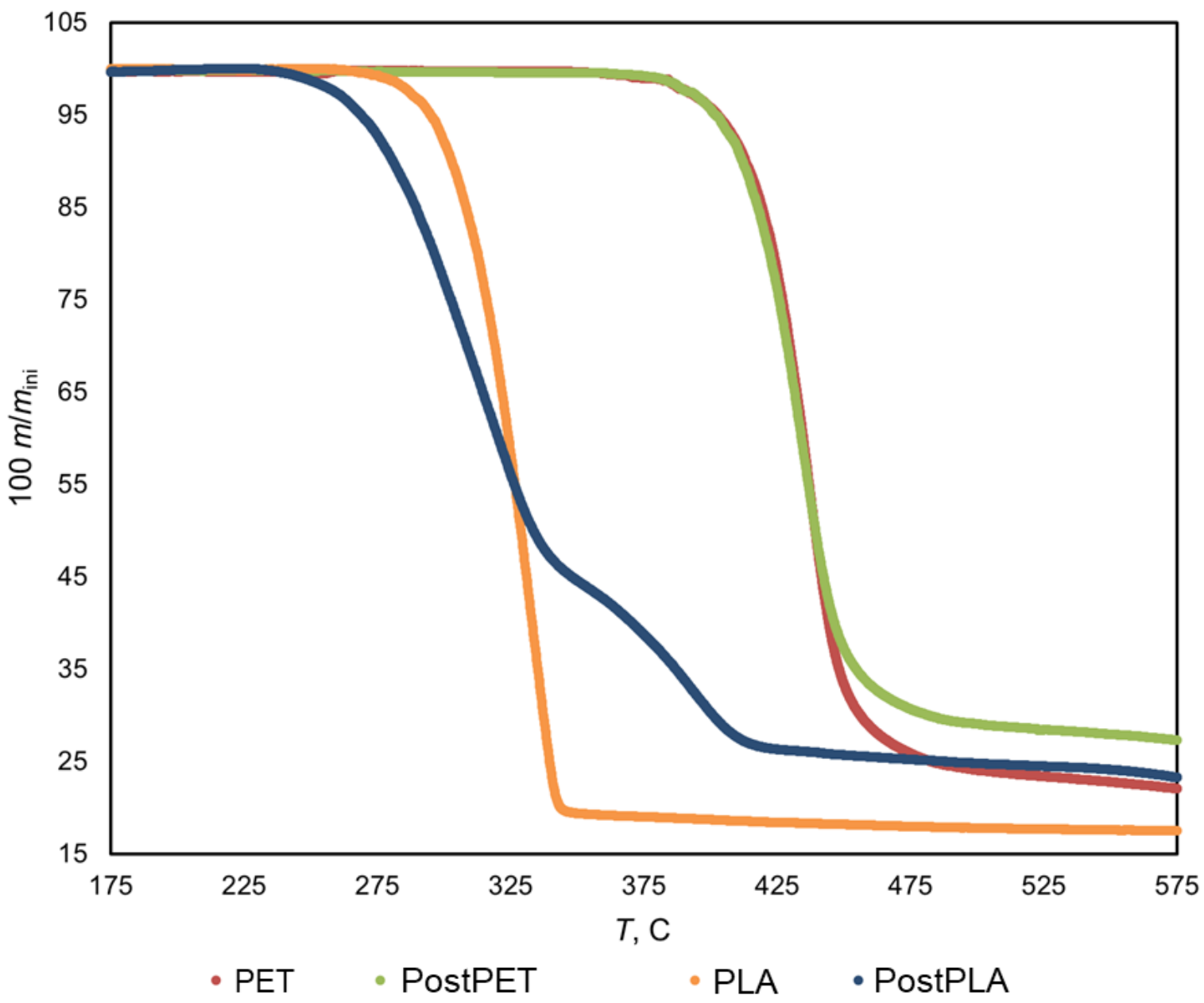

Figure S12. Experimental temperature dependence of mass for the studied polymers.

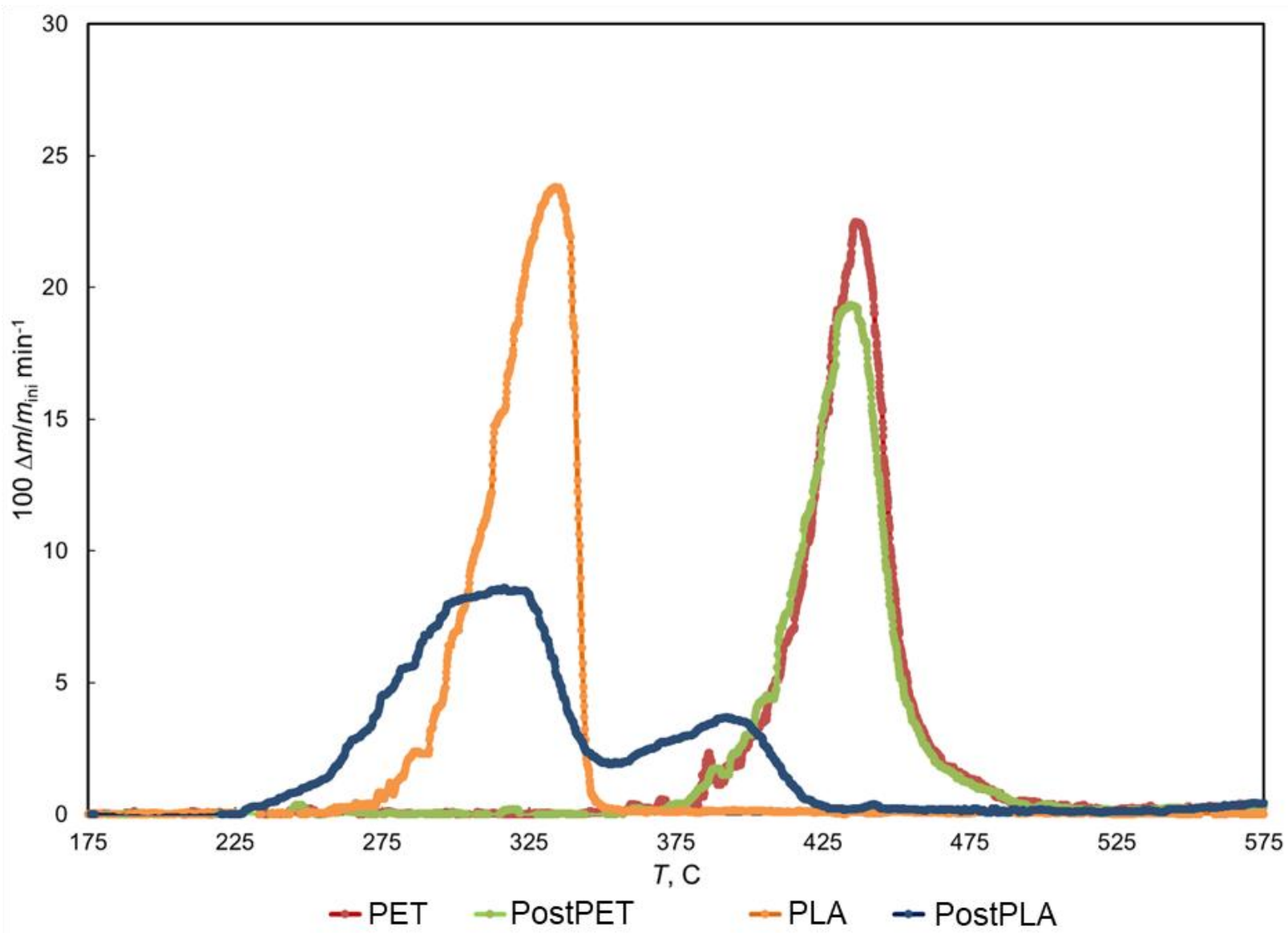

Figure S13. Experimental temperature dependence of mass loss rate for the studied polymers.

As shown in Figure S13, the apparent start of mass loss for both PET and postPET polymers occurs near 350°C. At the same time, due to the fact of kinetic nature of the decomposition process, the beginning of mass loss is dependent on the heating rate. Commonly, the start of mass loss during heating with 10 K·min^{-1} significantly overestimates the thermal stability. Therefore, we have reduced the so-called "decomposition temperature" by 50 °C to ensure the absence of significant chemical changes in a long-term perspective. Apparently, postPLA polymer has a lower thermal stability, and long-term application above 150 °C looks non-reasonable. For the pure PLA polymer sample, application at temperatures below 175 °C can be recommended.

DSC analysis of PET, PLA, postPET, and postPLA samples

Pure PET granules

The thermal behaviour of the PET sample shows the apparent phase transition with the onset temperature T_{trs} = 510.2 ± 0.7 K and $\Delta_{trs}H^o$ = 53.2 ± 0.4 J·g^{-1}. This value corresponds to the high degree of crystallinity of the PET sample.[2] Therefore, no apparent glass transition can be seen during the study. Crystallisation can be avoided by using a cooling rate higher than 1-1.6 K·s^{-1}. Such cooling rates cannot be achieved by the applied experimental setup without additional non-custom calibration. During the first heating at 1 K·min^{-1} the sample was obviously overheated, leading to a non-symmetrical peak with the higher onset temperature. The results of the first run were disregarded.

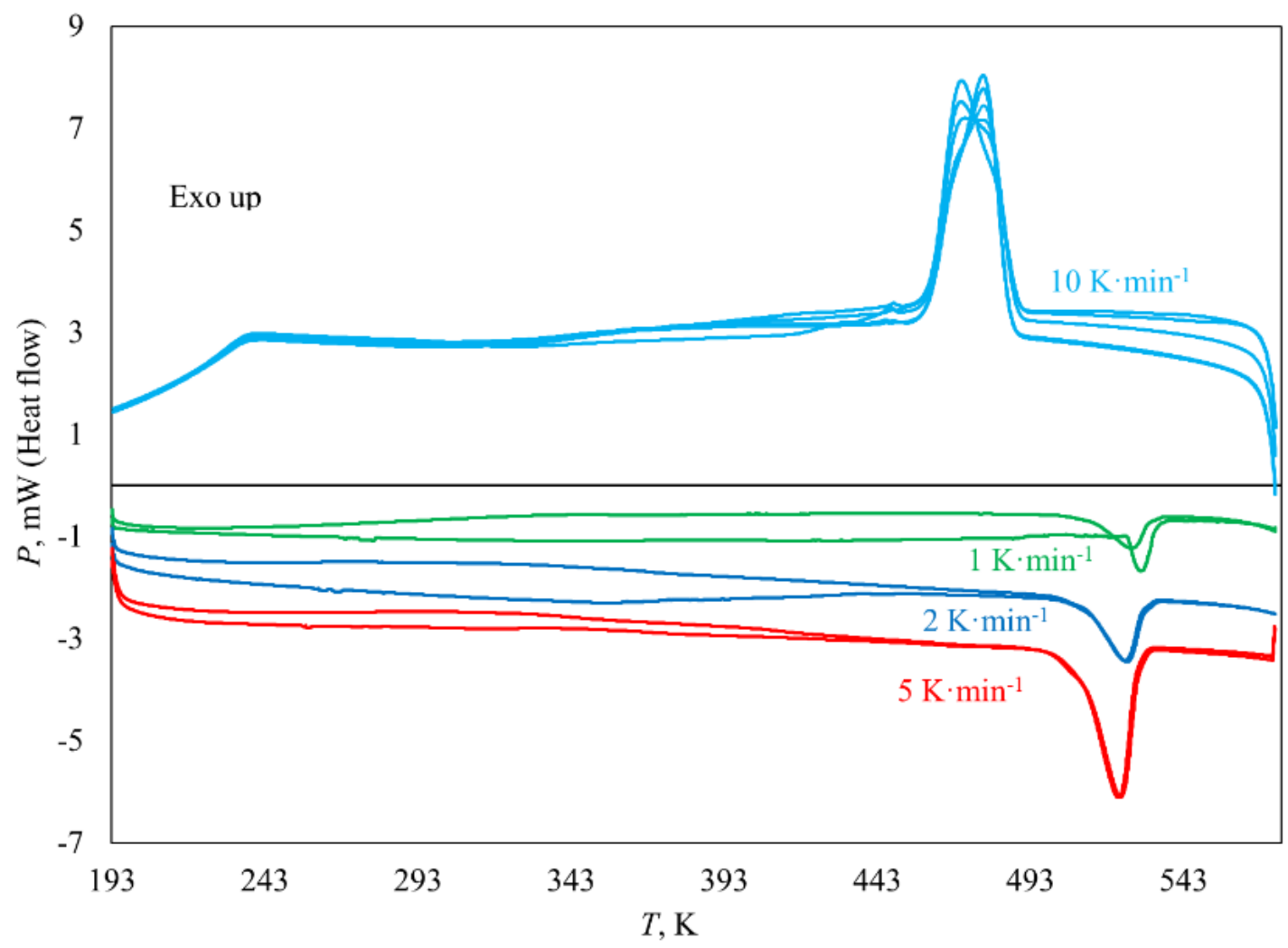

Figure S14. DSC profile of the PET sample.

Post-PET

For the postPET sample in contrast to pure PET polymer two overlapping peaks at 498.1 ± 0.5 K and 508.9 ± 0.5 K are observed. Even by applying the low heating rates, it was not possible to separate the overlapping phase transitions. Additionally, with the decreasing heating rates, the total rate normalized peak area increases. The combined peak area for two peaks determined at 10 and 5 K·min^{-1} resulted in 57.0 ± 1.5 J·g^{-1}, which also supports the idea of high crystallinity of samples cooled with a rate of 10 K·min^{-1}. This value is 7% higher than that of the melting peak observed for PET polymer.

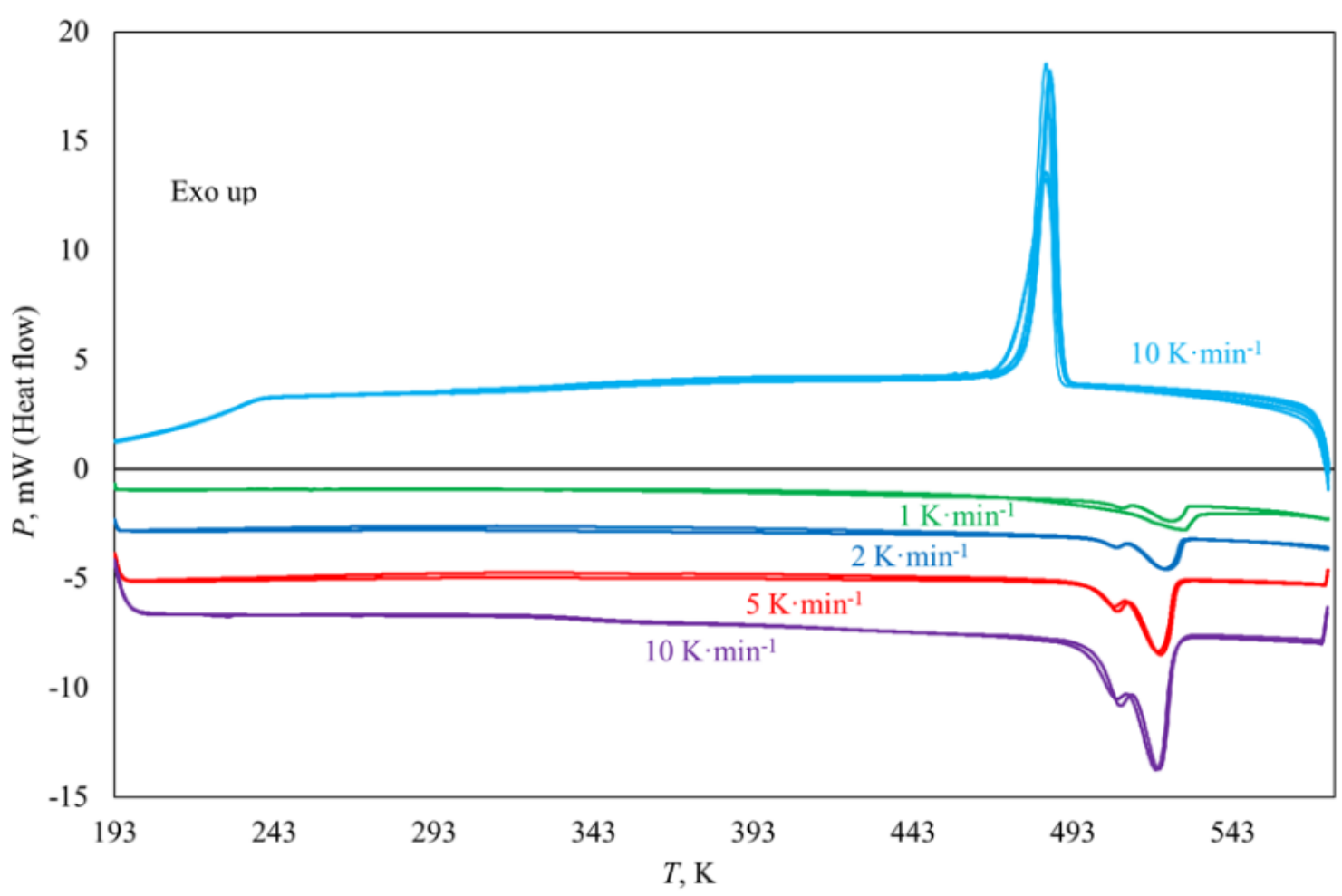

Figure S15. DSC profile of the postPET sample.

Pure PLA powder

The heating and cooling of the PLA sample over temperature interval 193 to 323 K does not reveal any change in thermal behavior of the sample and no obvious transitions were observed. During heating of the sample till 373 K, the glass transition with a large relaxation peak with midpoint 332 K can be seen. Further heating reveals some exothermic increase in the baseline near 373 K, what can be related to crystallization of the sample. Further sequent cooling and heating shows the shift of the glass transition to 328.5 K. During the next step of study till 423 K, no significant change in thermal behavior is observed – the heating and cooling profile is fully reproducible and glass transition is observed at same temperature of 328.5 K. So, it can be stated that PLA is thermally stable for a short time when heated to 423 K.

During the next step, the studied temperature interval was increased to 193 to 473 K. During the first heating, the non-symmetric endothermal peak with an enthalpy of 45 J·g^{-1} and onset temperature 430 K was observed. This peak obviously corresponds to melting of the polymer sample. During consequent cooling and heating no sample crystallization was seen during cooling with 5 K·min^{-1}. At the same time, the narrow spikes can be seen upon cooling of the glass sample corresponding to mechanical sample cracking. During heating, a significant increase in heat capacity jump of glass transition is observed followed by "cold" crystallization of the sample. It should be marked that the onset of the melting peak is shifted in each run by approximately 2 K to lower temperature. This fact can indicate the slow thermal degradation of PLA polymer in liquid state. Further runs with final temperatures of 523 and 573 K apparently show the shift of the onset temperature of the sample melting and a decrease of the enthalpy of the corresponding phase transition.

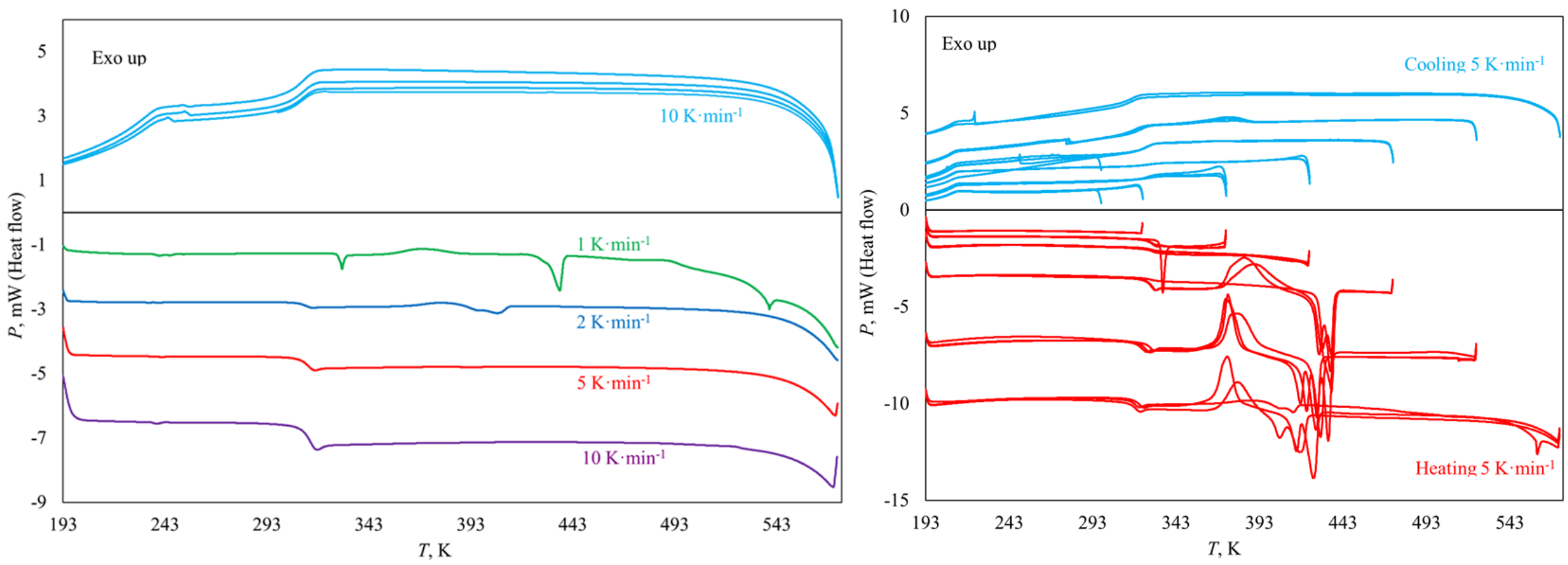

Figure S16. DSC profile of the PLA sample.

Post-PLA from pen material

The thermal behavior for post-PLA sample is very close to PLA sample. But most of the observed transitions are shifted to lower temperature. Thus, the heating and cooling of the postPLA sample over temperature interval 193 to 323 K does not reveal any change in thermal behavior of the sample and no obvious transitions were observed. During heating of the sample till 373 K the glass transition with a large relaxation peak with midpoint 325.6 K can be seen. Further heating reveals apparent "cold" crystallization peak at 360 K. Further cooling and heating shows the shift of the glass transition to 322 K. During the next step of study, heating to 423 K shows the beginning of the melting peak with T_{onset} = 412 K. The glass transition is shifted to lower temperature: midpoint corresponds to 318 K with a slight shift to lower temperature with (approximately 1 K each run). So, with some caution, it can be stated that postPLA is thermally stable during short time exposure to 423 K.

During the next run to 473 K, a double endothermal peak with an enthalpy of 27 J·g^{-1} and onset temperature 425 K was observed. This peak obviously corresponds to melting of the polymer sample. During further cooling sample, crystallization was seen at the temperature 390 K. In contrast to PLA sample, no "cold" crystallization peak can be seen on heating. The onset of melting peak is decreased in each run by approximately 3 K and the obvious change in the ratio of enthalpies in the double peak is observed. One further run with the final temperature of 523 K apparently shows the shift of the onset temperature of the sample melting and a decrease of the enthalpy of the corresponding phase transition. The crystallization peak during cooling is vanished. The midpoint of glass transition after heating to 523 K is shifted to 303 K and no melting peak can be seen on heating. During cooling, the obvious spikes near 270 K related to sample cracking are seen. During the second cooling, the energy of mechanical cracking moved the pan from the pinned position on DSC sensor, what is seen as a vertical dark blue line near 270 K. After the pan moved from the measurement area, no further significant signal could be recorded.

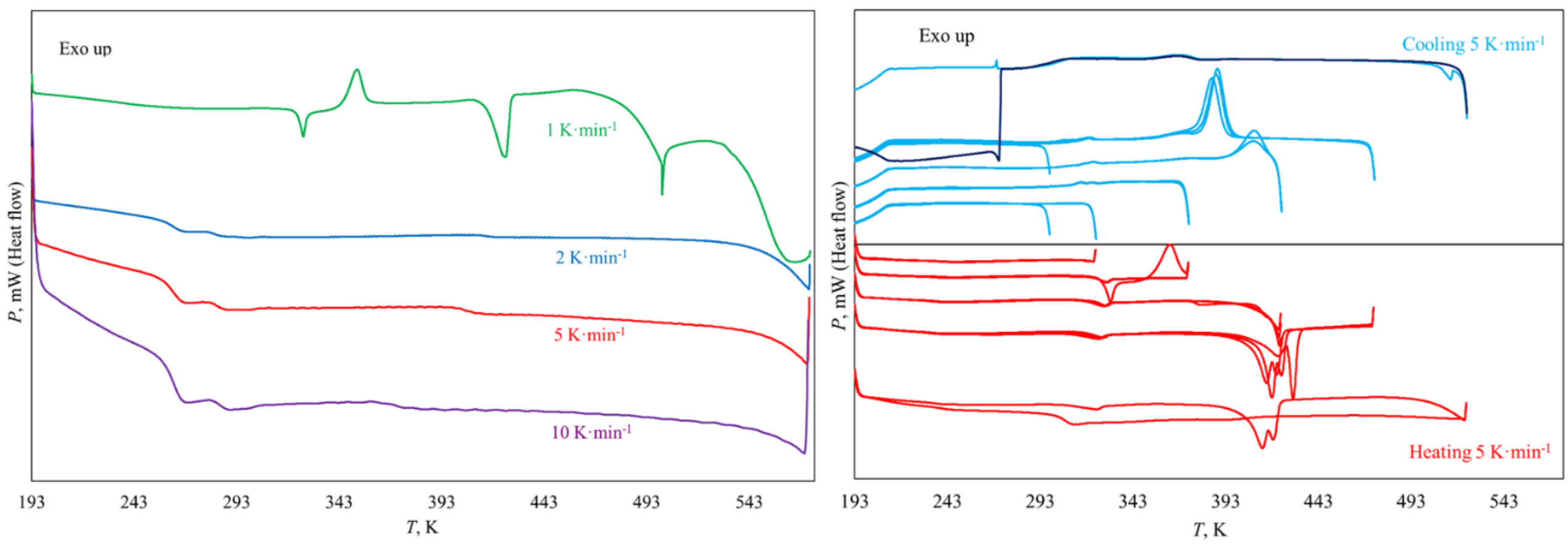

Figure S17. DSC profile of the postPLA sample.

Optimisation of reaction conditions for PEF upcycling

Optimisation of reaction condition was done using polyethylene furanoate (PEF) under mechanochemical condition. 4-methoxy benzyl alcohol and benzyl amine were chosen as the standard substrate for the alcoholytic and aminolytic depolymerization of PEF respectively. Upon optimizing different bases and catalytic systems, only sodium methoxide was found as best catalyst for PEF upcycling under mechanochemical conditions. More descriptions can be found in the manuscript.

Table S1. Optimisation of mechanochemical alcoholysis of PEF.

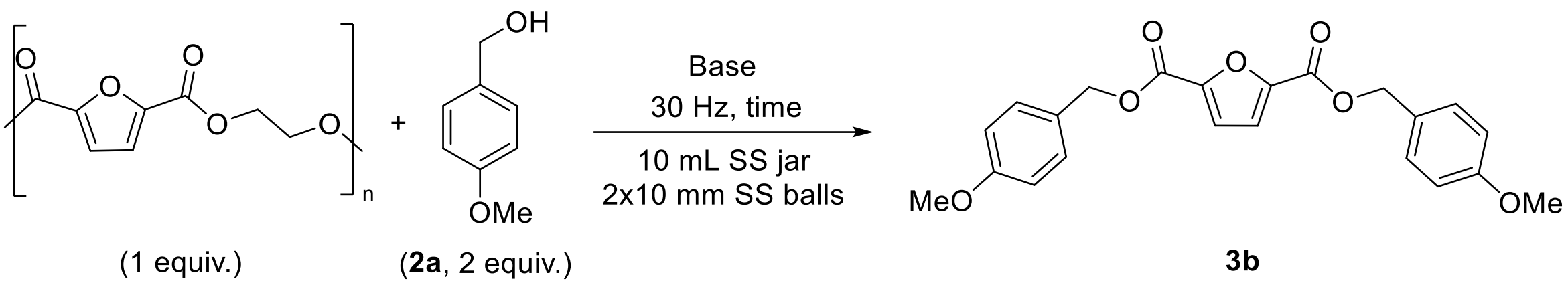

Entry	Base/catalyst	Time (min)	PEF conversion into product (%)[b]	Yield (%)[c]
1.	Na_2CO_3 (3 eq.)	90	0	0
2.	^{t}BuNH$_2$/NaCl (2-4 eq.)	90	0	0
3.	DBU:MSA salt (0.2-0.5 eq.)	60	0	0
4.	PPTS (0.5 eq.)	60	0	0
5.	NaOH (2.5 eq)	90	0	0
6.	MeONa (0.4 eq.)	60[d]	85	81
7.	MeONa (0.4 eq.)	60	99	>98
**8.**	**MeONa (0.3 eq.)**	**60**	**>98**	**98**
9.	MeONa (0.2 eq.)	90	>95	94
10.	None	90	0	0
11.	MeONa (0.3 eq.)	90[e]	>96	96

[a] Reaction conditions: PEF (182 mg, 1 mmol per repeating unit, 1 equiv.) and 4-methoxybenzyl alcohol (2 mmol, 2 equiv.) were ball milled in the presence of base/catalyst at 30 Hz. [b] Conversion was calculated by measuring remaining amount of polymer after mechanochemical upcycling reaction. [c] Product isolated yields have been calculated upon measuring and calculation the mmols of obtained product and dividing it by mmols of polymer repeating unit use for the reaction. [d] 25 Hz ball milling frequency. [e] Use of ZrO_2 jar and milling balls. DBU: 1,8-Diazabicyclo[5.4.0]undec-7-ene, MSA: Methylsulfonic acid, PPTS: Pyridinium *p*-toluensulfonate, SS: Stainless steel.

Table S2. Optimisation of mechanochemical amidation of PEF.[a]

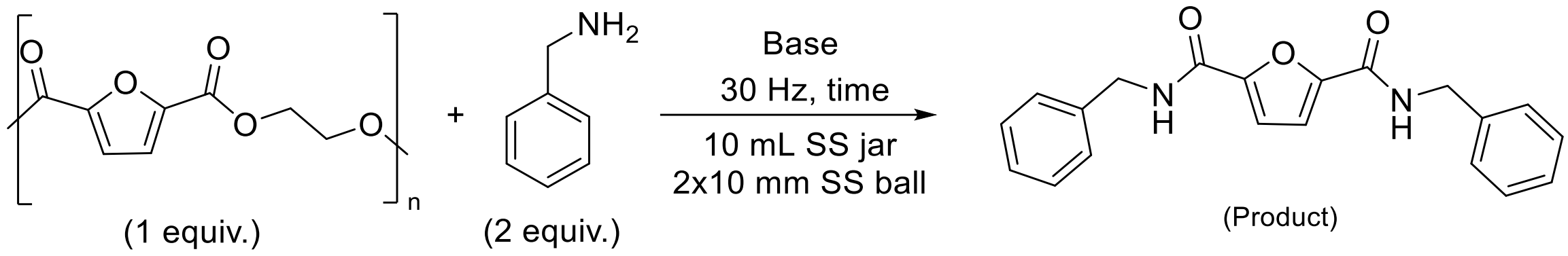

Entry	Base	Auxiliary	Time (min)	PEF conversion into product (%)	Yield (%)
1.	MeONa (0.2 eq.)	None	60	>90	93
2.	MeONa (0.3 eq.)	None	60	>98	99
3.	None	NaCl (4 equiv.)	90	<60 (mixture of products)	31 (major)
4.	$ZnBr_2$ (0.2 eq.)	NaCl (4 equiv.)	90	81%	80

[a] Reaction conditions: PEF (182 mg, 1 mmol per repeating unit, 1 equiv.) and benzyl amine (2 mmol, 2 equiv.) were ball milled in the presence of base at 30 Hz. SS: Stainless steel.

General procedure for the mechanochemical upcycling of polyesters

The small-scale polyester alcoholysis was carried out in a Retsch MM400 mixer mill at the frequency of 30 Hz using a 10 mL stainless steel grinding jar and two ball of 10 mm diameter (*m* = 4 g).

General procedure (GP1) for alcoholytic depolymerization

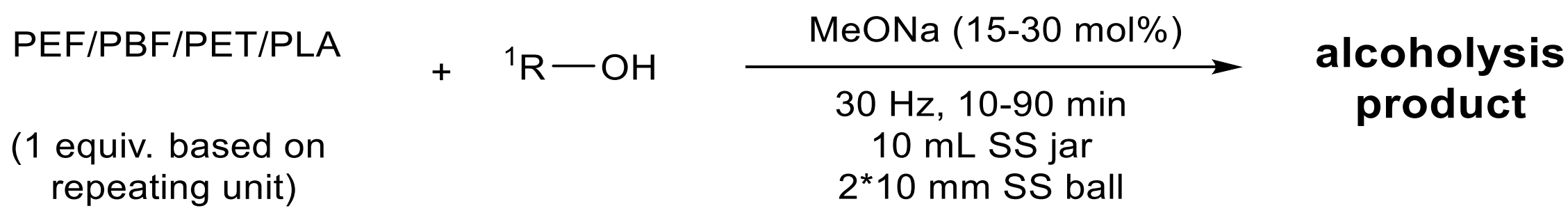

Scheme S1. Mechanochemical tranesterification of polyesters.

A mixture of PEF (182 mg, 1 equiv., 1 mmol based on repeating unit), MeONa (16.2 mg, 30 mol%), and the corresponding alcohol (2a-2d, 2-2.2 equiv.) were ball milled for 60-90 min at 30 Hz. After completion of the reaction, all PEF was converted into product, the reaction mixture was suspended in water and two types of workup procedures have been followed for solid and liquid products, respectively. In case of alcohols **2a** and **2b**, the reaction mixture was washed with brine and water to remove all MeONa and alcohol impurity, dried overnight at 60 °C, yielding over 90% of solid alcoholysis product. In case of alcohols **2c**-**2e** the products are liquid at ambient conditions. Therefore, the product was extracted using workup with ethyl acetate and aqueous $NaHCO_3$ followed by washing with water.

*Note: In case of DIAF synthesis from PEF, isoamyl alcohol (**2e**) was used in excess amount due to its low boiling point. Therefore, during workup, the organic phase was washed three times with aqueous $NaHCO_3$ to remove all alcohol impurity and obtain the highly pure final product.*

A similar reaction procedure was followed for the mechanochemical alcoholysis of PBF (210 mg, 1 equiv., 1 mmol based on repeating unit) and PET (192 mg, 1 mmol, 1 equiv., based on repeating unit). In case of PET, instead of two 10 mm SS balls one 15 mm SS ball was used along with NaCl (2 equiv.) as inert grinding media.

For the mechanochemical alcoholysis of PLA, a mixture of PLA (144 mg, 1 equiv., 2 mmol based on repeating using), MeONa (16.2 mg, 15 mol%), and 4-methoxybenzyl alcohol (249 μL, 1 equiv.) were ball milled for 10 min at 30 Hz. After completion of the reaction, almost all the PLA was converted into product and the mixture was extracted in ethyl acetate during workup with aqueous $NaHCO_3$ followed by washing with water, yielding over 90% of highly pure product.

General procedure (GP2) for aminolytic depolymerization

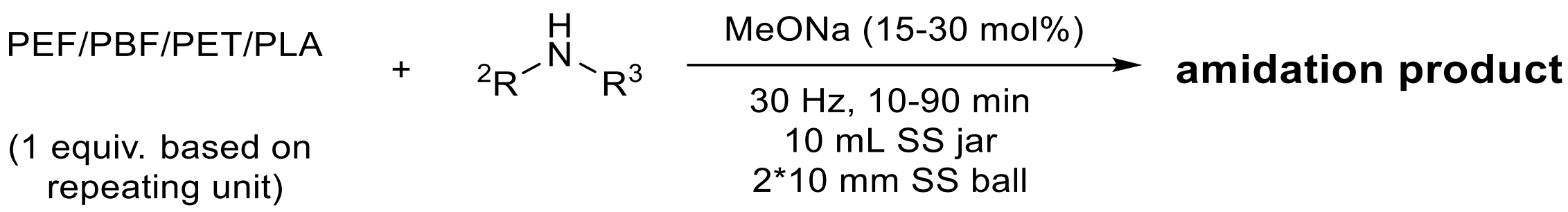

Scheme S2. Mechanochemical amidation of polyesters.

A mixture of PEF (182 mg, 1 equiv., 1 mmol based on repeating unit), MeONa (16.2 mg, 30 mol%), and the corresponding amine (**1a**-**1b**, 2-2.2 equiv.) were ball milled for 60 min at 30 Hz. After completion of the reaction, all PEF was converted into the corresponding amidation product, the reaction mixture was washed with brine and water to remove all MeONa and amine impurity, dried overnight at 60 °C, yielding over 90% of solid amidation product.

A similar reaction procedure was followed for the mechanochemical amidation of PBF (194 mg, 1 equiv., 1 mmol based on repeating unit) and PET (1 equiv
mg, 1 mmol, 1 equiv., based on repeating unit). In case of PET, instead of two 10 mm SS balls one 15 mm SS ball was used along with NaCl (2 equiv.) as inert grinding media.

For the mechanochemical amidation of PLA, a mixture of PLA (144 mg, 1 equiv., 2 mmol based on repeating using), MeONa (16.2 mg, 15 mol%), and morpholine (1 equiv.) were ball milled for 10 min at 30 Hz. After completion of the reaction, almost all PLA was converted into product and extracted in ethyl acetate during acidic workup, yielding over 90% of highly pure product.

Note: For all experiment mmols of polymer were calculated with respect to the mmols of the repeating unit of the corresponding polymer. To calculate the mmols, the used mass of polymer was divided by the molecular weight of its repeating unit. The yield of the final product was calculated by dividing the mmols of obtained product by the mmols of polymer used.

Products obtained from PEF

Synthesis of furan-2,5-diylbis(morpholinomethanone) (3a)

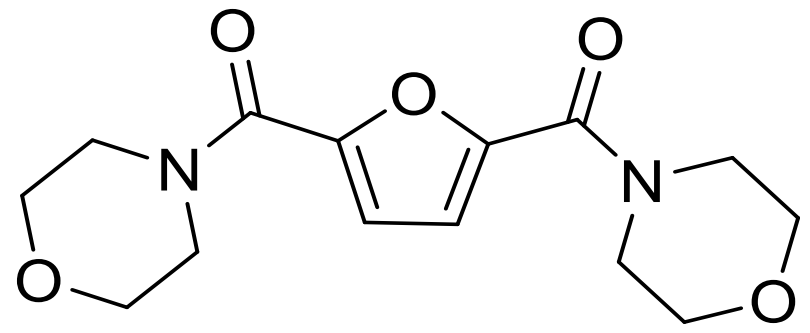

Following the general procedure GP2, using polyethylene furanoate (182 mg, 1 mmol) and morpholine (**1a**, 2.1 equiv., 2.1 mmol) afforded the title compound **3a** (285 mg, 97%) in 60 min as a white solid after workup with acidic workup (using 1M HCl solution) followed by extraction in ethyl acetate. Crystals suitable for single-crystal X-ray diffraction analysis were obtained by recrystallisation from ethanol/acetone (1:1) at 7 °C.

^{1}H NMR (400 MHz, DMSO-d_6) δ = 7.10 (s, 2H), 3.64-3.63 (m, 16H).
^{13}C NMR (101 MHz, DMSO-d_6) δ = 157.7, 147.2, 115.9, 66.
HRMS (ESI-TOF): *m/z* $[M+H]^+$ calcd for $[C_{14}H_{18}N_2O_5+H]^+$: 295.1289 found; 295.1292.

Synthesis of N^2,N^5-dibenzylfuran-2,5-dicarboxamide (3b)[3]

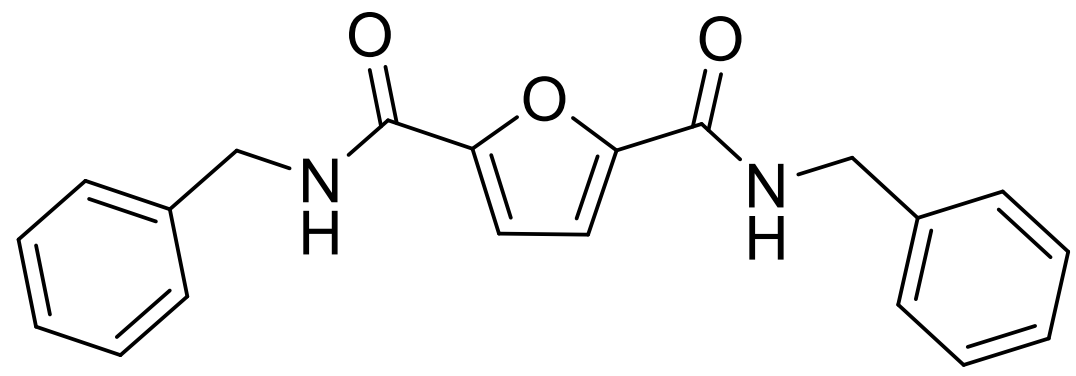

Following the general procedure GP2, using polyethylene furanoate (182 mg, 1 mmol) and benzyl amine (**1b**, 2 equiv., 2 mmol) afforded the title compound **3b** (330 mg, 99%) in 60 minutes as a pure white solid after thoroughly washing the reaction mixture with water. Crystals suitable for single-crystal X-ray diffraction analysis were obtained by recrystallisation from ethanol/acetone (2:1) at 7 °C.

^{1}H NMR (400 MHz, DMSO-d_6) δ = 9.03-9.00 (t, 2H), 7.34-7.25 (m, 10H), 7.20 (s, 2H), 4.51-4.49 (d, 4H).
^{13}C NMR (101 MHz, DMSO-d_6) δ = 157.7, 148.5, 139.4, 128.9, 127.7, 127.4, 115,2, 52.3.
HRMS (ESI-TOF): *m/z* $[M+Na]^+$ calcd for $[C_{20}H_{18}N_2O_3+Na]^+$: 357.1209 found; 357.1215.
IR (ATR, neat, cm^{-1}): 3306, 3242, 3124, 3083, 3031, 2244, 1664, 1647, 1593, 1594, 1575, 1494, 1455, 1434, 1355, 1273, 1223, 1165, 1043, 1021, 998, 934, 833, 823, 723, 690, 612, 579, 512, 495, 447.

Synthesis of bis(4-methoxybenzyl) furan-2,5-dicarboxylate (3c)

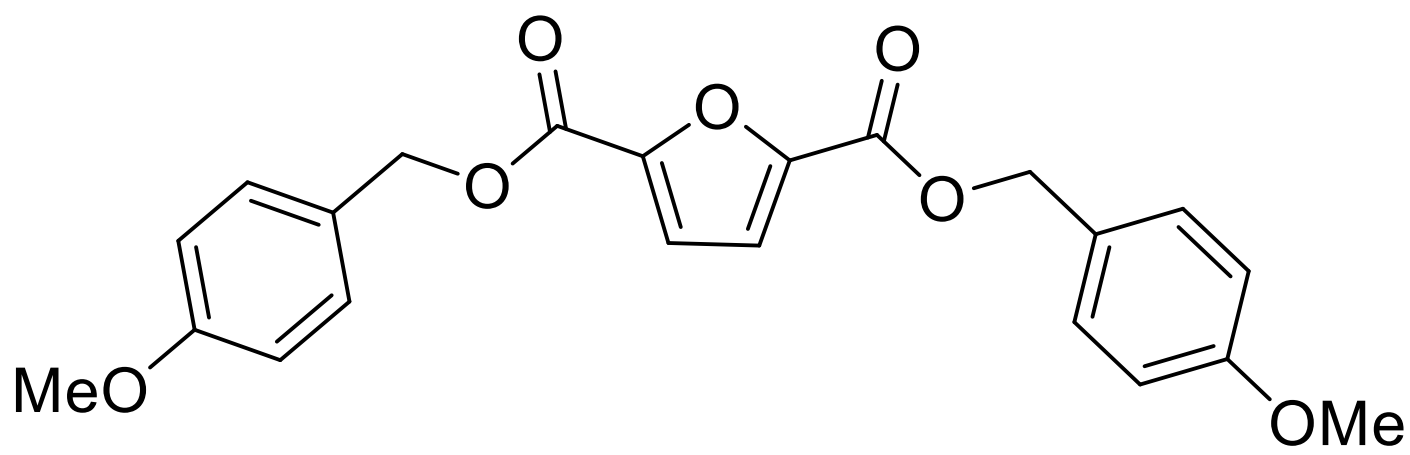

Following the general procedure GP1, using polyethylene furanoate (182 mg, 1 mmol) and 4-methoxybenzyl alcohol (**2a**, 2 equiv., 2 mmol) afforded the title compound **3c** (388 mg, 98%) in 60 minutes as a white solid after thoroughly washing the reaction mixture with water. Crystals suitable for single-crystal X-ray diffraction analysis were obtained by recrystallisation from ethanol/acetone (2:1) at 7 °C.

^{1}H NMR (300 MHz, $CDCl_3$) δ = 7.39-7.36 (dd, 4H), 7.17 (s, 2H), 6.91-6.88 (dd, 4H), 5.29 (s, 4H), 3.81 (s, 6H).
^{13}C NMR (75 MHz, $CDCl_3$) δ = 159.8, 157.9, 146.8, 130.5, 127.3, 118.5, 114.0, 67.0, 55.3.
HRMS (ESI-TOF): *m/z* $[M+Na]^+$ calcd for $[C_{22}H_{20}O_7+Na]^+$: 419.1101 found; 419.1111.
IR (ATR, neat, cm^{-1}): 3381, 3148, 3010, 2987, 2961, 2944, 2934, 2853, 2836, 2157, 1692, 1613, 1584, 1535, 1517, 1454, 1389, 1313, 1250, 1232, 1178, 1156, 1117, 1027, 963, 947, 915, 870, 818, 808, 754, 614, 586, 559, 526, 442.

Synthesis of bis(4-nitrobenzyl) furan-2,5-dicarboxylate (3d)

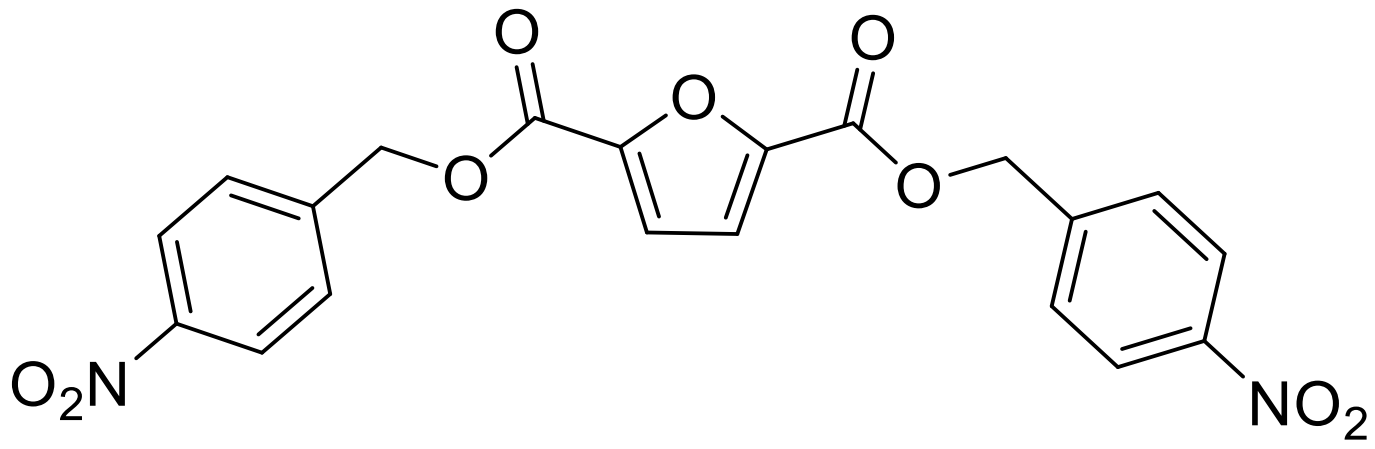

Following the general procedure GP1, using polyethylene furanoate (182 mg, 1 mmol) and 4-nitrobenzyl alcohol (**2b**, 2 equiv., 2 mmol) afforded the title compound **3d** (401 mg, 94%) in 60 minutes as yellow solid after thoroughly washing with water.

^{1}H NMR (400 MHz, DMSO-d_6) δ = 8.26-8.25 (m, 4H), 7.74-7.57 (m, 6H), 5.52 (s, 4H), 3.81 (s, 4H).
^{13}C NMR (101 MHz, DMSO-d_6) δ = 157.0, 147.2, 145.9, 143.1, 128.8, 123.6, 119.7, 65.4.
HRMS (ESI-TOF): *m/z* $[M+Na]^+$ calcd for $[C_{20}H_{14}N_2O_9 +Na]^+$: 449.0591 found; 449.0585.

Synthesis of bis(2-ethylhexyl) furan-2,5-dicarboxylate/diethylhexyl furanoate (DEHF) (3e)[4]

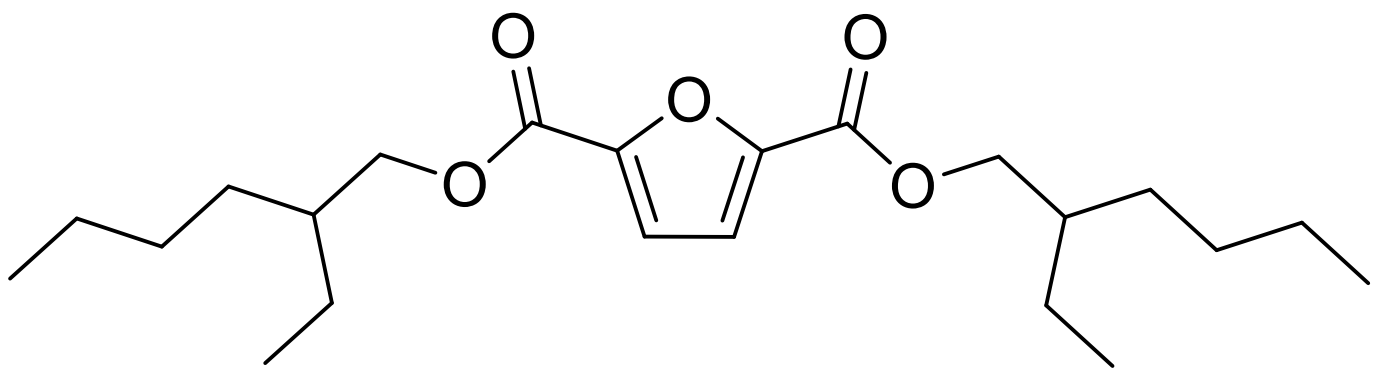

Following the general procedure GP1, using polyethylene furanoate (182 mg, 1 mmol) and 2-ethylhexanol (**2c**, 2.2 equiv., 2.2 mmol) afforded the title compound **3e** (346 mg, 91%) in 90 minutes as a colourless liquid after basic workup (aqueous $NaHCO_3$) followed by extraction in diethyl ether.

^{1}H NMR (400 MHz, $CDCl_3$) δ = 7.18 (s, 2H), 4.26-4.23 (dd, 4H), 2.37 (m, 2H), 1.45-1.30 (m, 16H), 0.95-0.88 (m, 12H).
^{13}C NMR (101 MHz, $CDCl_3$) δ = 158.4, 147.1, 118.2, 68.0, 38.9, 30.5, 29.0, 23.9, 23.0, 14.1, 11.15.
HRMS (ESI-TOF): *m/z* $[M+Na]^+$ calcd for $[C_{22}H_{36}O_5+Na]^+$: 403.2455 found; 403.2451.

Synthesis of bis(2-methoxyethyl) furan-2,5-dicarboxylate (3f)

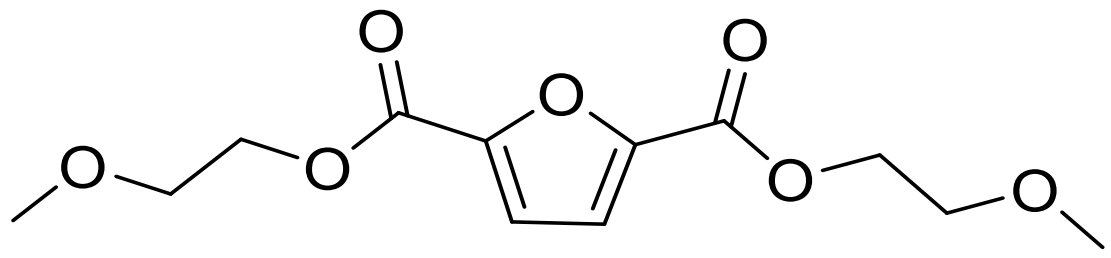

Following the general procedure GP1, using polyethylene furanoate (182 mg, 1 mmol) and 2-methoxyethanol (**2d**, 2.1 equiv., 2.1 mmol) afforded the title compound **3f** (242 mg, 89%) in 90 minutes as a colourless liquid after basic workup (aqueous $NaHCO_3$) followed by extraction in diethyl ether.

^{1}H NMR (400 MHz, $CDCl_3$) δ = 7.21 (s, 2H), 4.46-4.43 (dd, 4H), 3.69-3.66 (dd, 4H), 3.38 (s, 6H).
^{13}C NMR (101 MHz, $CDCl_3$) δ = 157.9, 146.6, 118.6, 70.1, 64.4, 59.0, 52.3.
HRMS (ESI-TOF): *m/z* $[M+Na]^+$ calcd for $[C_{12}H_{16}O_7+Na]^+$: 295.0788 found; 295.0787.

Synthesis of diisopentyl furan-2,5-dicarboxylate/diisoamyl furanoate (DIAF) (3g)

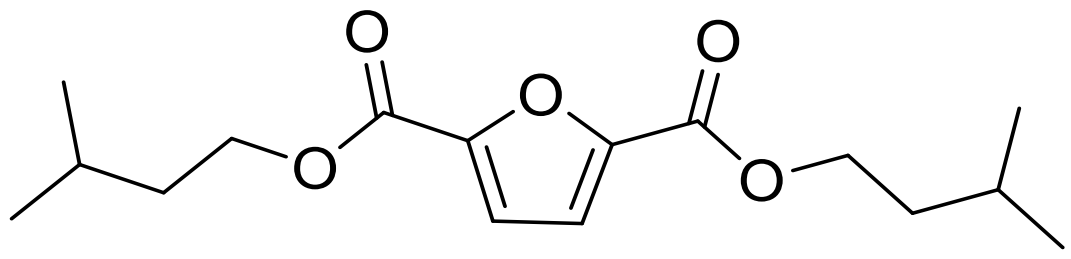

Following the general procedure GP1, using polyethylene furanoate (182 mg, 1 mmol) and 3-methylbutanol/isoamyl alcohol (**2e**, 4 equiv. 4 mmol) afforded the title compound **3g** (275 mg, 93%) in 60 minutes as a colourless liquid after basic workup (aqueous $NaHCO_3$) followed by extraction in diethyl ether.
Note: Excess amount of the alcohol was taken because of lower boiling point of isoamyl alcohol, to obtain full conversion of PEF into DIAF. To remove this excess amount, washed it with saturated solution of aqueous $NaHCO_3$ was done 3-4 times.

^{1}H NMR (400 MHz, $CDCl_3$) δ = 7.14 (s, 2H), 4.33-4.29 (dd, 4H), 1.73-1.56 (m, 6H), 0.92-0.90 (d, 12H).
^{13}C NMR (101 MHz, $CDCl_3$) δ = 158.1, 146.9, 118.2, 64.2, 37.2, 25.1, 22.4.
HRMS (ESI-TOF): *m/z* $[M+Na]^+$ calcd for $[C_{16}H_{24}O_5+Na]^+$: 319.1516 found; 319.1512.

Synthesis of di(pent-4-en-1-yl) furan-2,5-dicarboxylate (3h)

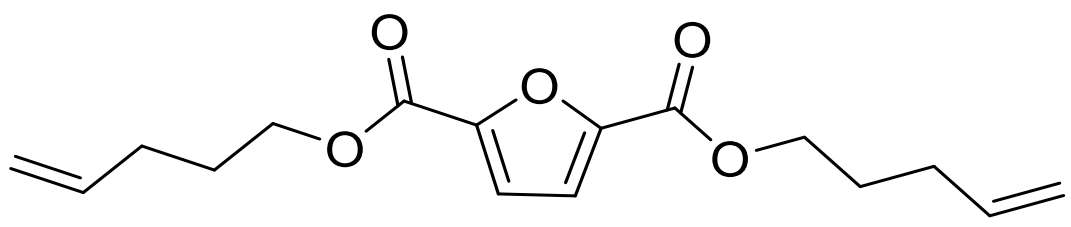

Following the general procedure GP1, using polyethylene furanoate (182 mg, 1 mmol) and 4-pentene-1-ol (**2g**, 4 equiv. 4 mmol) afforded the title compound **3h** (278 mg, 95%) in 60 minutes as a colourless liquid after basic workup same as **3g**.

^{1}H NMR (300 MHz, $CDCl_3$) δ = 7.13 (s, 2H), 5.79-5.67 (m, 2H), 5.0-4.89 (m, 4H), 4.28-4.23 (t, 2H), 3.57-3.53 (t, 2H), 2.12-2.02 (m, 4H), 1.82-1.73 (m, 2H), 1.59-1.54 (m, 2H).
^{13}C NMR (75 MHz, $CDCl_3$) δ = 158, 146.7, 138.2, 137.1, 118.3, 115.4, 114.6, 64.8, 61.95, 31.6, 29.9, 29.8, 27.6.
HRMS (ESI-TOF): *m/z* $[M+Na]^+$ calcd for $[C_{16}H_{20}O_5+Na]^+$: 315.1203 found; 315.1200.

Synthesis of N^2,N^5-diphenylfuran-2,5-dicarboxamide (3l)

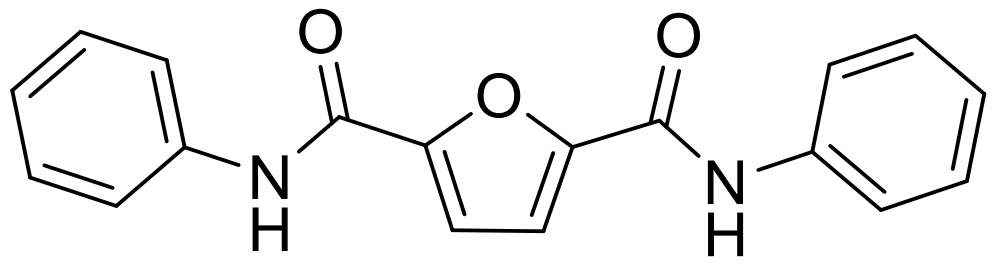

Around 40% yield of product using LiHMDS as a base

Following the general procedure GP2, using polyethylene furanoate (182 mg, 1 mmol) aniline (**1d**, 2 equiv. 2 mmol), and lithium bis(trimethylsilyl)amide (LiHMDS) (2 equiv.) instead of MeONa afforded the title compound **3l** (122 mg, 40%) in 60 minutes as a white solid after washing the reaction mixture with water and hexane, respectively, to remove base, ethylene glycol and hexamethyldisilazane (HMDS) byproducts. Crystals suitable for single-crystal X-ray diffraction analysis were obtained by recrystallisation from ethanol/acetone (1:1) at 7 °C.

^{1}H NMR (400 MHz, $CDCl_3$) δ = 8.69 (s, 2H), 7.61-7.59 (m,4H), 7.29-7.26 (t, 4H), 7.22 (s, 2H), 7.14-7.10 (t, 2H).
^{13}C NMR (101 MHz, $CDCl_3$) δ = 155.9, 148.6, 136.9, 129.2, 125.3, 120.8, 116.7.

Products obtained from PBF

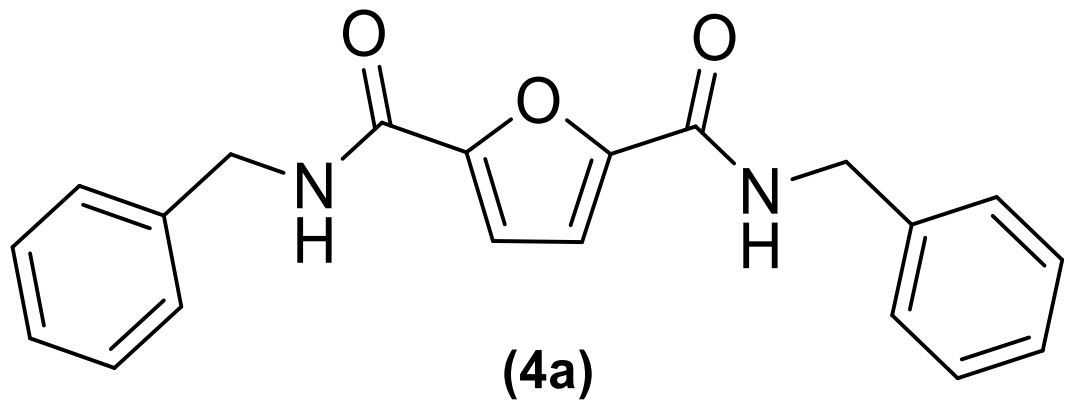

(4a)

Following the general procedure GP2, using polybutylene furanoate (210 mg, 1 mmol repeating unit) and benzyl amine (**1b**, 2 equiv., 2 mmol) afforded the title compound **3b** (314 mg, 94%) in 60 minutes as a pure white solid after thoroughly washing the reaction mixture with water.

^{1}H NMR (400 MHz, DMSO-d$_6$) δ = 8.42-7.51 (t, 2H), 7.70-7.62 (m, 10H), 7.51 (s, 2H), 4.87-4.84 (d, 4H).
^{13}C NMR (101 MHz, DMSO-d$_6$) δ = 158.0, 148.0, 137.4, 128.5, 127.7, 127.5, 115.5, 43.1.

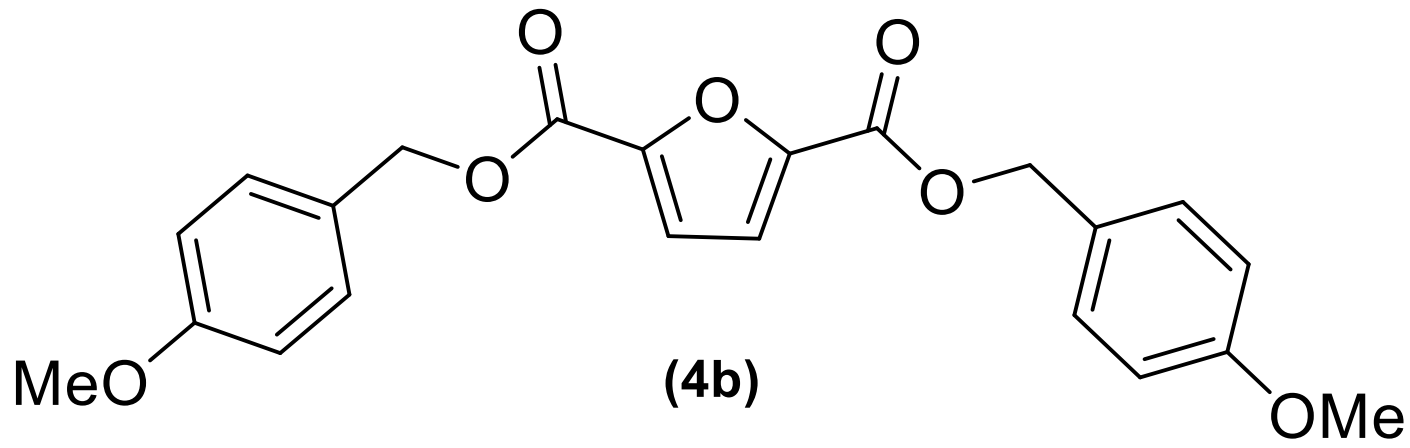

(4b)

Following the general procedure GP1, using polybutylene furanoate (210 mg, 1 mmol repeating unit) and 4-methoxybenzyl alcohol (**2a**, 2 equiv., 2 mmol) afforded the title compound **3c** (379 mg, 98%) in 60 minutes as a white solid after thoroughly washing the reaction mixture with water.

^{1}H NMR (300 MHz, CDCl$_3$) δ = 7.41 (s, 2H), 7.40-7.38 (m, 4H), 6.96-6.93 (dd, 4H), 5.27 (s, 4H), 3.81 (s, 6H).
^{13}C NMR (75 MHz, CDCl$_3$) δ = 159.4, 157.3, 146.1, 130.5, 127.2, 119.2, 113.9, 66.5, 55.1.

Products obtained from PET

Synthesis of 1,4-phenylenebis(morpholinomethanone) (5a)[5]

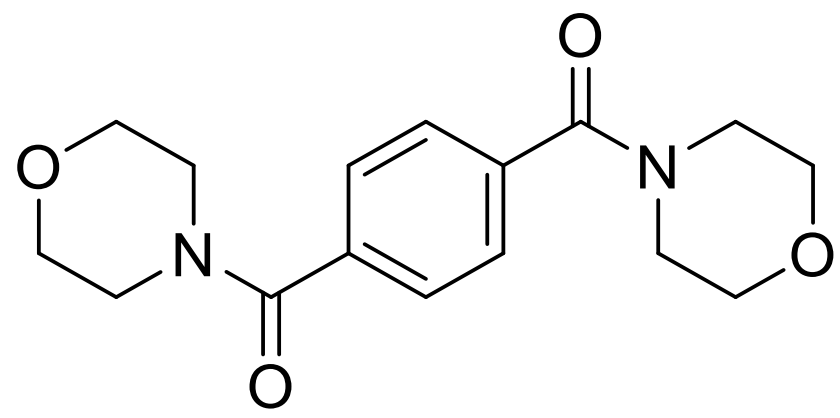

Following the general procedure GP2, using polyethylene terephthalate (PET) (192 mg, 1 mmol) and morpholine (**1a**, 2 equiv., 2 mmol) afforded the title compound **5a** (285 mg, 94%) in 60 minutes as a pure white solid after thoroughly washing the reaction mixture with water.

^{1}H NMR (400 MHz, $CDCl_3$) δ = 7.30-7.29 (m, 4H), 3.59-3.26 (m, 16H).
^{13}C NMR (101 MHz, $CDCl_3$) δ = 168.9, 136.4, 127.0, 66.4.
HRMS (ESI-TOF): *m/z* $[M+Na]^+$ calcd for $[C_{16}H_{20}N_2O_4+H]^+$: 305.1496 found; 305.1496.

Synthesis of bis(4-methoxybenzyl) terephthalate (5b)

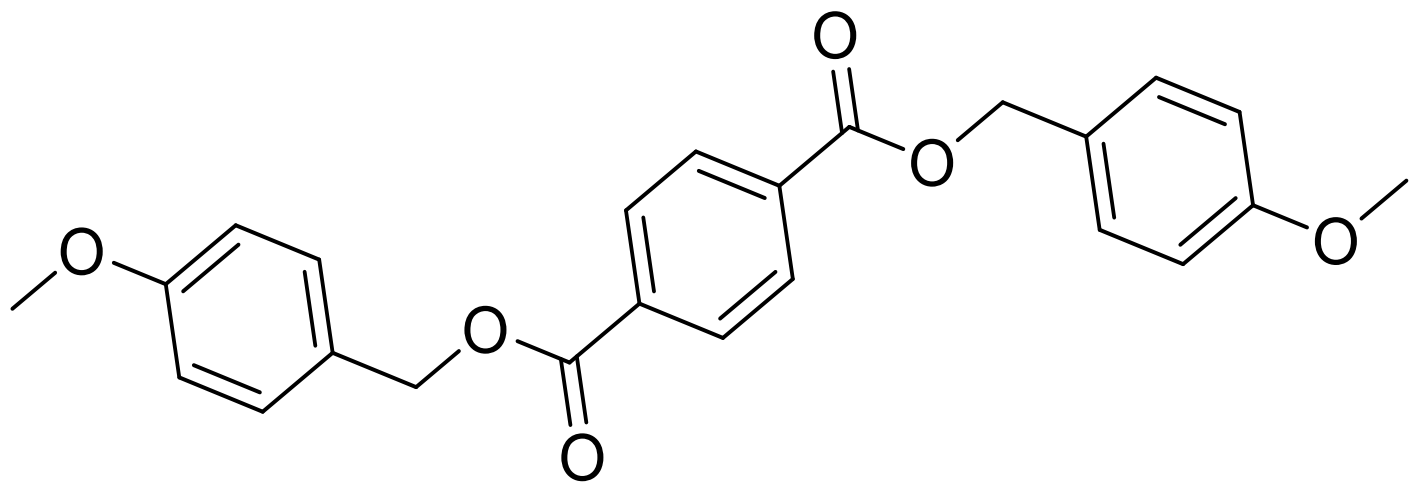

Following the general procedure GP1, using polyethylene terephthalate (PET) (192 mg, 1 mmol), 4-methoxybenzyl alcohol (**2a**, 2.1 equiv., 2.1 mmol), and NaCl (2 equiv.) afforded the title compound **5b** (387 mg, 96%) in 60 minutes as a pure white solid after thoroughly washing the reaction mixture with water. Crystals suitable for single-crystal X-ray diffraction analysis were obtained by recrystallisation from ethanol/acetone (1:1) at 7 °C.

^{1}H NMR (300 MHz, $CDCl_3$) δ = 8.09 (dd, 4H), 7.41-7.38 (dd, 4H), 6.93-6.91 (dd, 4H), 5.31 (s, 4H), 3.82 (s, 6H).
^{13}C NMR (75 MHz, $CDCl_3$) δ = 165.8, 159.9, 134.1, 130.3, 129.7, 127.8, 114.1, 67.12, 55.42.
HRMS (ESI-TOF): *m/z* $[M+Na]^+$ calcd for $[C_{24}H_{22}O_6+Na]^+$: 429.1308 found; 429.1317.

Synthesis of bis(2-methoxyethyl) terephthalate (5c)[6]

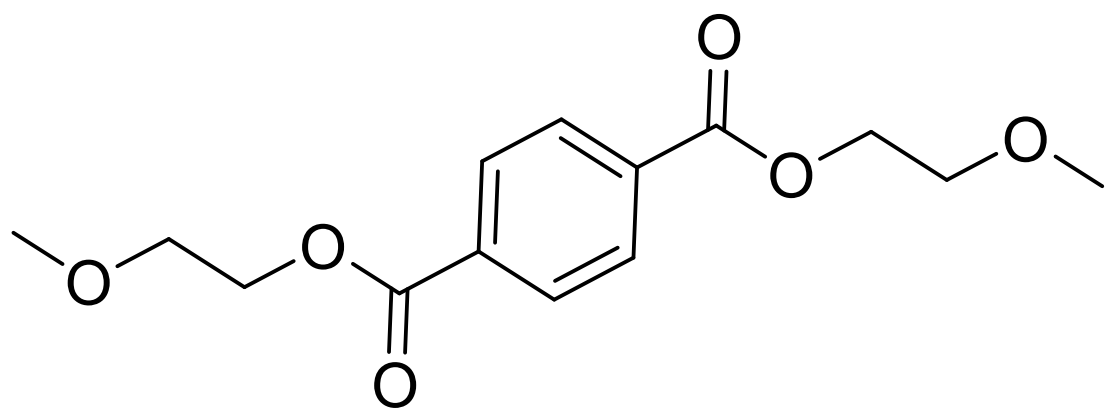

Following the general procedure GP1, using polyethylene terephthalate (PET) (192 mg, 1 mmol) and 2-methoxyethanol (**2d**, 2.5 equiv., 2.5 mmol) afforded the title major compound **5c** (240 mg, mixture of products, >80% PET conversion into alcoholysis products) in 60 minutes as colourless liquid after basic workup followed by extraction in diethyl ether.

^{1}H NMR (400 MHz, $CDCl_3$) δ = 7.25-7.21 (dd, 4H), 3.67-3.64 (t, 4H), 2.92-2.89 (t, 4H), 5.31 (s, 4H), 2.59 (s, 6H).

^{13}C NMR (101 MHz, $CDCl_3$) δ = 165.5, 133.7, 129.7, 129.4, 70.2, 64.2, 58.8.

Synthesis of bis(2-ethylhexyl) terephthalate/diethylhexyl terephthalate (DEHP) (5d) [7]

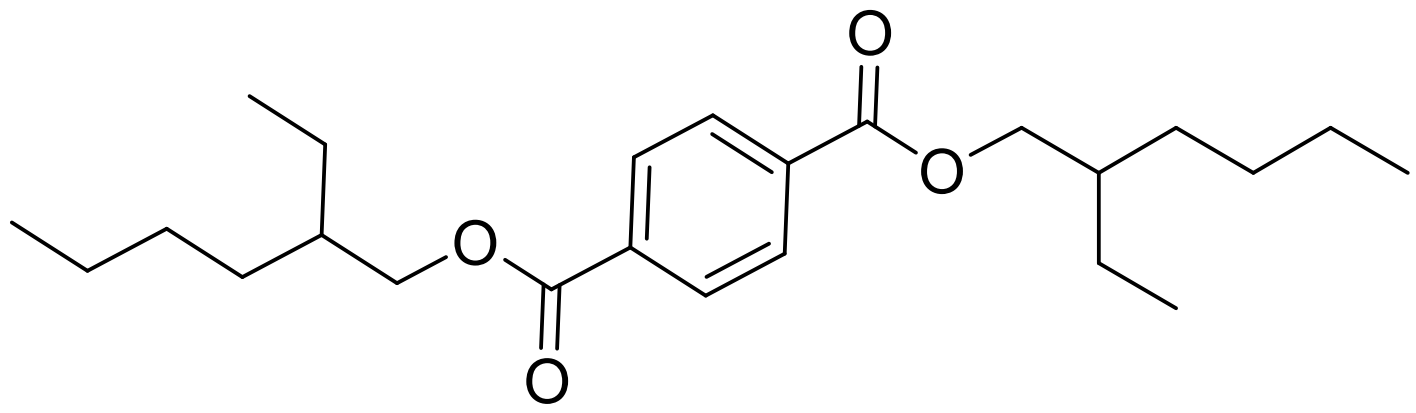

Following the general procedure GP1, using polyethylene terephthalate (192 mg, 1 mmol) and 2-ethyl hexanol (**2c**, 2 equiv., 2 mmol) afforded 33% conversion of PET into the title compound **5d** within 90 minutes as a colourless liquid after basic workup (aqueous $NaHCO_3$) followed by extraction in diethyl ether.

^{1}H NMR (300 MHz, $CDCl_3$) δ = 8.11-7.08 (m, 4H), 4.27-4.24 (dd, 4H), 1.74-1.70 (t, 4H), 1.5-0.86 (m, 38H).
HRMS (ESI-TOF): *m/z* $[M+Na]^+$ calcd for $[C_{24}H_{38}O_4+Na]^+$: 413.2662 found; 413.2672.

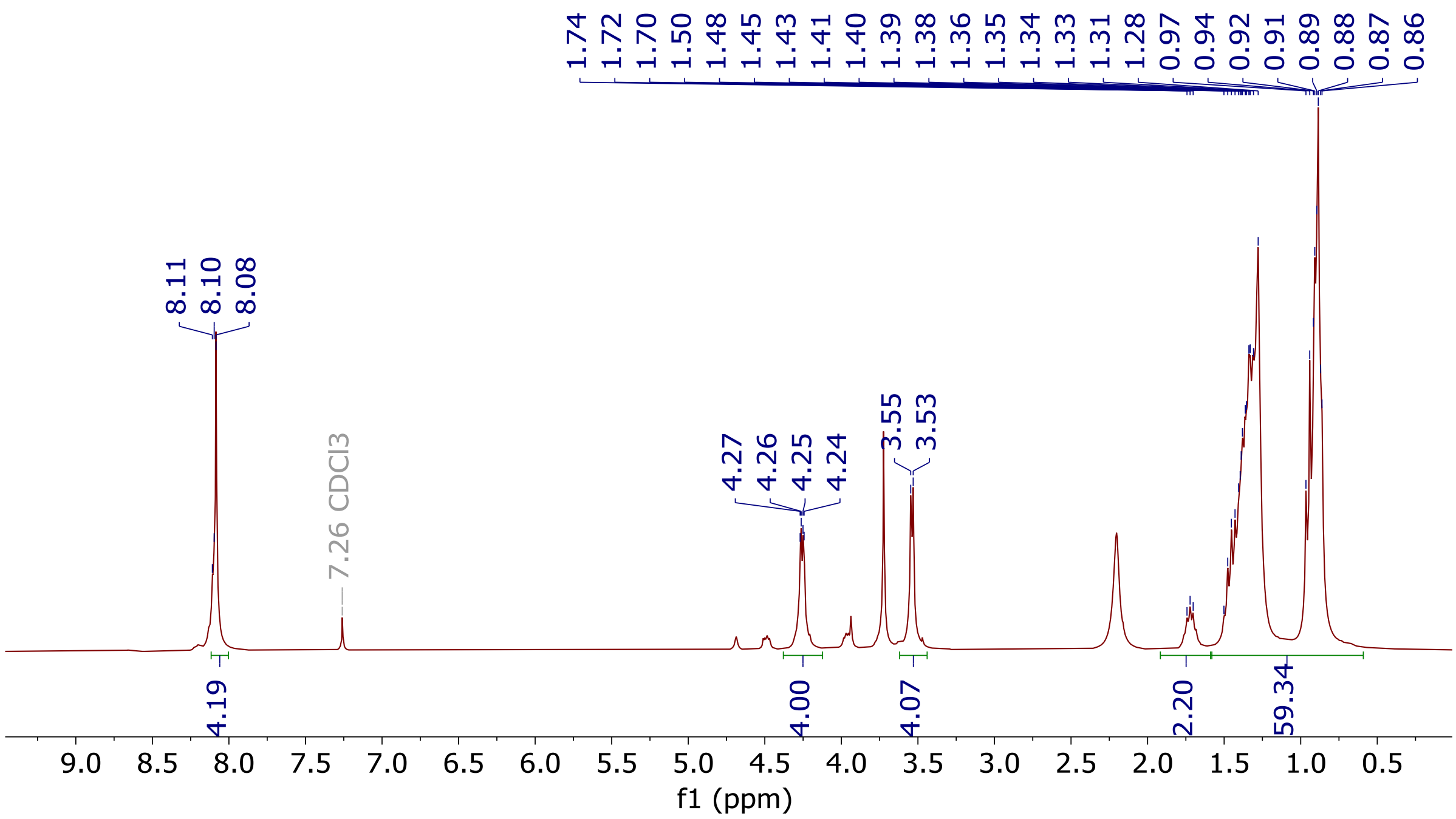

Figure S18. ^{1}H NMR spectrum ($CDCl_3$, 300 MHz, 297 K) of reaction mixture (**5d**), showing 33% conversion of PET into DEHP.

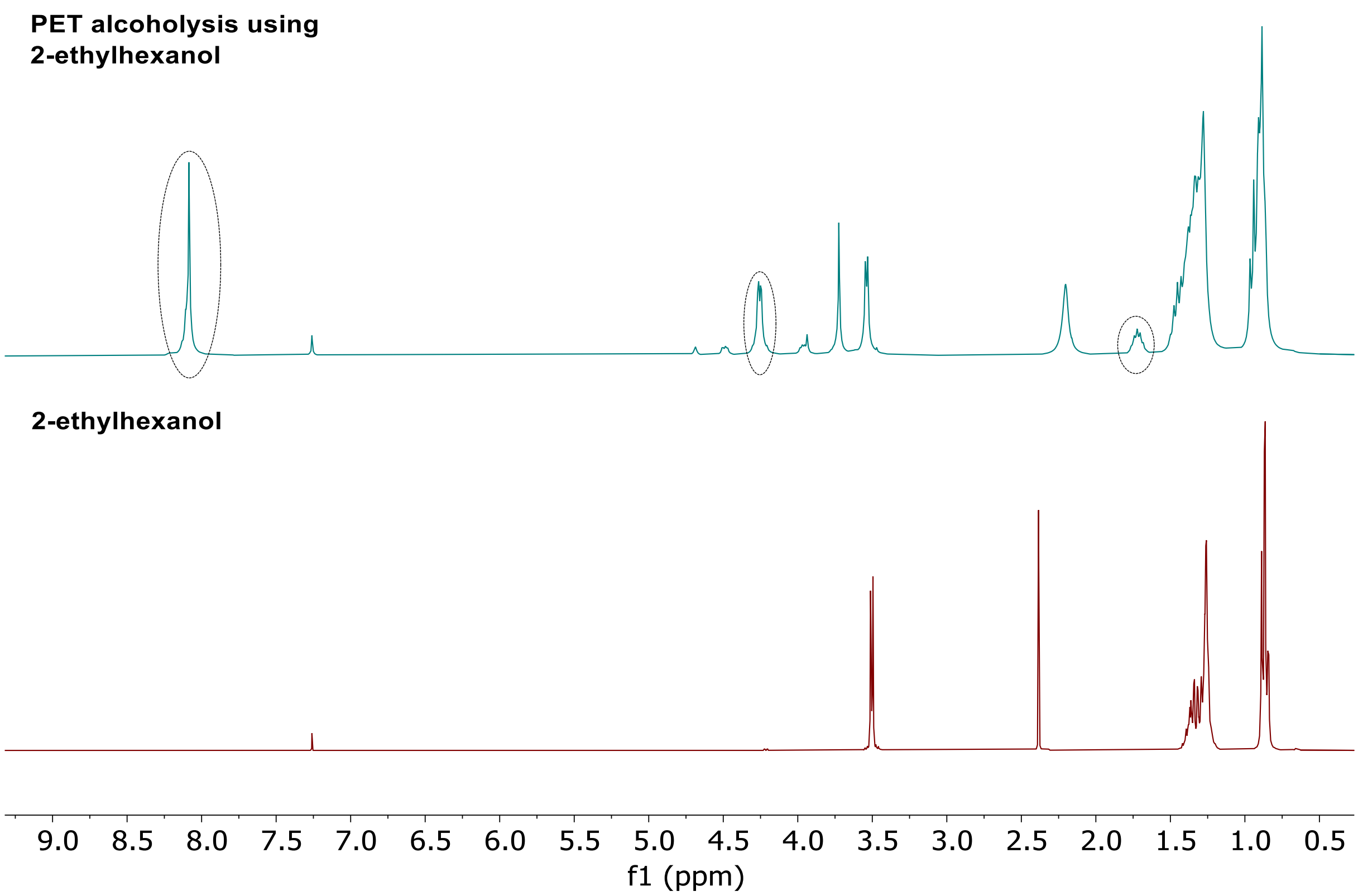

Figure S19. ^{1}H NMR spectra [$CDCl_3$:TFA-d_1(1:1), 300 MHz, 297 K], showing formation of DEHP (dotted circle) and remaining starting material, 2-ethyl hexanol.

Product obtained from PLA

Synthesis of 2-hydroxy-1-morpholinopropan-1-one (6a)[8]

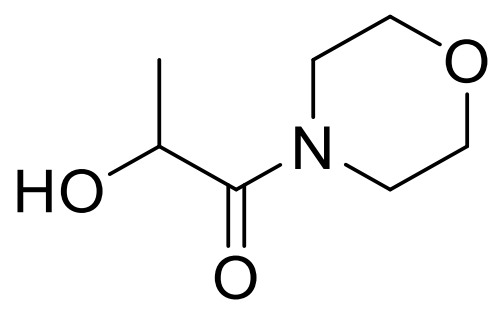

Following the general procedure GP2, using PLA (144 mg, 2 mmol of repeating unit, 1 equiv.) and morpholine (1 equiv., 2 mmol) afforded the title compound **6a** (75% NMR yield) in 30 minutes as colourless liquid after extraction using diethyl ether and water.

^{1}H NMR (400 MHz, $CDCl_3$) δ = 4.46-4.41 (q, 1H), 3.78-3.73 (m, 9H), 1.32-1.30 (d, 3H).
^{13}C NMR (75 MHz, $CDCl_3$) δ = 173.9, 66.9, 64.1, 45.5, 21.4.
HRMS (ESI-TOF): *m/z* $[M+Na]^+$ calcd for $[C_7H_{13}NO_3+H]^+$: 160.0968 found; 160.0970.

Synthesis of 2-hydroxy-1-morpholinopropan-1-one (6b)

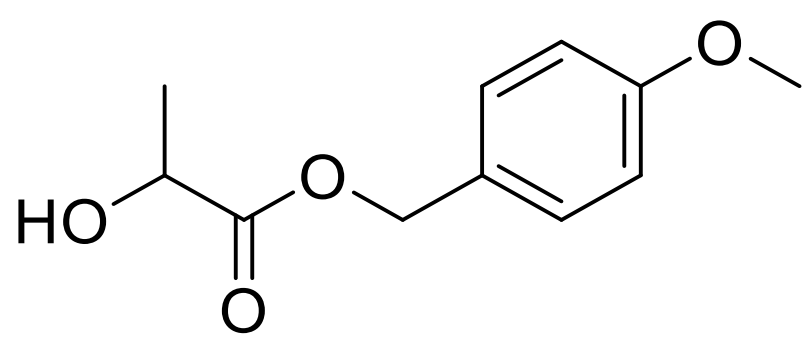

Following the general procedure GP1, using PLA (144 mg, 2 mmol of repeating unit, 1 equiv.) and 4-methoxybenzyl alcohol (1 equiv., 2 mmol) afforded the title compound **6b** (79% NMR yield) in 30 minutes as colourless liquid after extraction using diethyl ether and water.

^{1}H NMR (300 MHz, $CDCl_3$) δ = 7.31-7.30 (d, 2H), 6.91-6.89 (d, 2H), 5.14 (s, 2H), 4.32-4.29 (d, 1H), 3.81 (s, 3H), 2.67 (s, 1H), 1.42-1.39 (d, 3H).
^{13}C NMR (75 MHz, $CDCl_3$) δ = 175.7, 159.9, 130.3, 129.5, 114.1, 114.0, 113.9, 67.3, 66.9, 55.4, 20.4.
HRMS (ESI-TOF): *m/z* $[M+Na]^+$ calcd for $[C_{11}H_{14}O_4+Na]^+$: 233.0784 found; 233.0789.

Synthesis of pent-4-en-1-yl 2-hydroxypropanoate (6c)

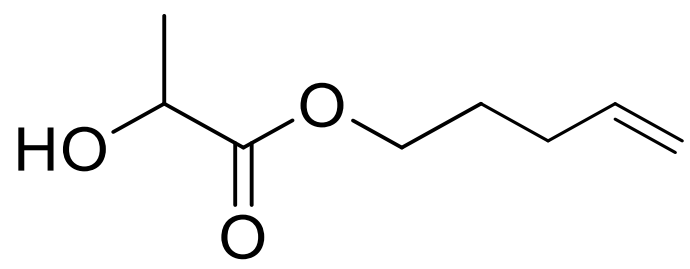

Following the general procedure GP1, using PLA (144 mg, 2 mmol of repeating unit, 1 equiv.) and excess amount of 4-pentene-1-ol (2 equiv., 4 mmol) afforded the title compound **6c** (96% NMR yield) in 30 minutes as colourless liquid after extraction using diethyl ether and aq. $NaHCO_3$.

^{1}H NMR (300 MHz, $CDCl_3$) δ = 5.81-5.75 (m, 1H), 5.03-4.92 (m, 2H), 4.25-4.18 (q, 1H), 4.17-4.13 (m, 1H), 3.64-3.59 (t, 1H), 3.00 (s, 1H), 2.12-2.06 (m, 2H), 1.76-1.63 (m, 2H), 1.40-1.37 (d, 3H).
^{13}C NMR (75 MHz, $CDCl_3$) δ = 186.1, 148.6, 147.5, 125.9, 125.2, 77.1, 75.3, 72.6, 42.1, 40.4, 40.3, 38.1, 30.8.

Mechanochemical upcycling of post-consumer plastics

PostPET

1 g of food packaging transparent PET material was ball milled with 2.17 mL of morpholine in the presence of 30 mol% MeONa using a 25 mL SS jar and a 15 mm SS ball at 30 Hz for 60 minutes. Complete conversion of the packaging material into a white solid product was observed.

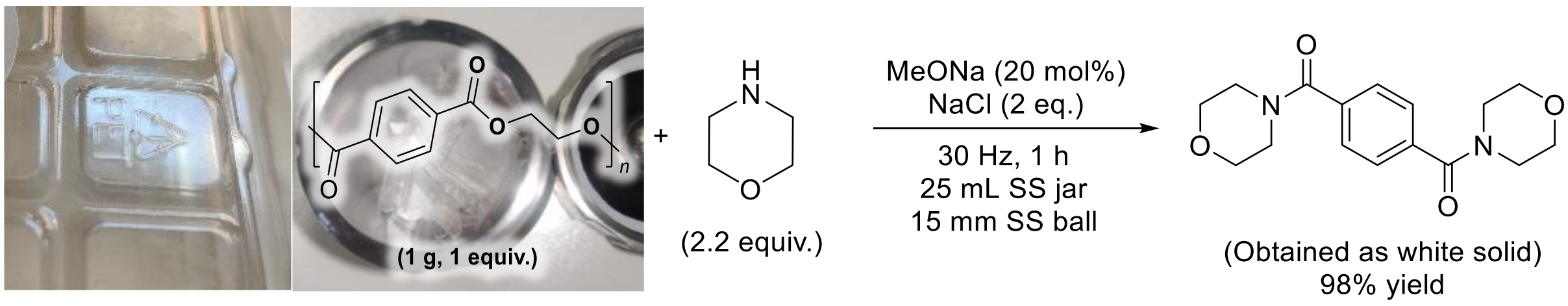

Scheme S3. Mechanochemical upcycling of post postPET material.

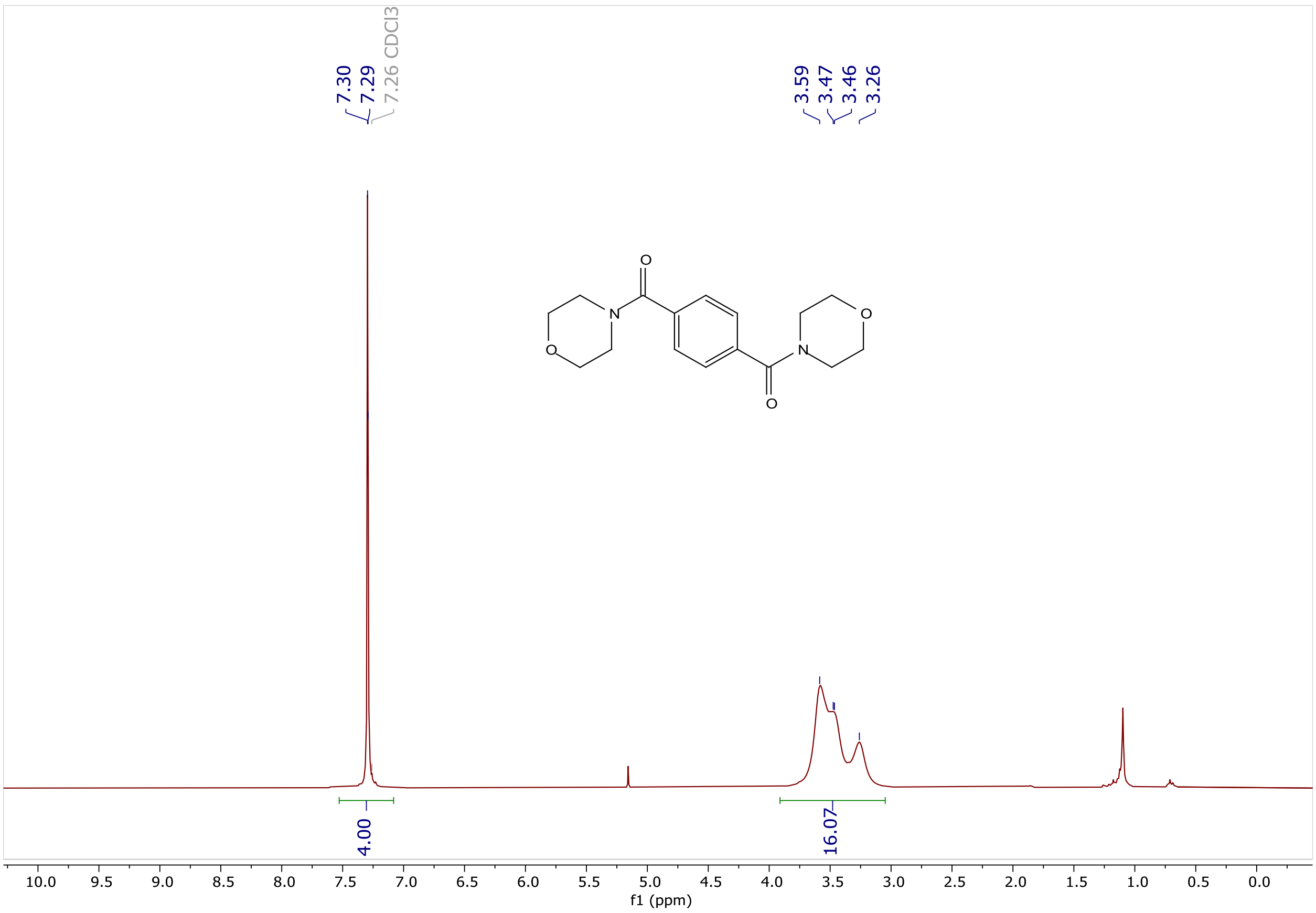

Figure S20. ^1H NMR spectrum ($CDCl_3$, 300 MHz, 297 K) of product obtained from postPET.

LDPE/OPP/PET blend

1.5 g of a polymer blend, containing PET, was ball milled with 1.5 mL of morpholine in the presence of MeONa as a catalyst and NaCl as grinding agent using a 25 mL SS jar and two 15 mm SS balls at 30 Hz for 60 minutes. 220 mg of PET upcycling product was obtained after complete conversion of PET present in the packet. NMR spectra of the packet and NMR spectra of unreacted polymer after reaction were recorded in TFA-d_1, where no PET peak was observed in the unreacted remaining mixture of polyolefins (LDPE and OPP) after reaction.

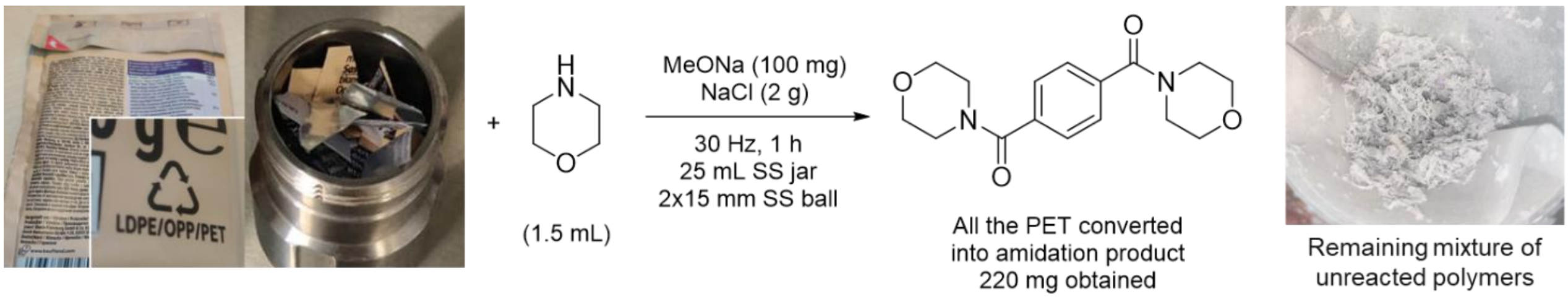

Scheme S4. Mechanochemical upcycling of LDPE/OPP/PET mixed polymer material.

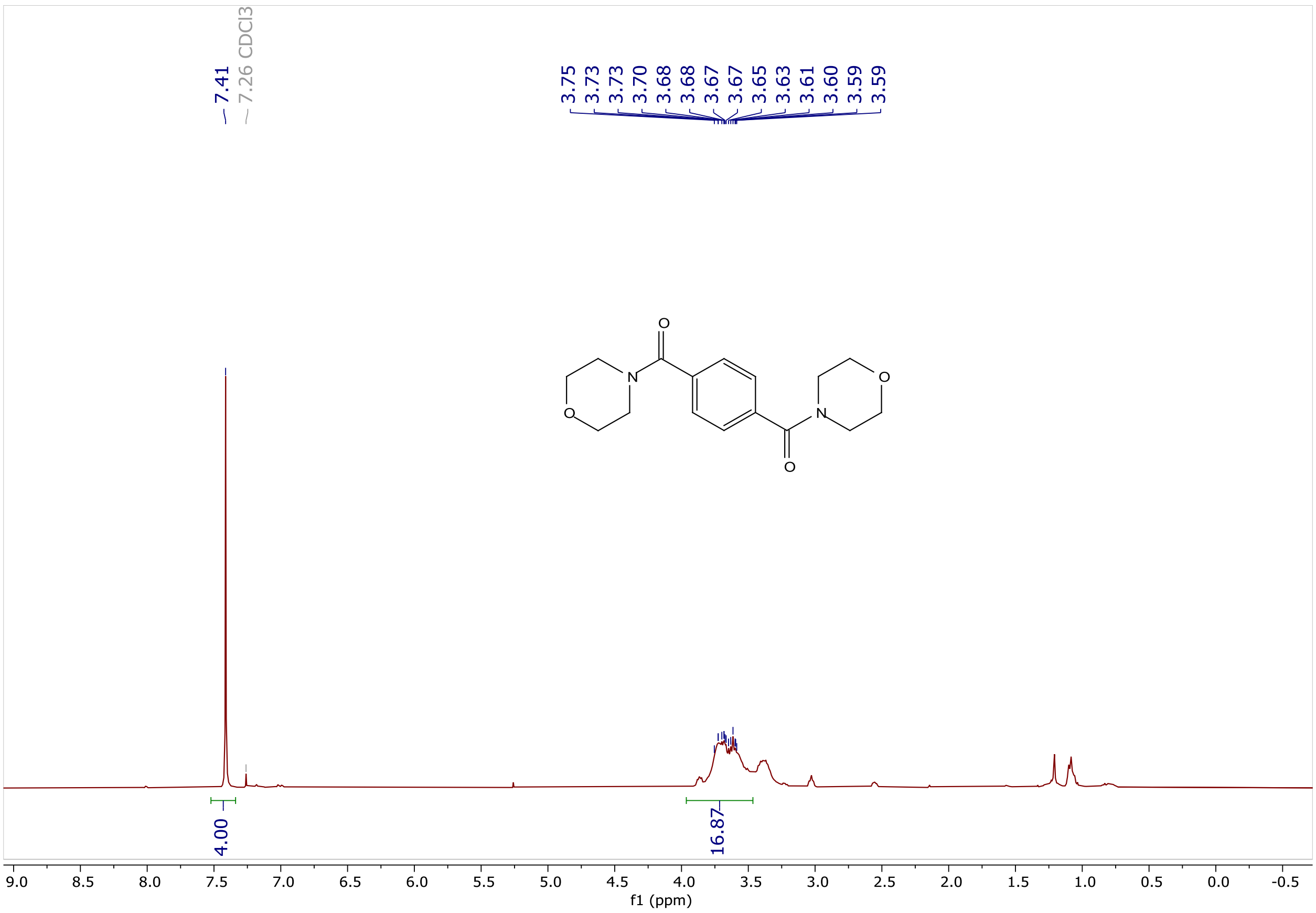

Figure S21. ^{1}H NMR spectrum ($CDCl_3$, 300 MHz, 297 K) of product obtained from LDPE/OPP/PET-based material.

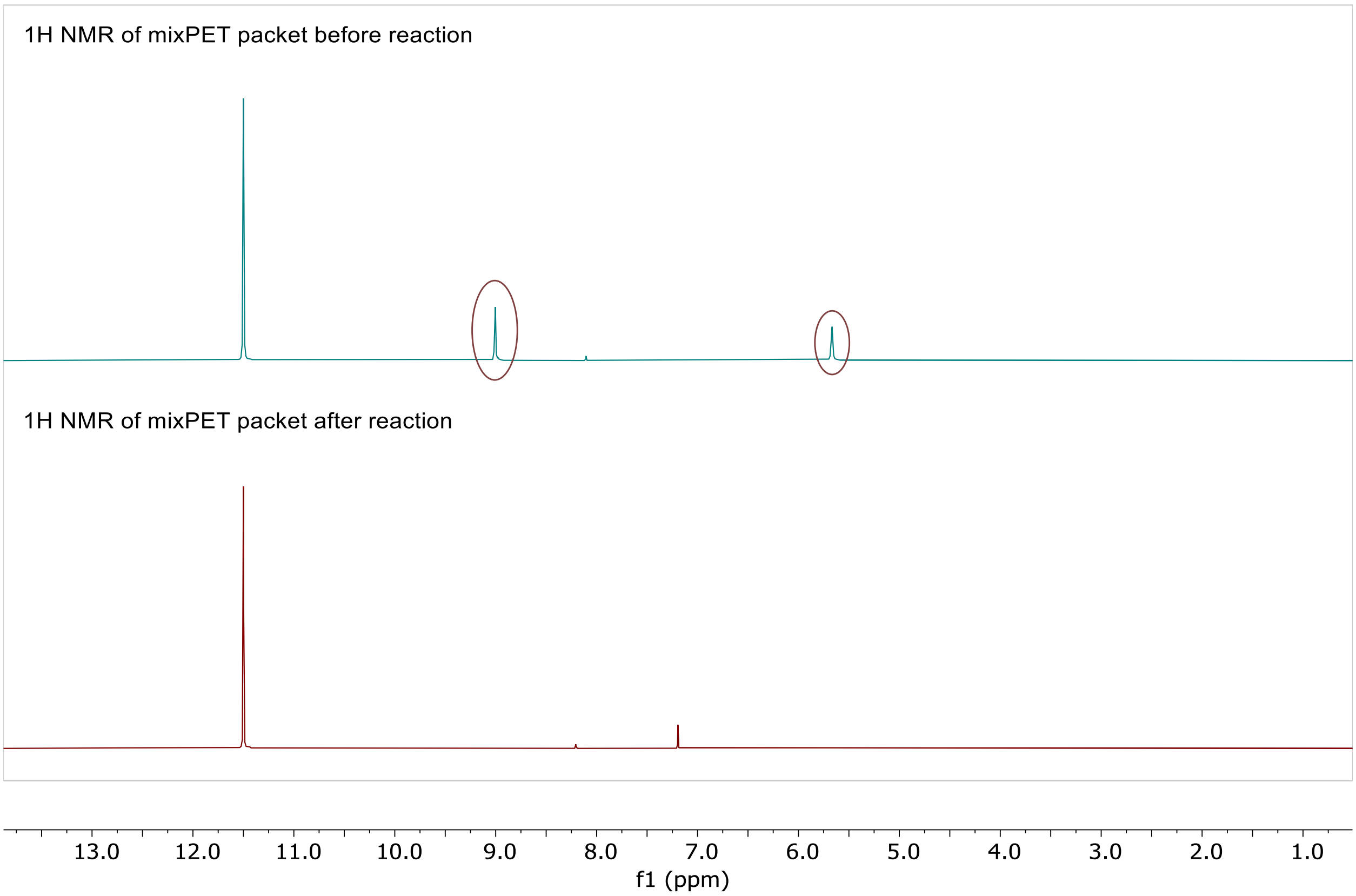

Figure S22. ^{1}H NMR spectra (TFA-d_1, 300 MHz, 297 K) of the LDPE/OPP/PET polymer blend before and after mechanochemical upcycling reaction. Circles indicate PET resonances.

PostPLA

1.5 g of a PLA pen (20.8 mmol) and 2.6 mL of 4-methoxybenzyl alcohol were ball milled together in the presence of MeONa (0.056 g, 5 mol%) using a 25 mL SS jar and a 15 mm SS ball at 30 Hz for 60 minutes. 3.6 g of a colourless liquid were obtained after the reaction, containing PLA alcoholysis product and some of the remaining alcohol. All the PLA from the pen material was upcycled in this reaction, indicated by its NMR spectrum (Figure S23).

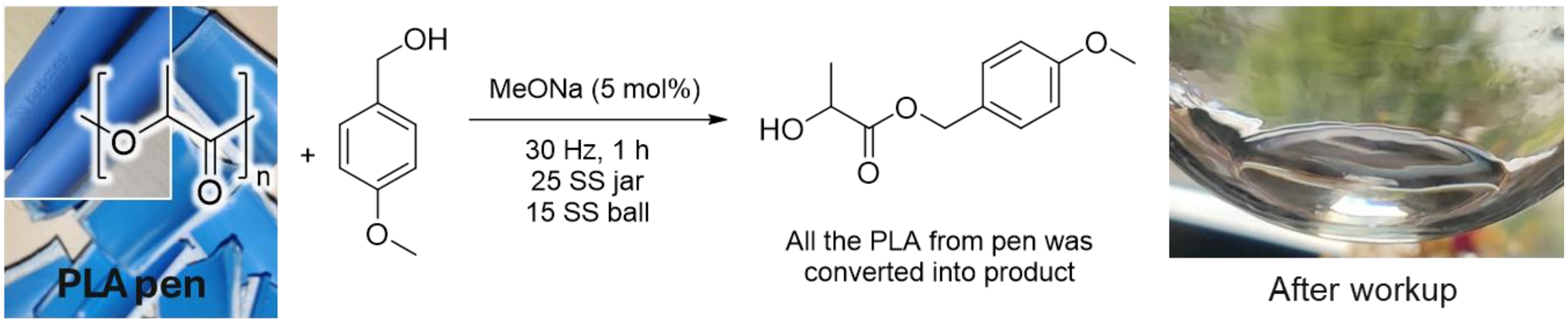

Scheme S5. Mechanochemical upcycling of PLA pen material.

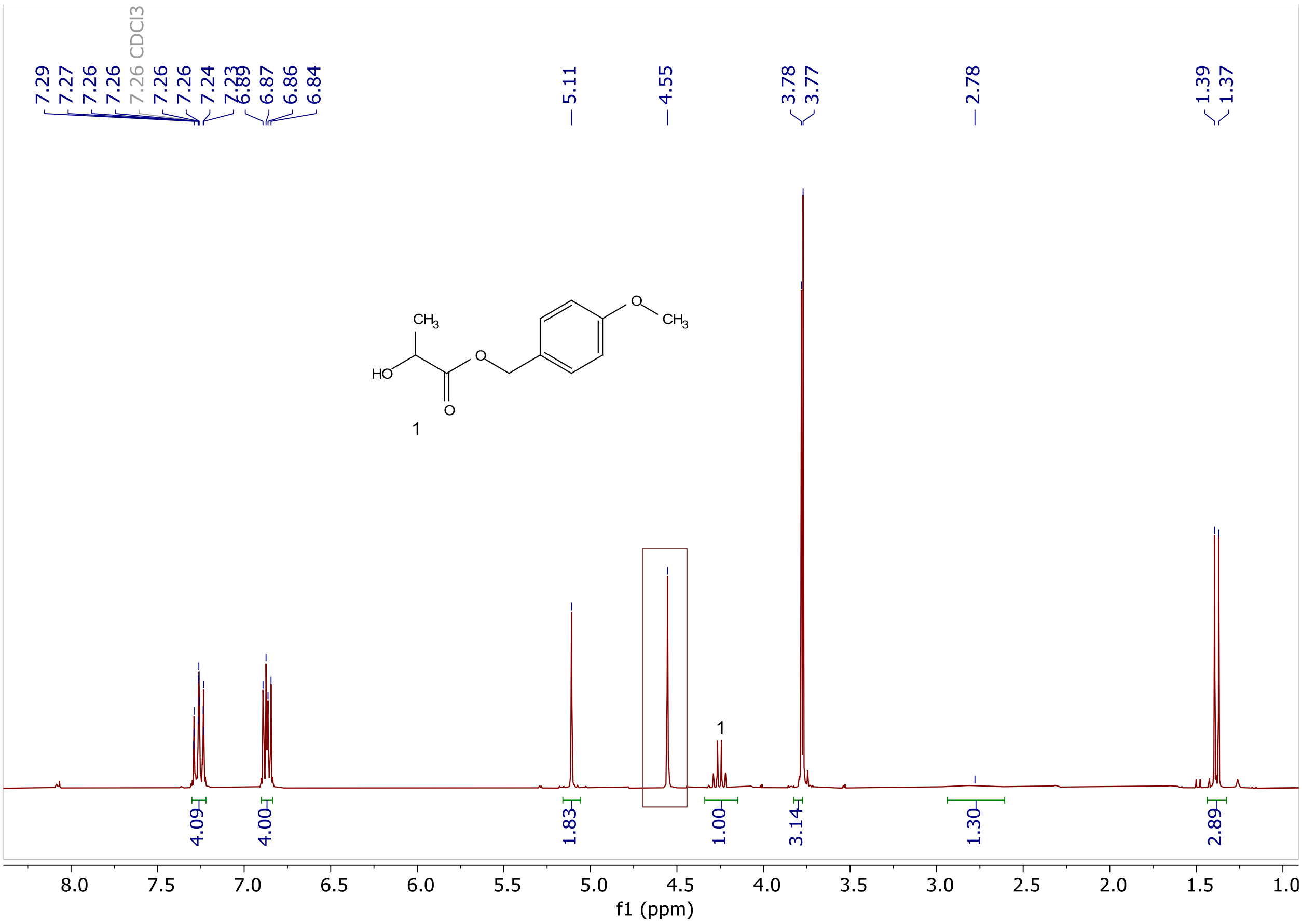

Figure S23. ^{1}H NMR spectrum ($CDCl_3$, 300 MHz, 297 K) of alcoholysis product obtained from PLA pen in $CDCl_3$. During the reaction, an excess amount of alcohol was used which is also visible in this NMR spectrum, indicated as dotted square and extra peaks around at δ 3.78, 6.8, 7.25 ppm. There is no peak around 5.1 ppm which indicates that all PLA from the pen was consumed.

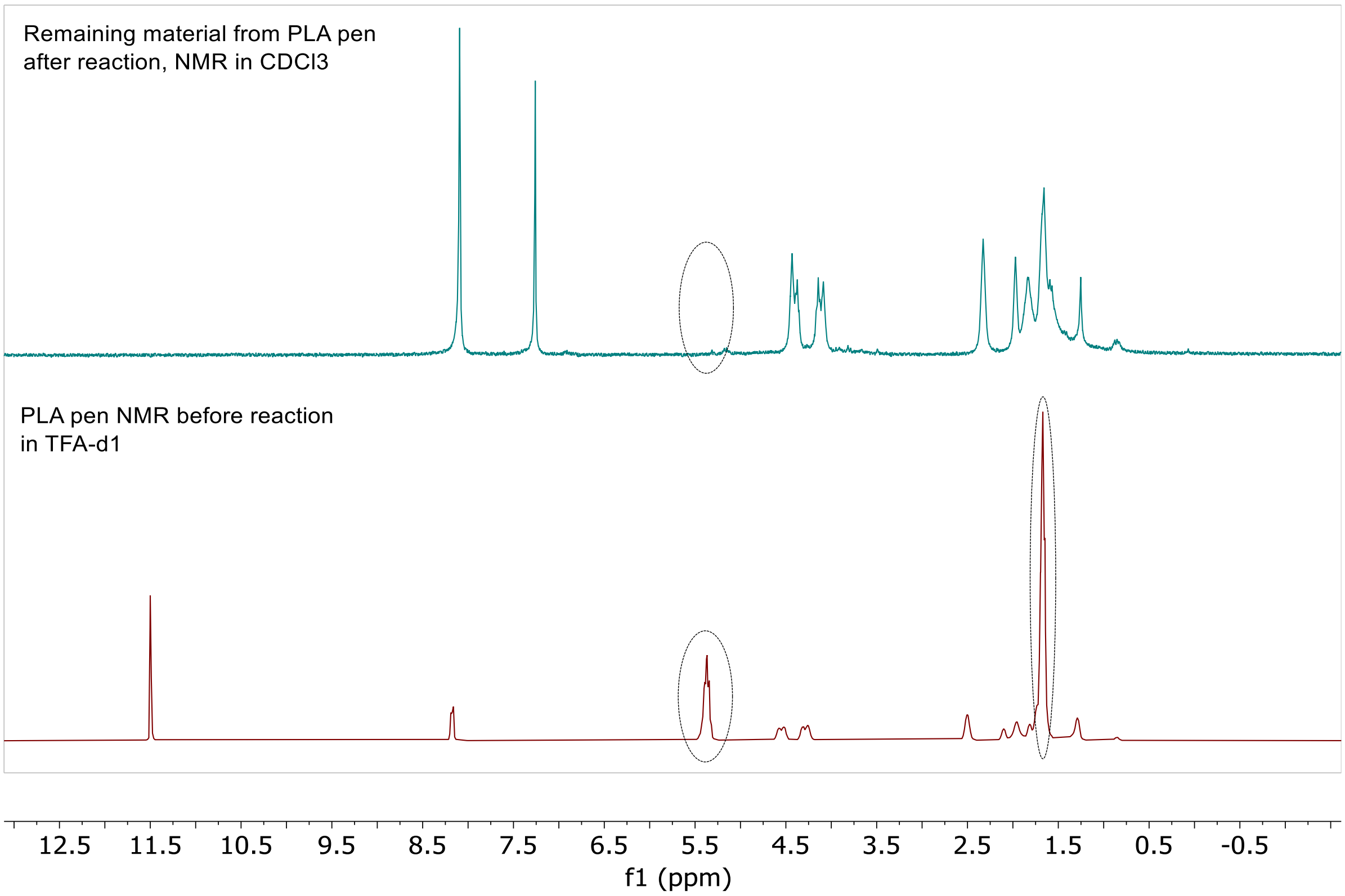

Figure S24. ^{1}H NMR spectra ($CDCl_3$, 300 MHz, 297 K) of PLA pen before and after mechanochemical upcycling reaction. Circles indicate PLA resonances.

Large-scale mechanochemical upcycling of polyesters

2.5 g PEF, 20 mol% MeONa, and 30 mmol 2-ethyl hexanol were transferred into a 25 mL SS ball milling jar, and ball milled for 60 minutes using two 15 mm SS balls at 30 Hz. After completion of the reaction, all PEF was converted into DEHF. The reaction mixture was quenched with water and extracted using diethyl ether, yielding 92% of pure DEHF product as a colourless liquid.

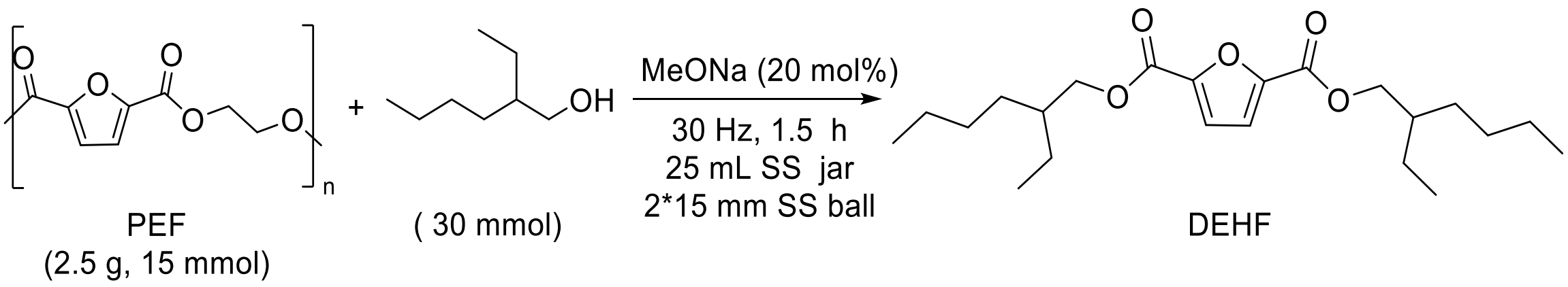

Scheme S6. Large scale demonstration of alcoholysis of PEF.

1.92 g PET, 20 mol% MeONa, and 30 mmol 4-methoxybenzyl alcohol were transferred into a 25 mL SS ball milling jar, and ball milled for 90 minutes using two 15 mm SS balls at 30 Hz. After completion of the reaction, all PEF was converted into product. The reaction mixture was washed with brine and water and filtered. The white solid residue of pure product was collected on a filter paper and dried at 60 °C overnight, yielding 93% of pure bis(4-methoxybenzyl) terephthalate.

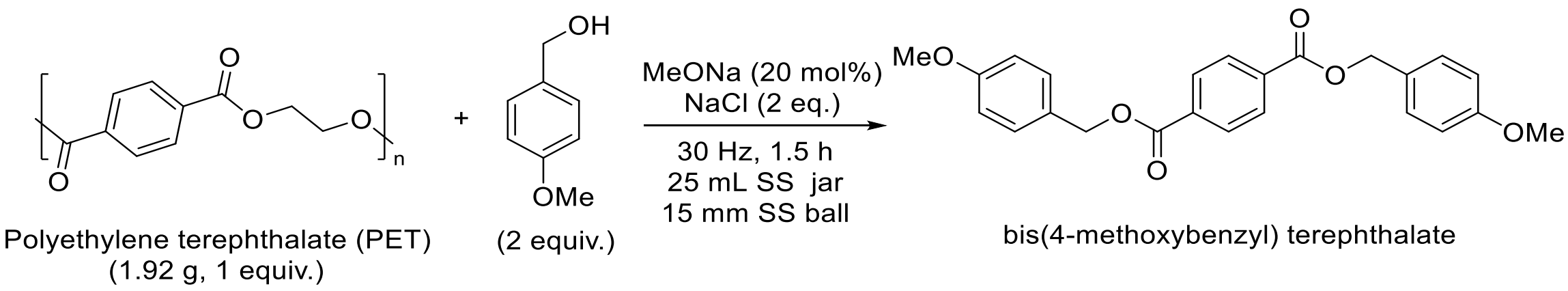

Scheme S7. Large scale demonstration of alcoholysis of PET.

Large-scale mechanochemical upcycling reaction of PLA (2 g) was performed using morpholine and 4-methyxobenzyl alcohol to obtain the corresponding amidation and alcoholysis products, respectively. After completion of the reaction, all PLA was converted into products in 60 minutes using 25 mL SS ball milling jar and a 15 mm SS.

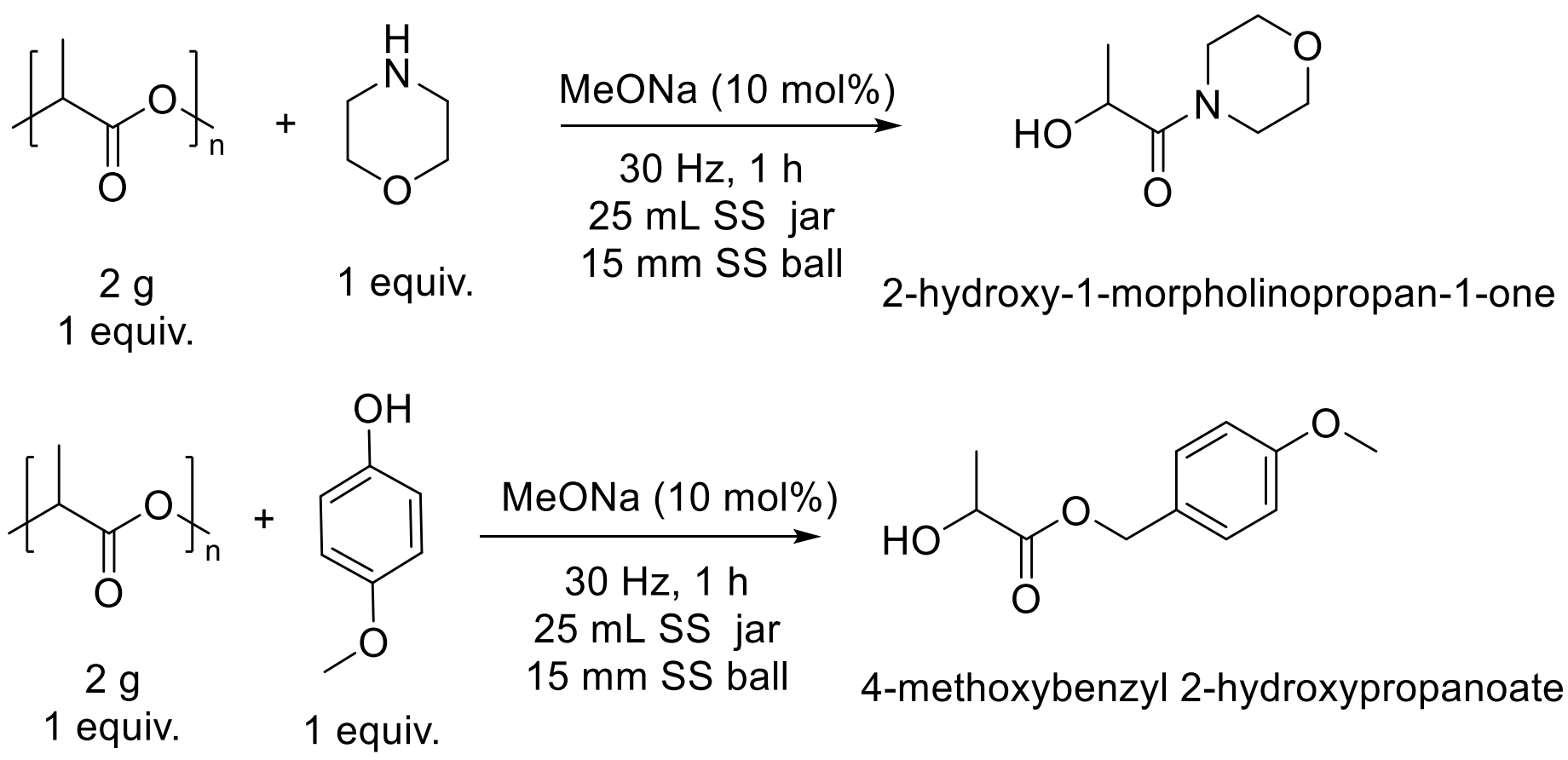

Scheme S8. Large scale demonstration of alcoholysis and amidation of PLA.

NMR spectra of depolymerisation products

Mechanochemical upcycling of PEF

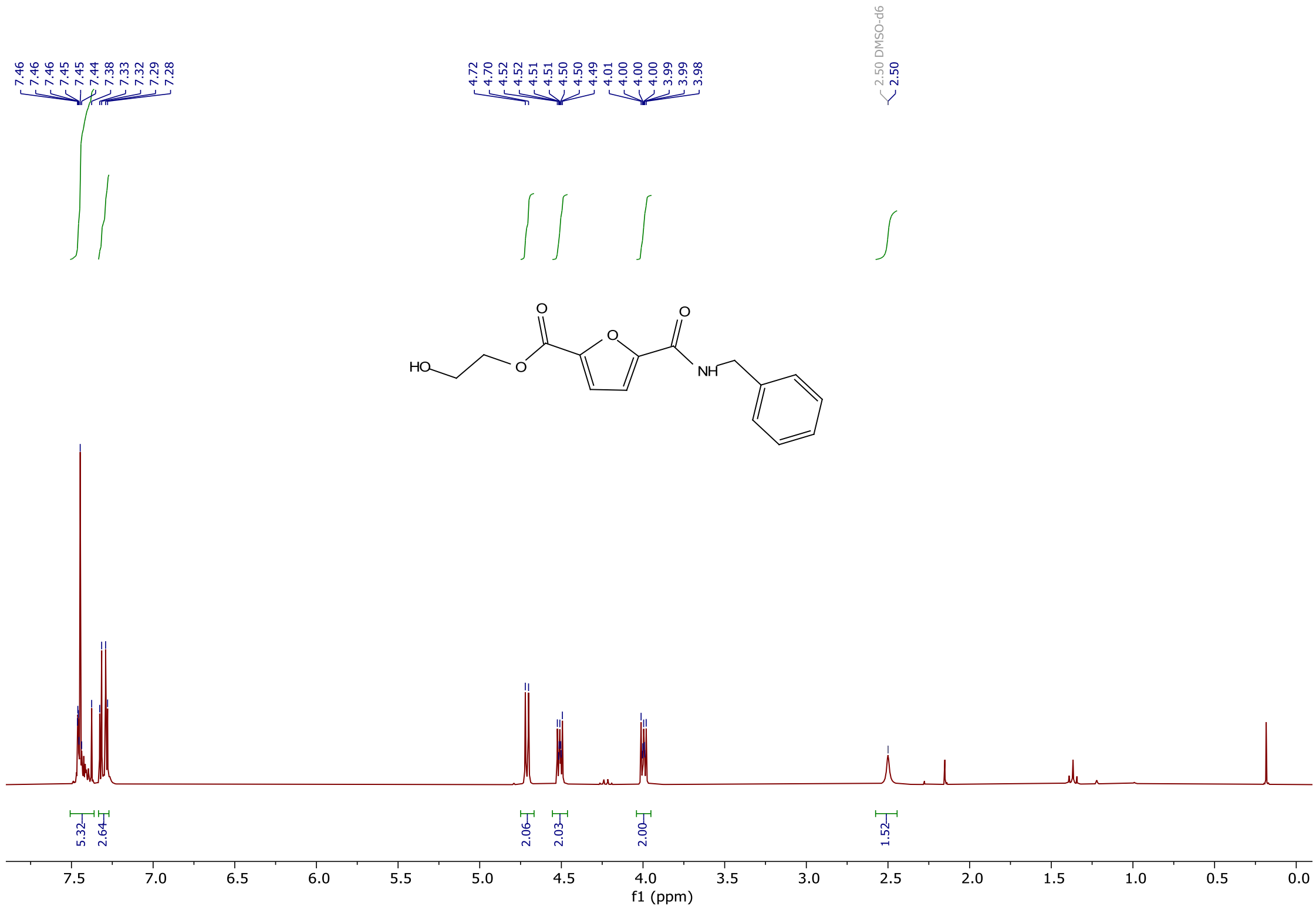

Figure S25. ^{1}H NMR spectrum (DMSO-d_6, 300 MHz, 297 K) of 2-hydroxyethyl 5-(benzylcarbamoyl)furan-2-carboxylateobtained from amidation of PEF in the absence of sodium methoxide.

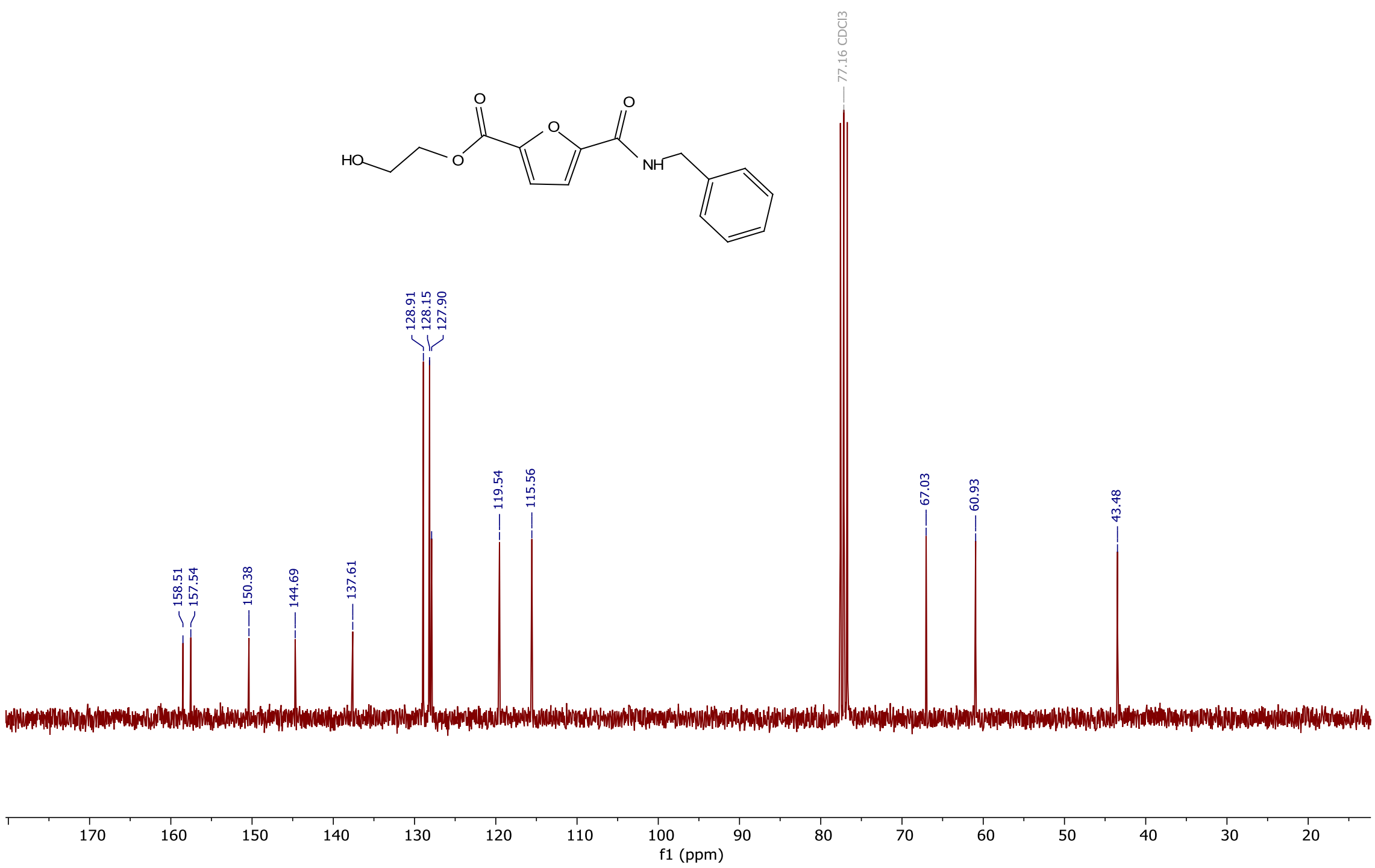

Figure S26. ^{13}C NMR spectrum (DMSO-d_6, 75 MHz, 297 K) of 2-hydroxyethyl 5-(benzylcarbamoyl)furan-2-carboxylate obtained from amidation of PEF in the absence of sodium methoxide.

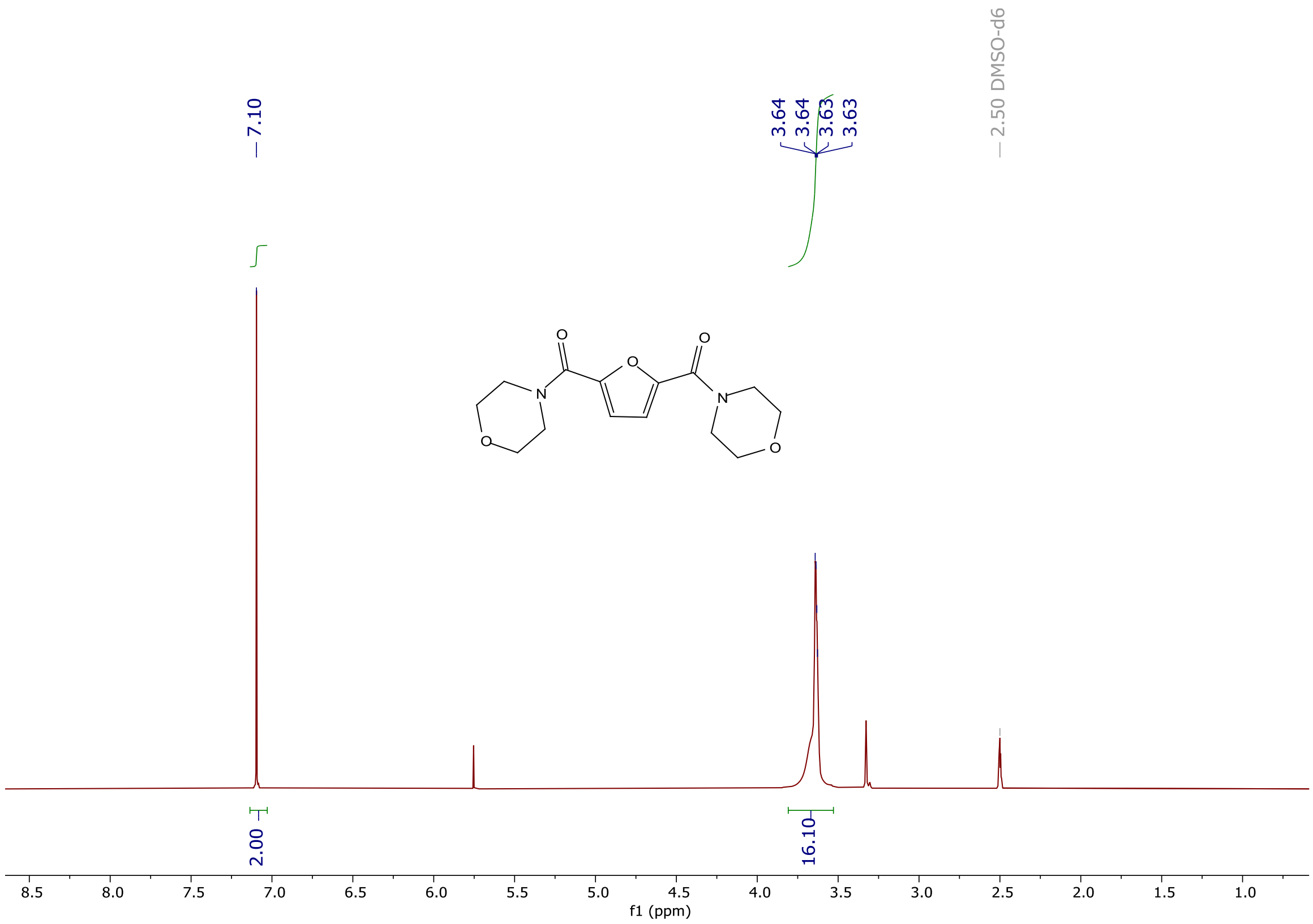

Figure S27. ^{1}H NMR spectrum (DMSO-d_6, 400 MHz, 297 K) of furan-2,5-diylbis(morpholinomethanone) (**3a**).

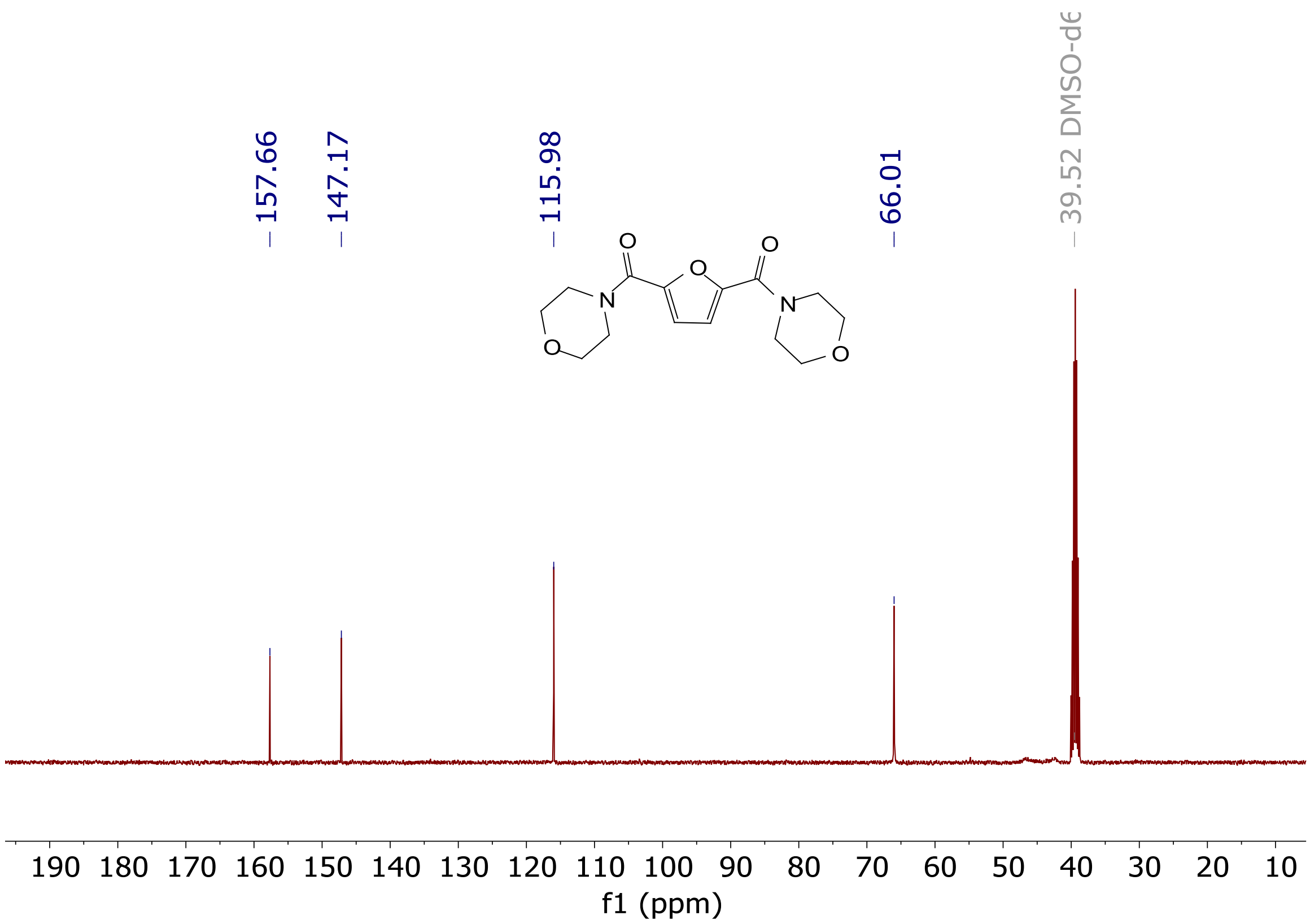

Figure S28. ^{13}C NMR spectrum (DMSO-d_6, 101 MHz, 297 K) of furan-2,5-diylbis(morpholinomethanone) (**3a**).

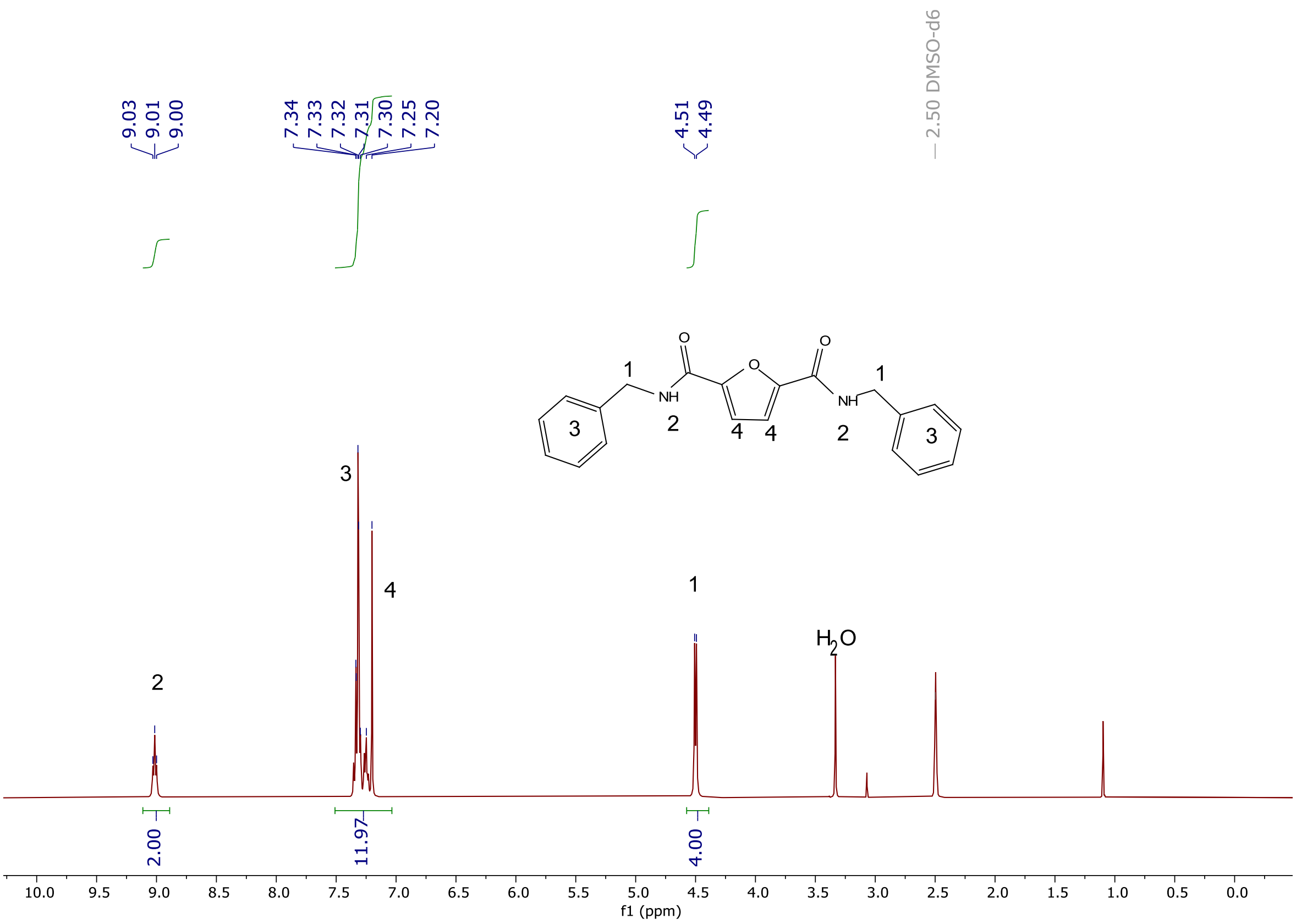

Figure S29. ^{1}H NMR spectrum (DMSO-d_6, 400 MHz, 297 K) of N^2,N^5-dibenzylfuran-2,5-dicarboxamide (**3b**).

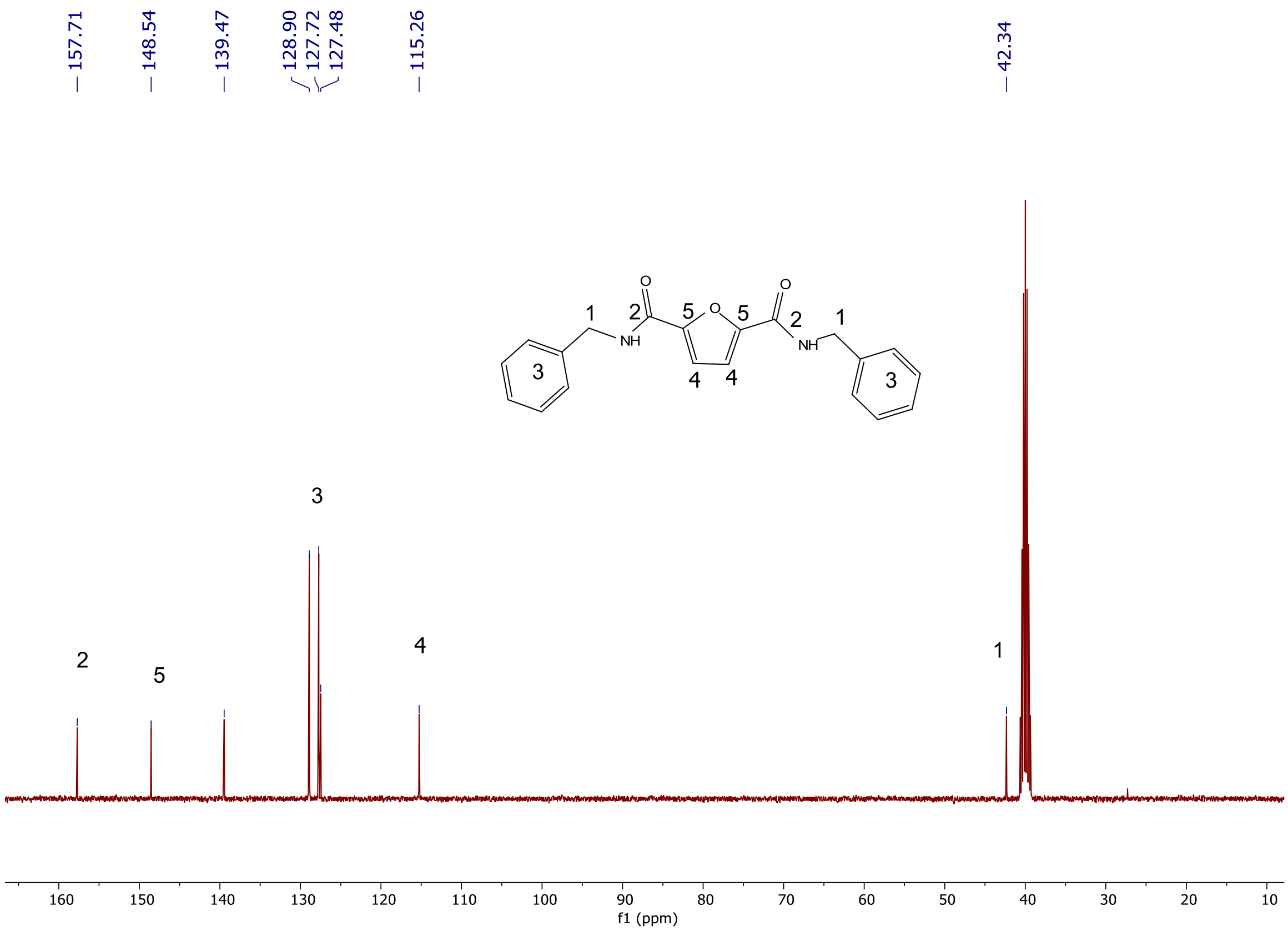

Figure S30. ^{13}C NMR spectrum (DMSO-d_6, 101 MHz, 297 K) of N^2,N^5-dibenzylfuran-2,5-dicarboxamide (**3b**).

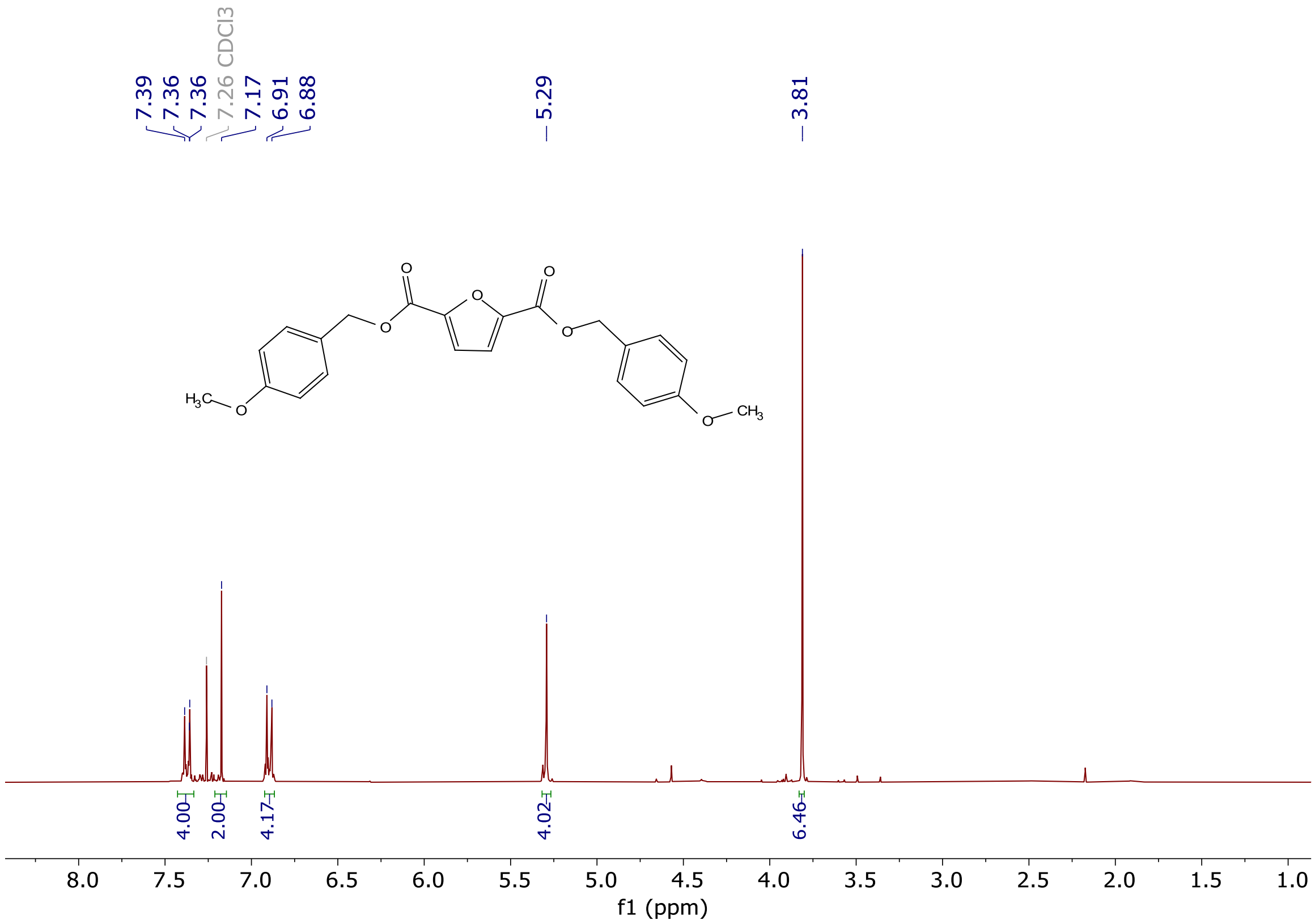

Figure S31. ^{1}H NMR spectrum ($CDCl_3$, 300 MHz, 297 K) of bis(4-methoxybenzyl) furan-2,5-dicarboxylate (**3c**).

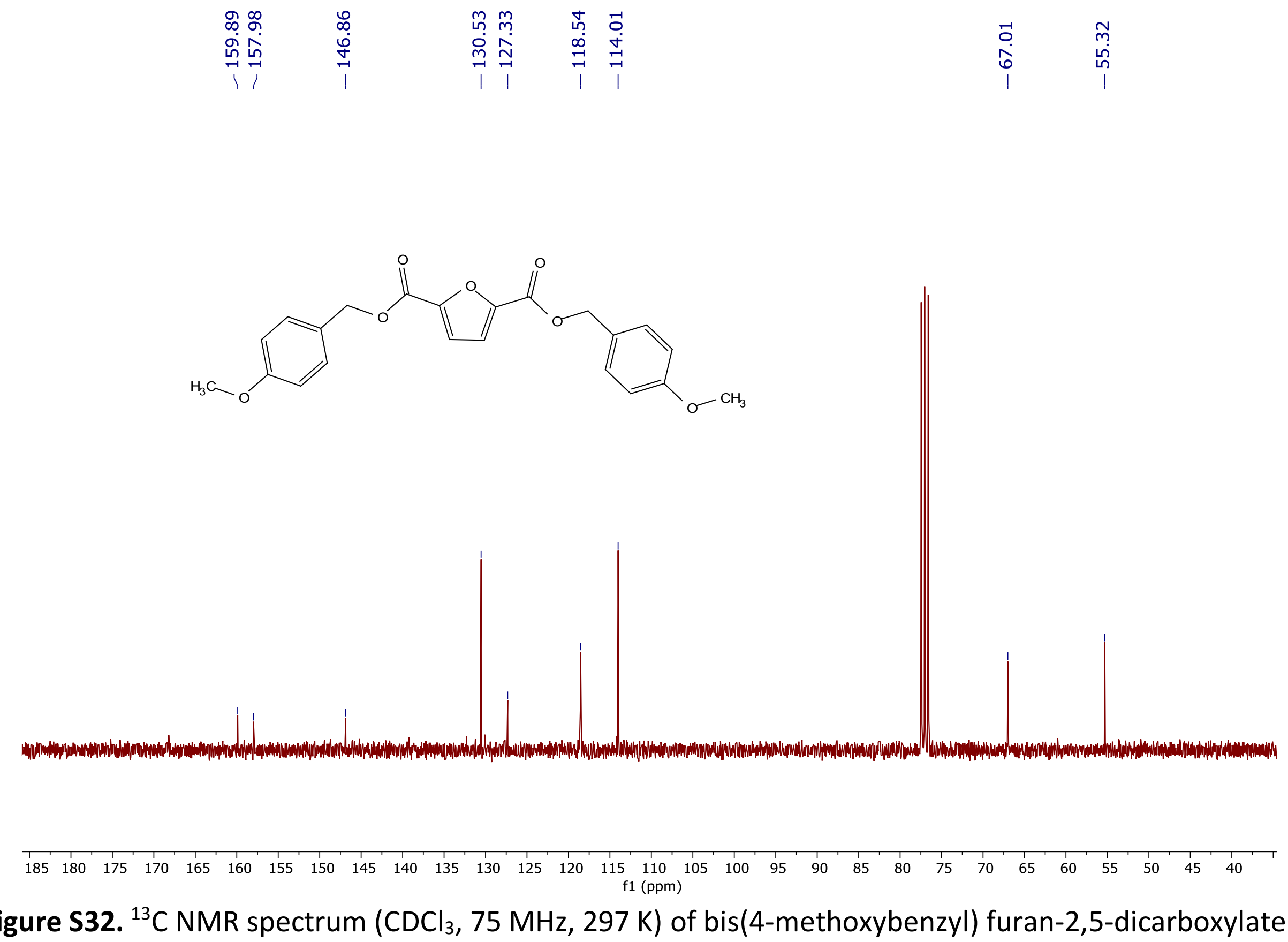

Figure S32. ^{13}C NMR spectrum ($CDCl_3$, 75 MHz, 297 K) of bis(4-methoxybenzyl) furan-2,5-dicarboxylate (**3c**).

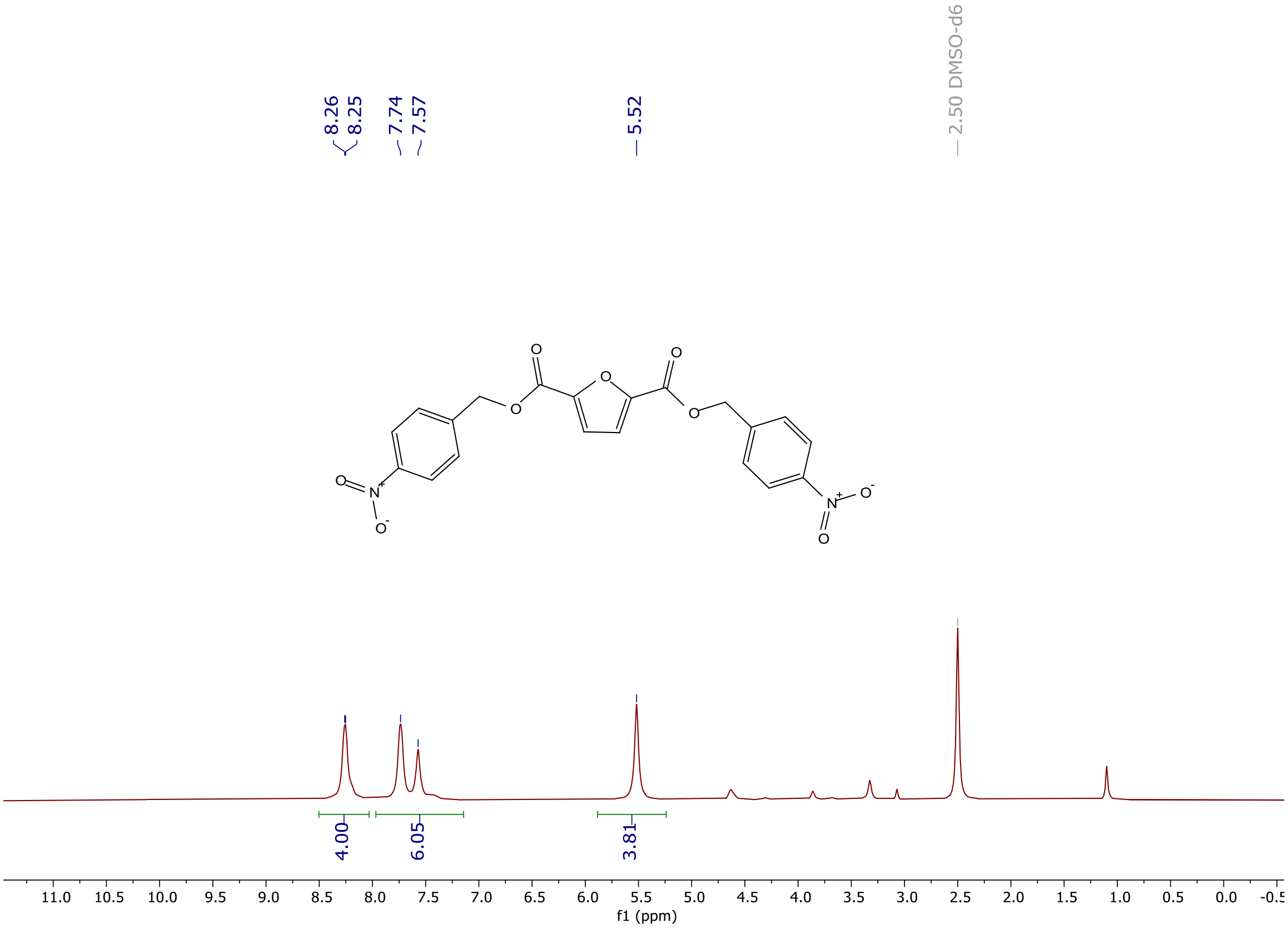

Figure S33. ^{1}H NMR spectrum (DMSO-d_6, 400 MHz, 297 K) of bis(4-nitrobenzyl) furan-2,5-dicarboxylate (**3d**).

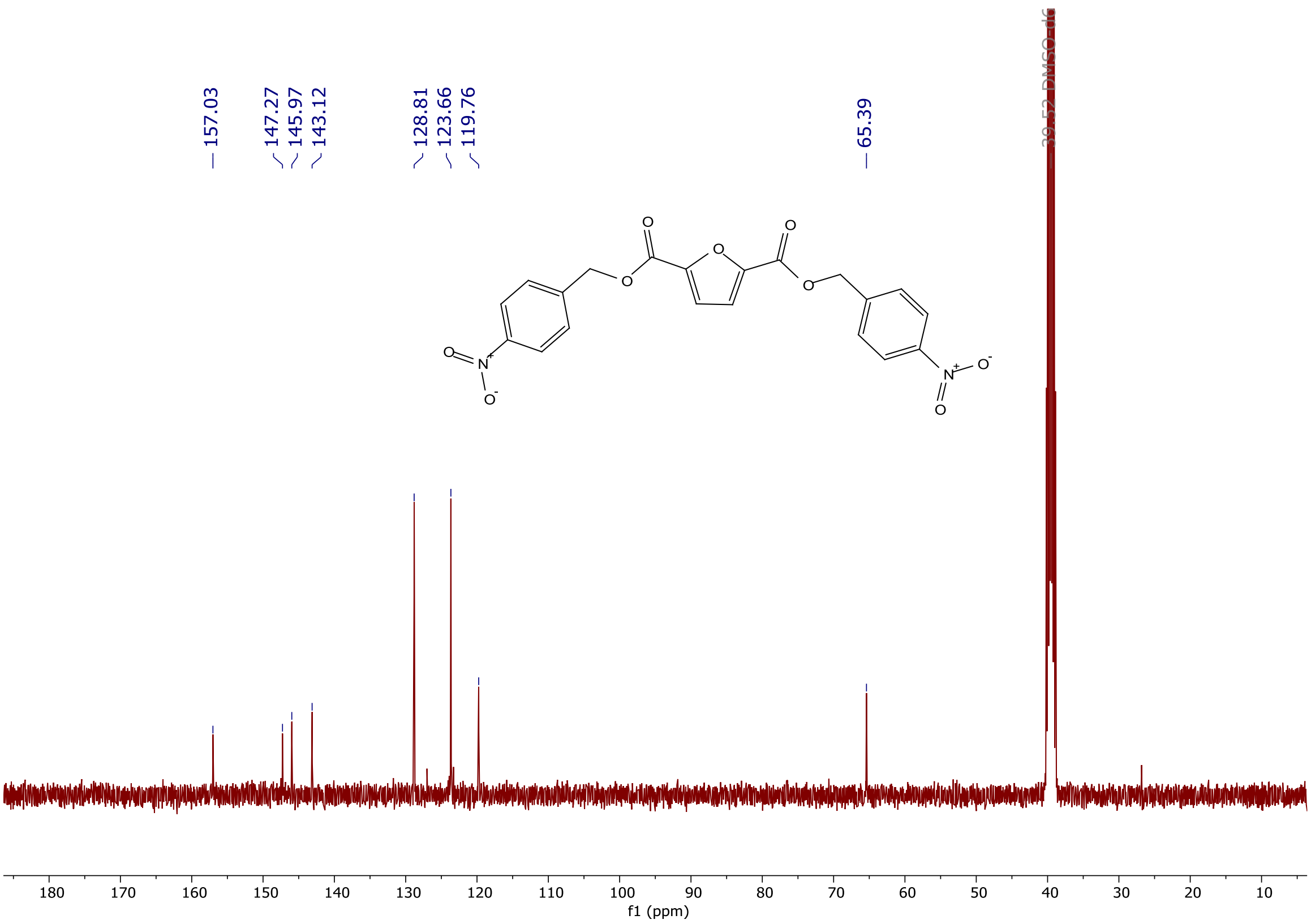

Figure S34. ^{13}C NMR spectrum (DMSO-d_6, 101 MHz, 297 K) of bis(4-nitrobenzyl) furan-2,5-dicarboxylate (**3d**).

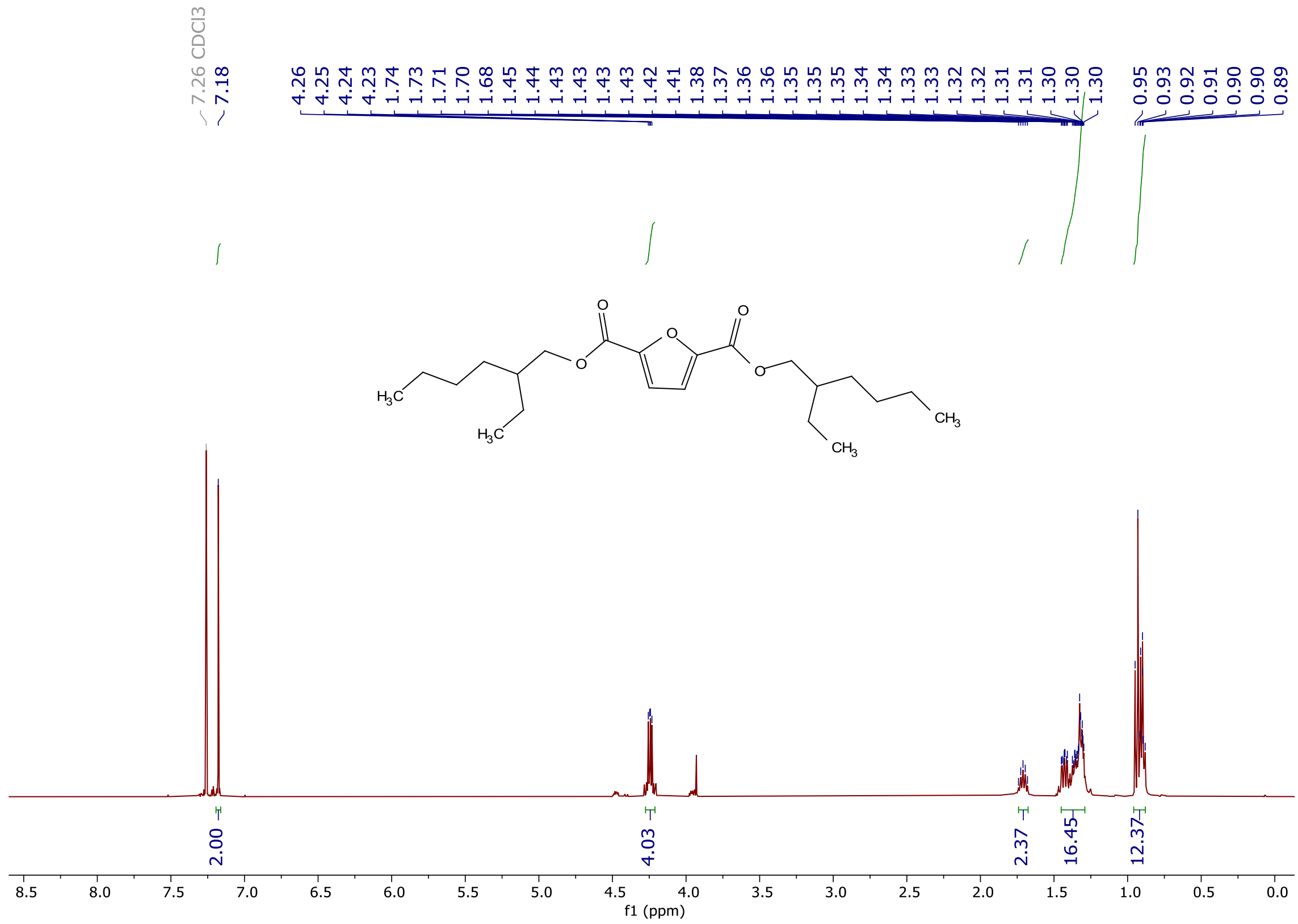

Figure S35. ^{1}H NMR spectrum ($CDCl_3$, 400 MHz, 297 K) of bis(2-ethylhexyl) furan-2,5-dicarboxylate (**3e**).

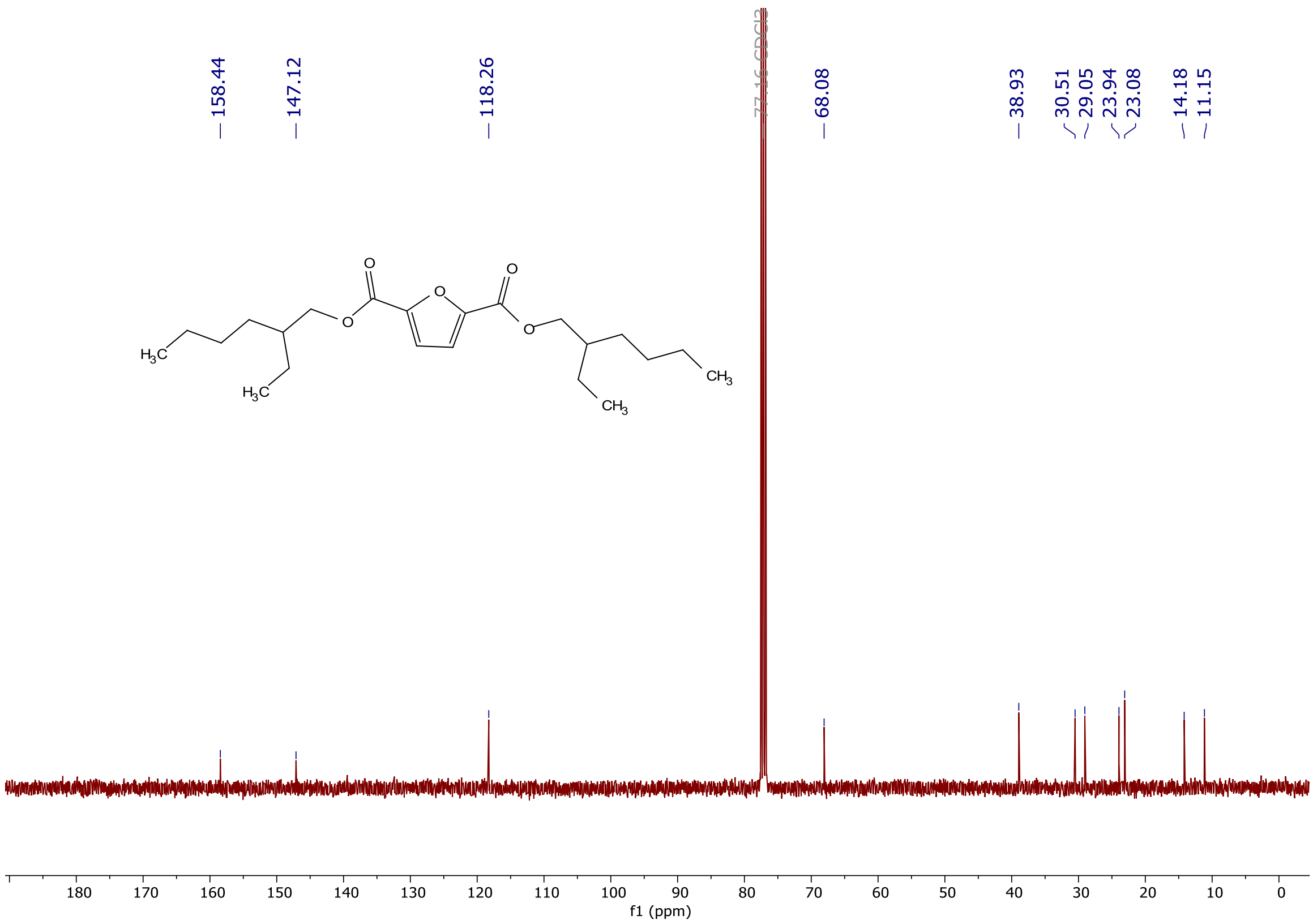

Figure S36. ^{13}C NMR spectrum ($CDCl_3$, 101 MHz, 297 K) of bis(2-ethylhexyl) furan-2,5-dicarboxylate (**3e**).

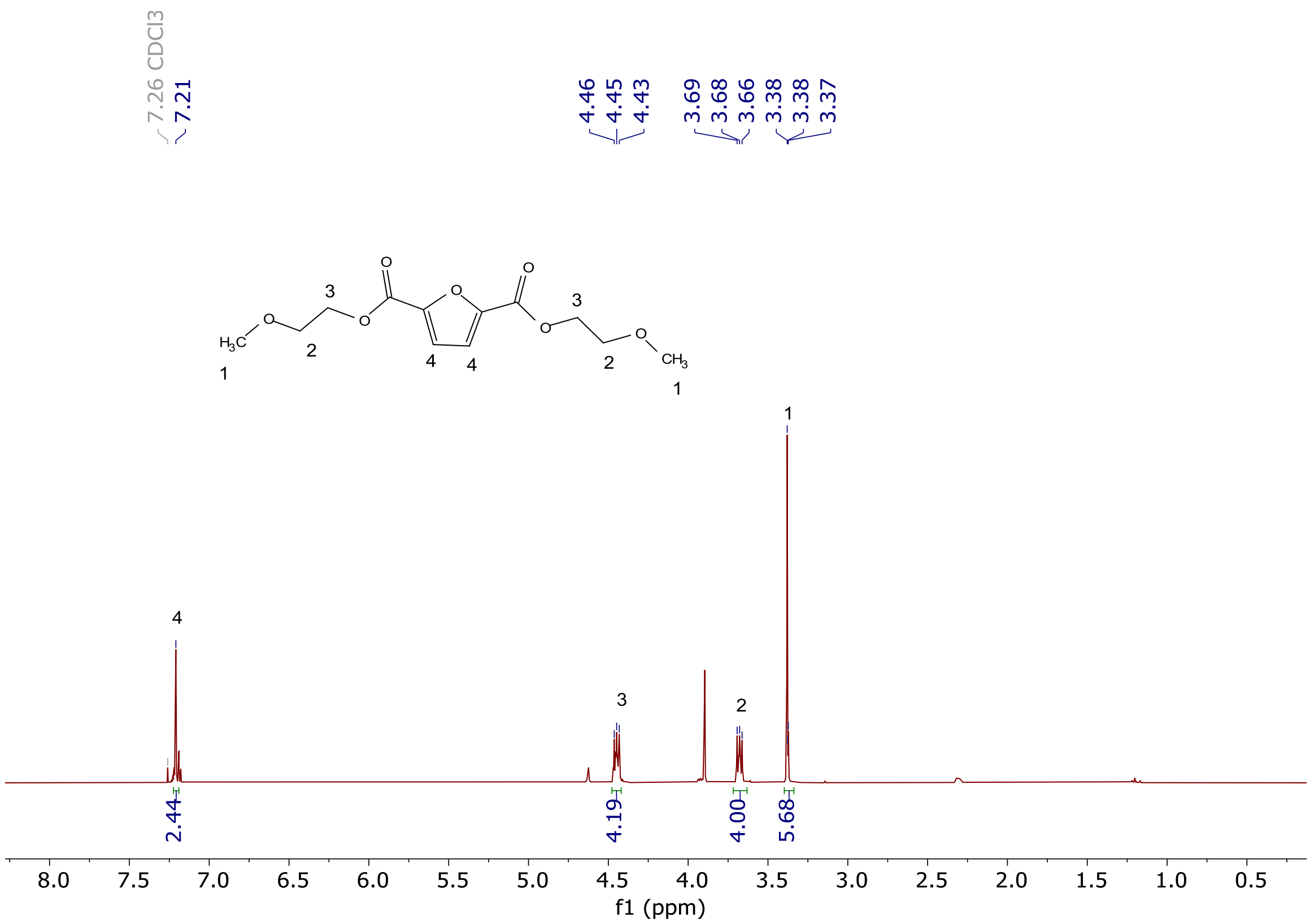

Figure S37. ^{1}H NMR spectrum ($CDCl_3$, 400 MHz, 297 K) of bis(2-methoxyethyl) furan-2,5-dicarboxylate (**3f**).

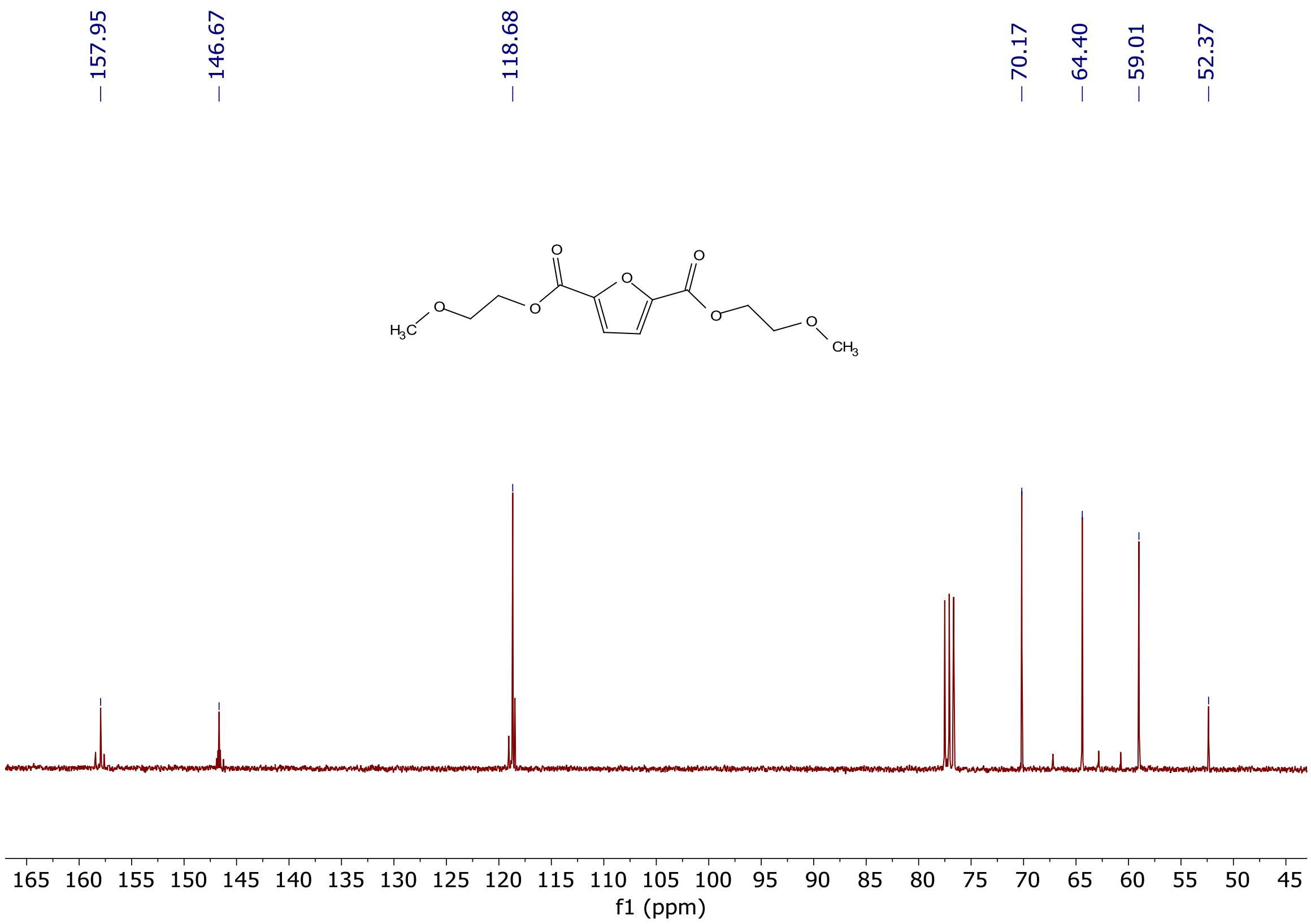

Figure S38. ^{13}C NMR spectrum ($CDCl_3$, 101 MHz, 297 K) of bis(2-methoxyethyl) furan-2,5-dicarboxylate (**3f**).

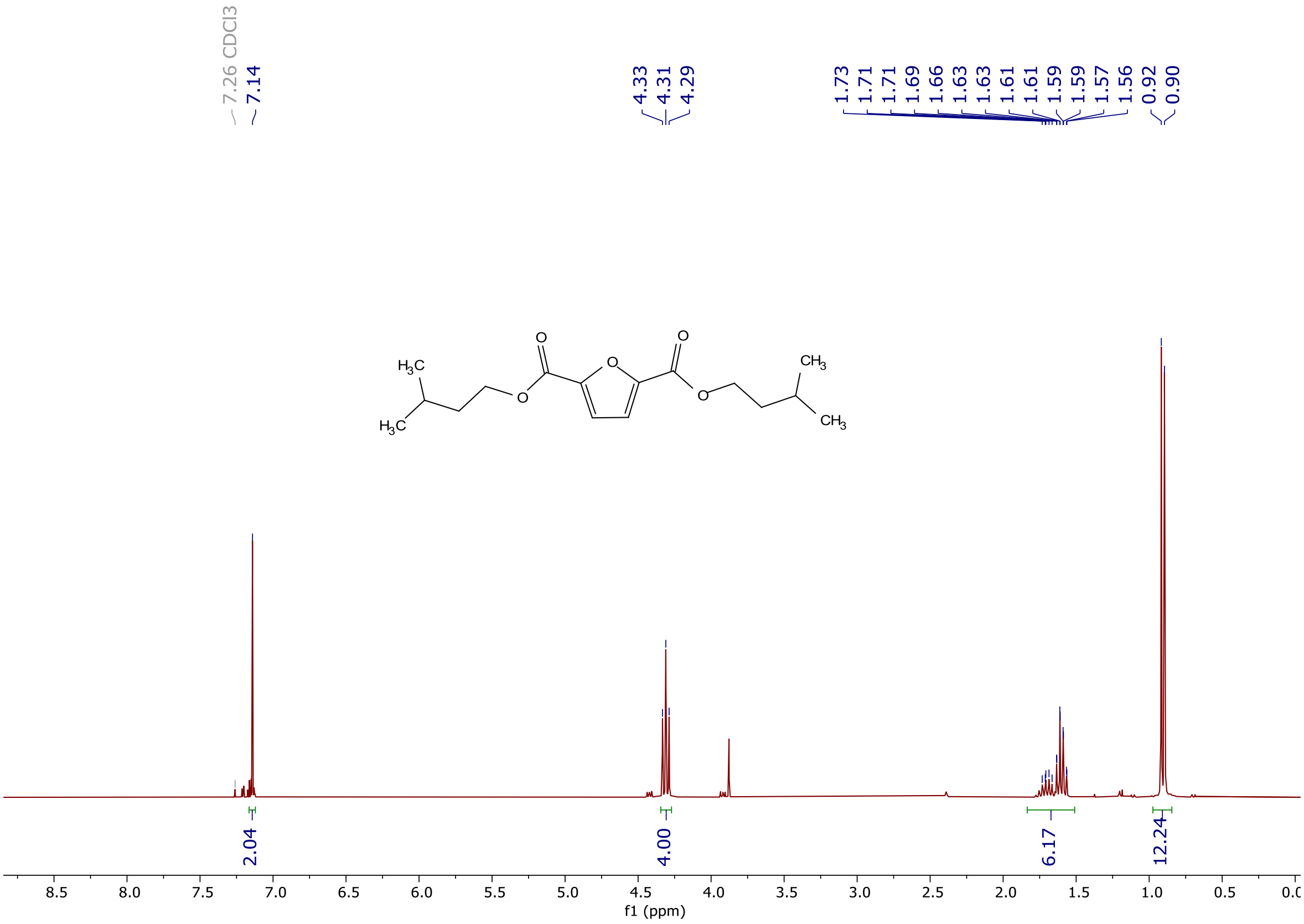

Figure S39. ^{1}H NMR spectrum ($CDCl_3$, 400 MHz, 297 K) of diisopentyl furan-2,5-dicarboxylate (**3g**).

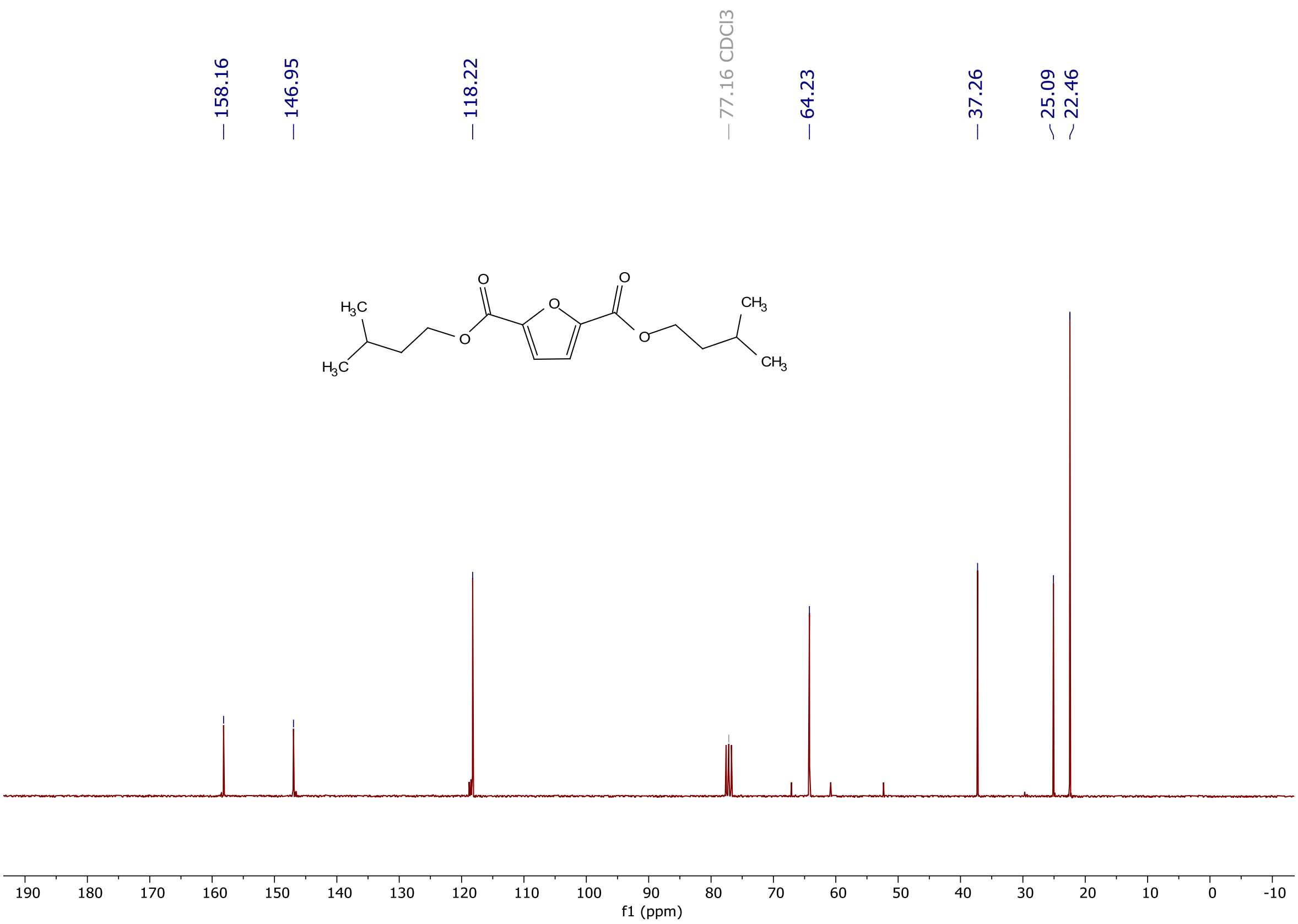

Figure S40. ^{13}C NMR spectrum ($CDCl_3$, 101 MHz, 297 K) of diisopentyl furan-2,5-dicarboxylate (**3g**).

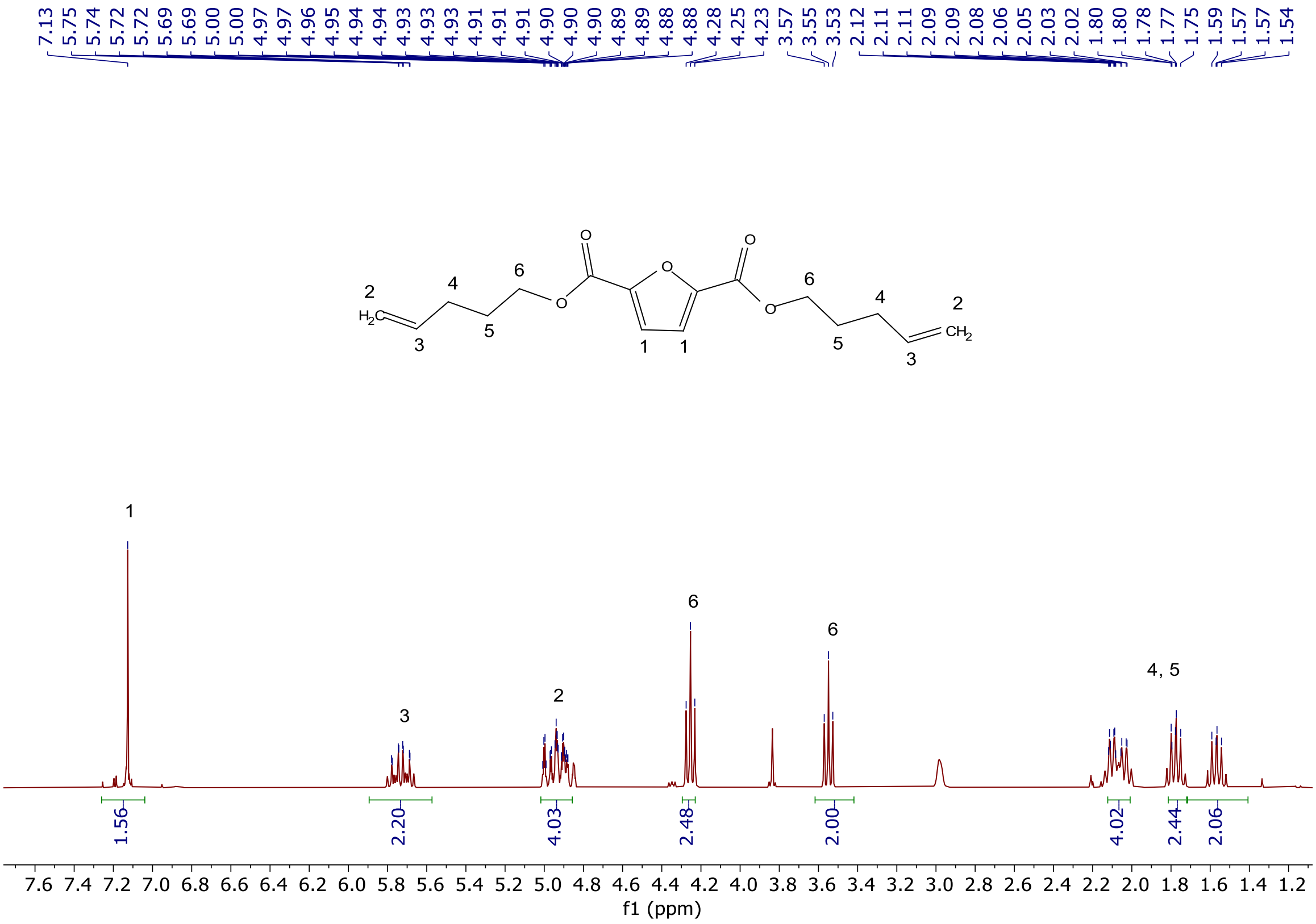

Figure S41. ^1H NMR spectrum ($CDCl_3$, 300 MHz, 297 K) of di(pent-4-en-1-yl) furan-2,5-dicarboxylate (**3h**).

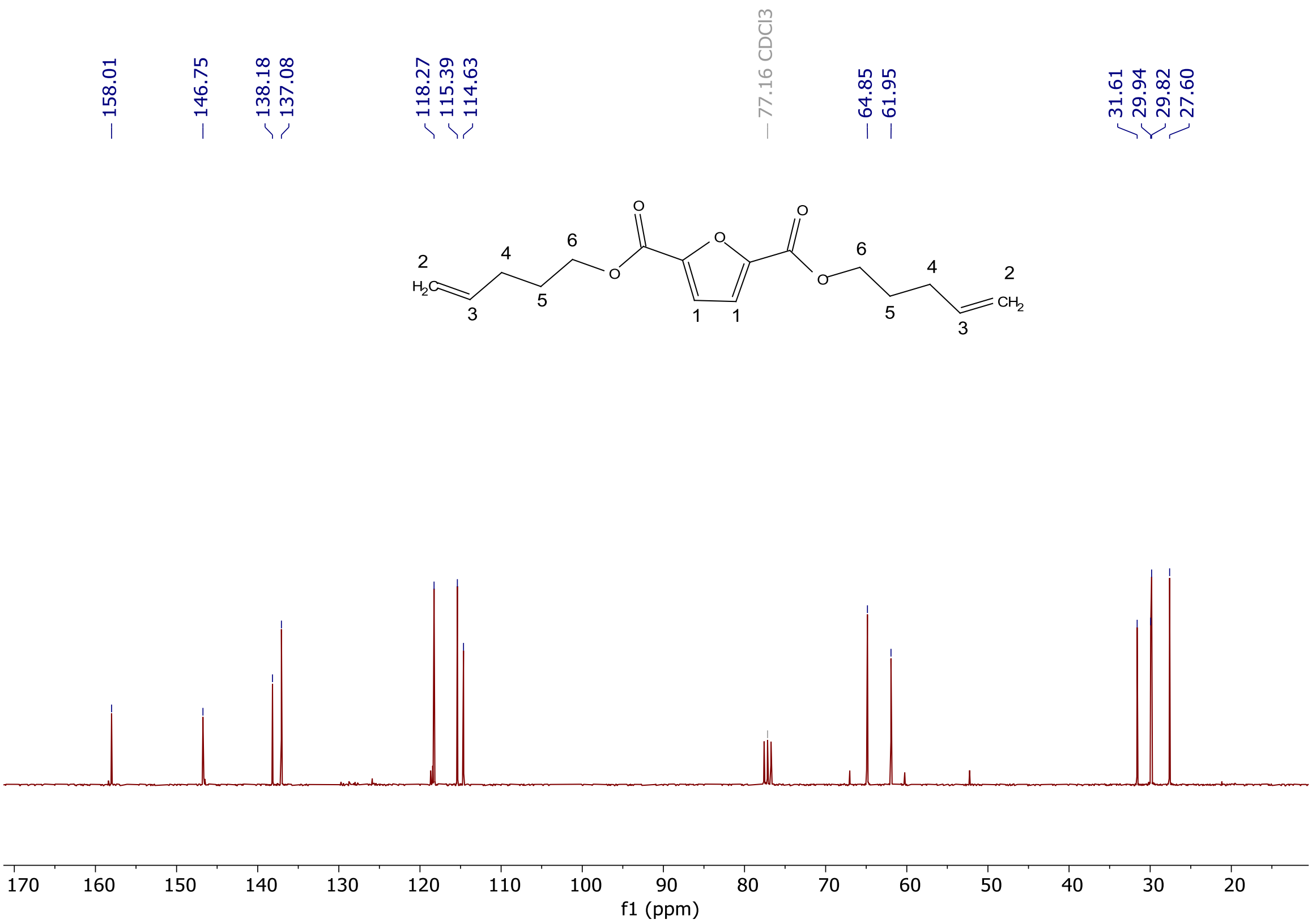

Figure S42. ^{13}C NMR spectrum ($CDCl_3$, 75 MHz, 297 K) of di(pent-4-en-1-yl) furan-2,5-dicarboxylate (**3h**).

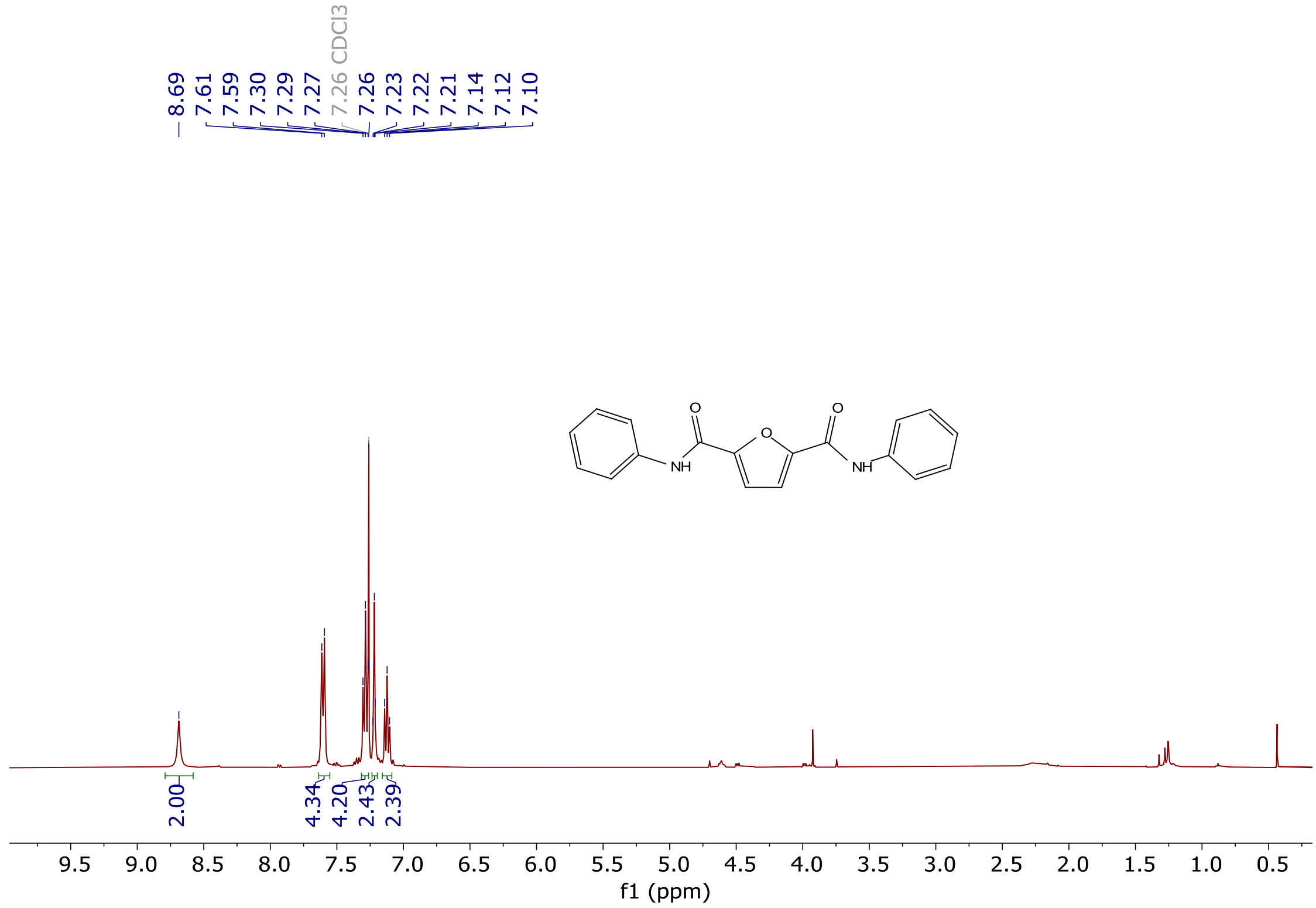

Figure S43. ^{1}H NMR spectrum ($CDCl_3$, 400 MHz, 297 K) of N^2,N^5-diphenylfuran-2,5-dicarboxamide (**3l**).

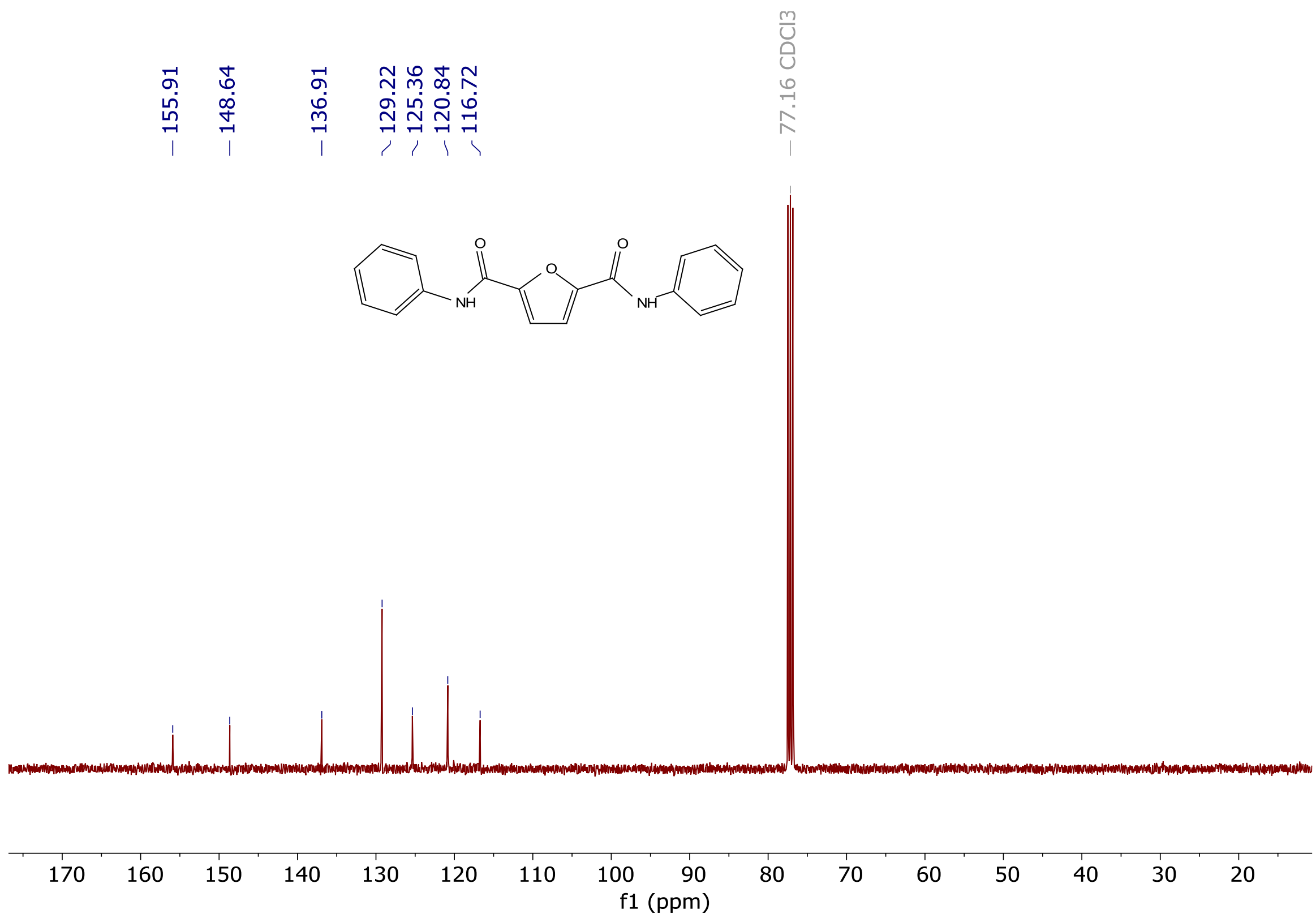

Figure S44. ^{13}C NMR spectrum ($CDCl_3$, 101 MHz, 297 K) of N^2,N^5-diphenylfuran-2,5-dicarboxamide (**3l**).

Mechanochemical upcycling of PBF

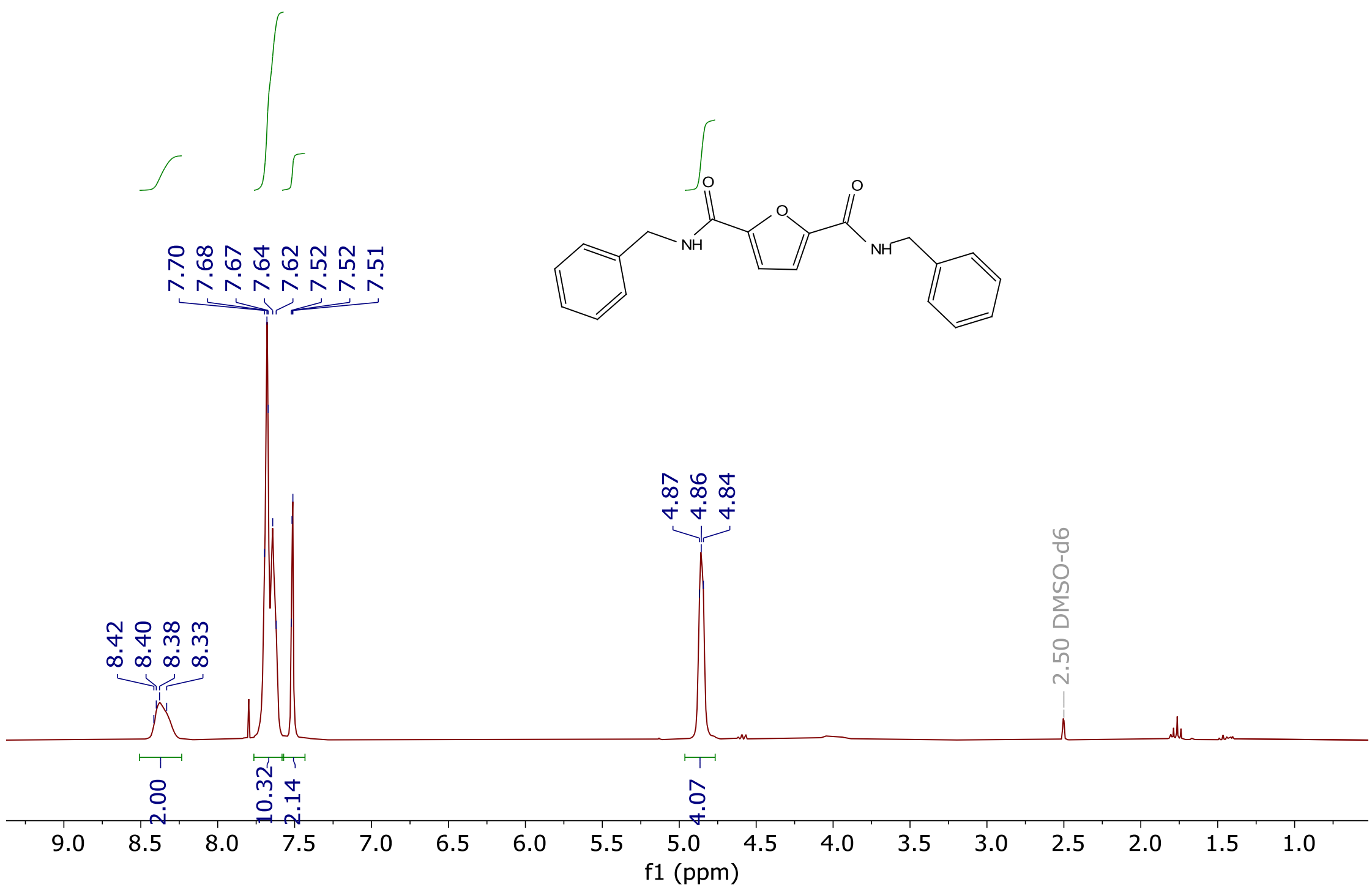

Figure S45. ^{1}H NMR spectrum (DMSO-d$_6$, 400 MHz, 297 K) of N^{2},N^{5}-dibenzylfuran-2,5-dicarboxamide (**4a**) from PBF.

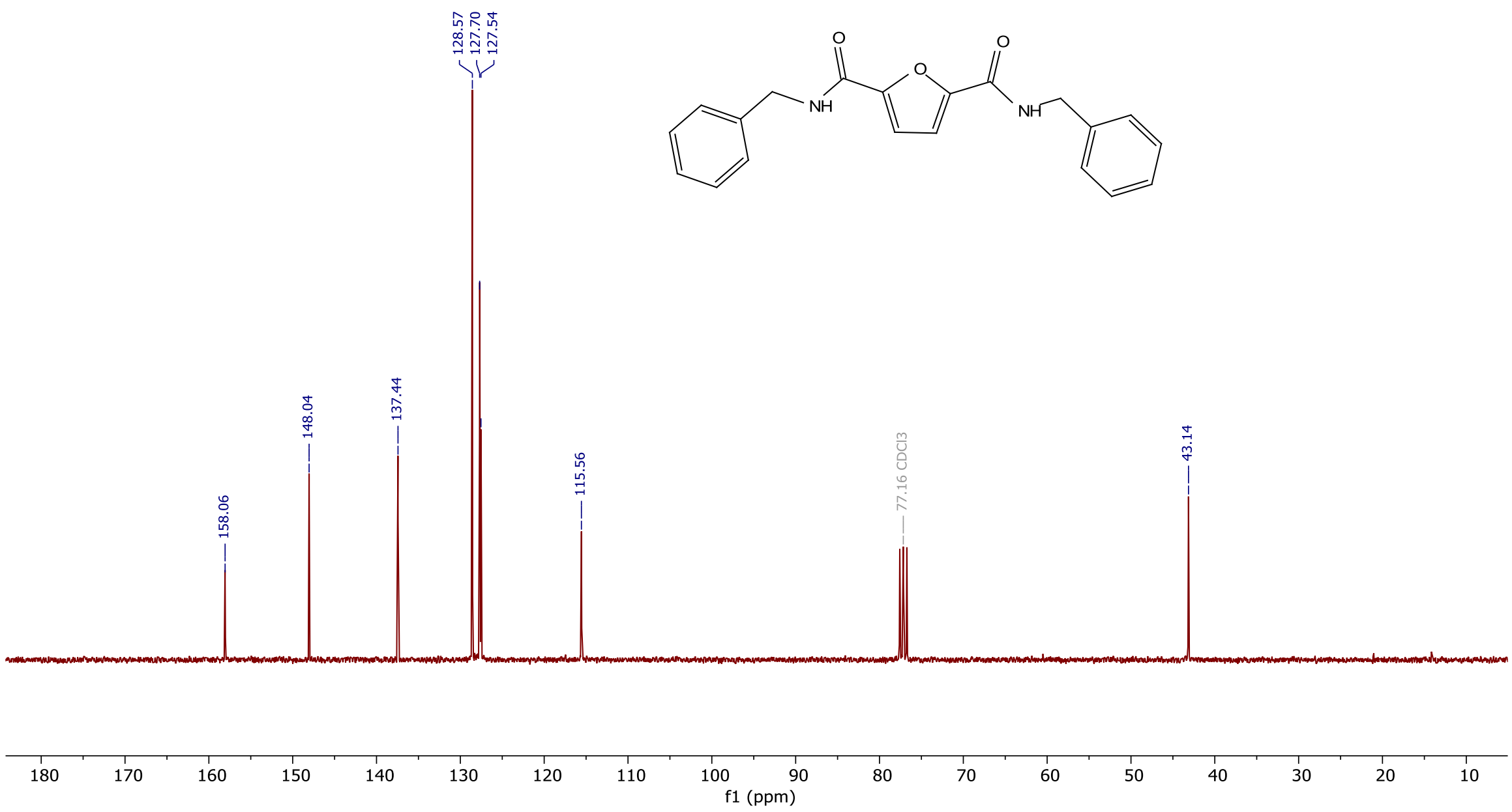

Figure S46. ^{13}C NMR spectrum (DMSO-d$_6$, 101 MHz, 297 K) of N^{2},N^{5}-dibenzylfuran-2,5-dicarboxamide (**4a**) from PBF.

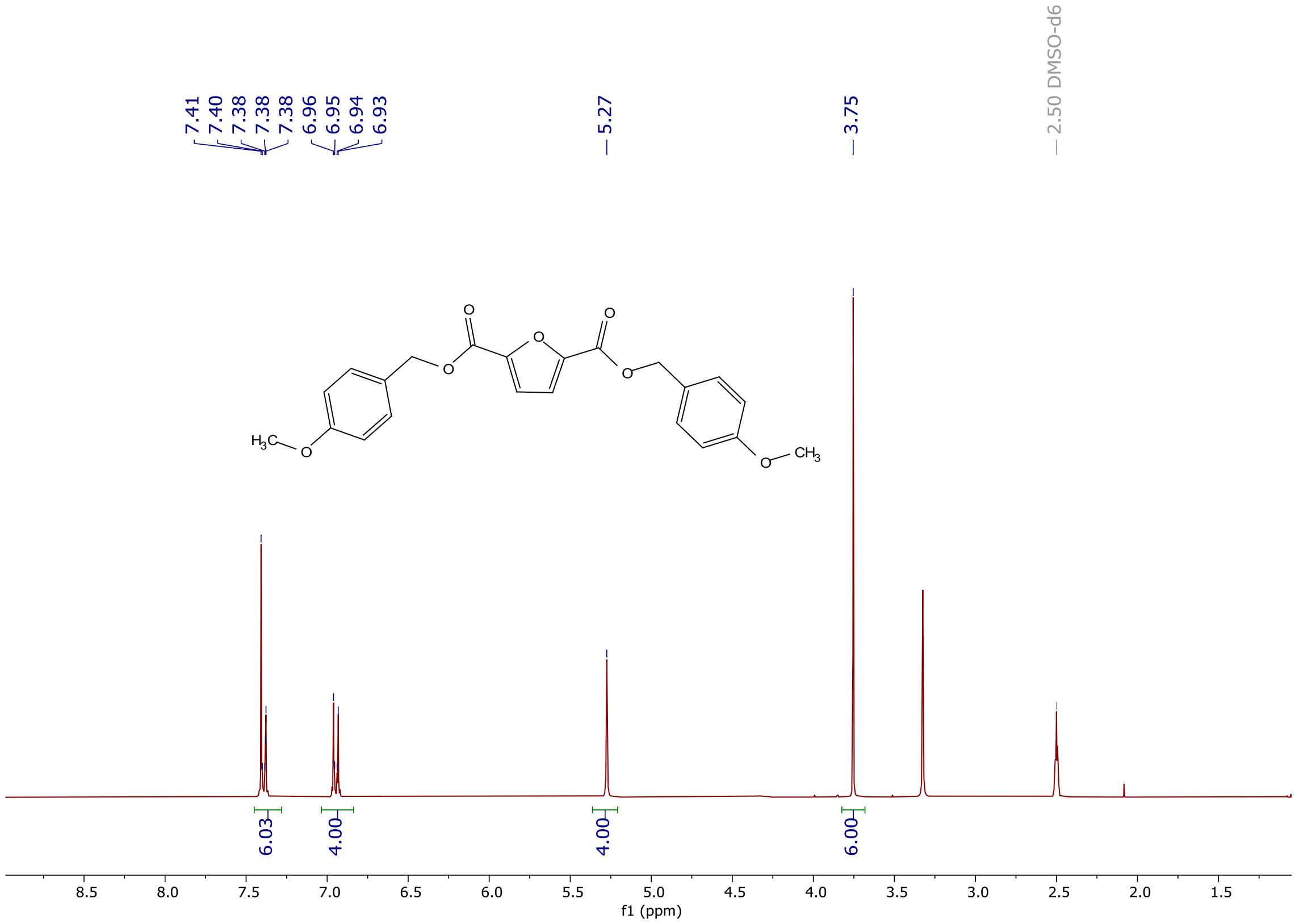

Figure S47. ^{1}H NMR spectrum ($CDCl_3$, 300 MHz, 297 K) of bis(4-methoxybenzyl) furan-2,5-dicarb (**4b**) from PBF.

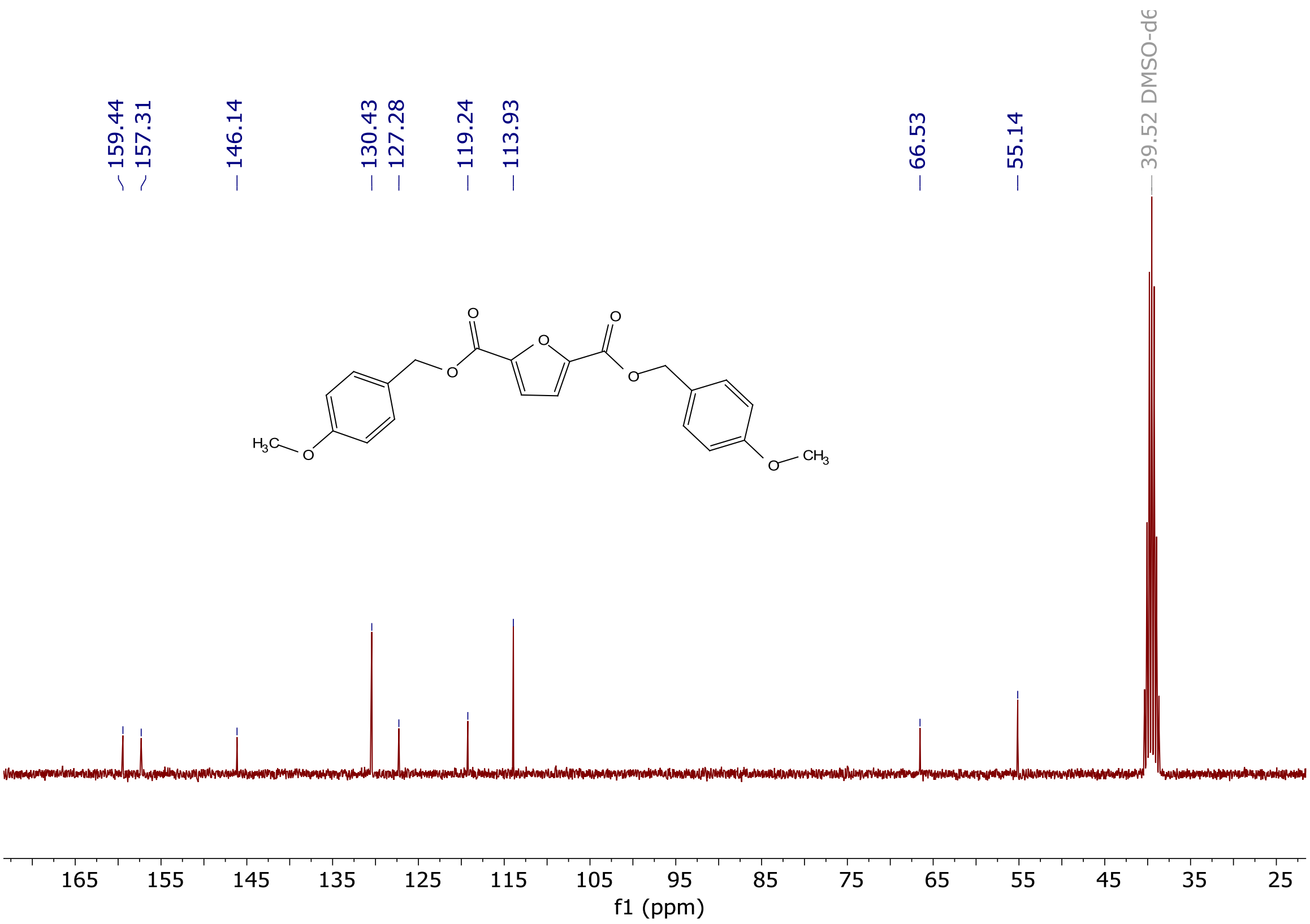

Figure S48. ^{13}C NMR spectrum ($CDCl_3$, 75 MHz, 297 K) of bis(4-methoxybenzyl) furan-2,5-dicarb (**4b**) from PBF.

Mechanochemical upcycling of PET

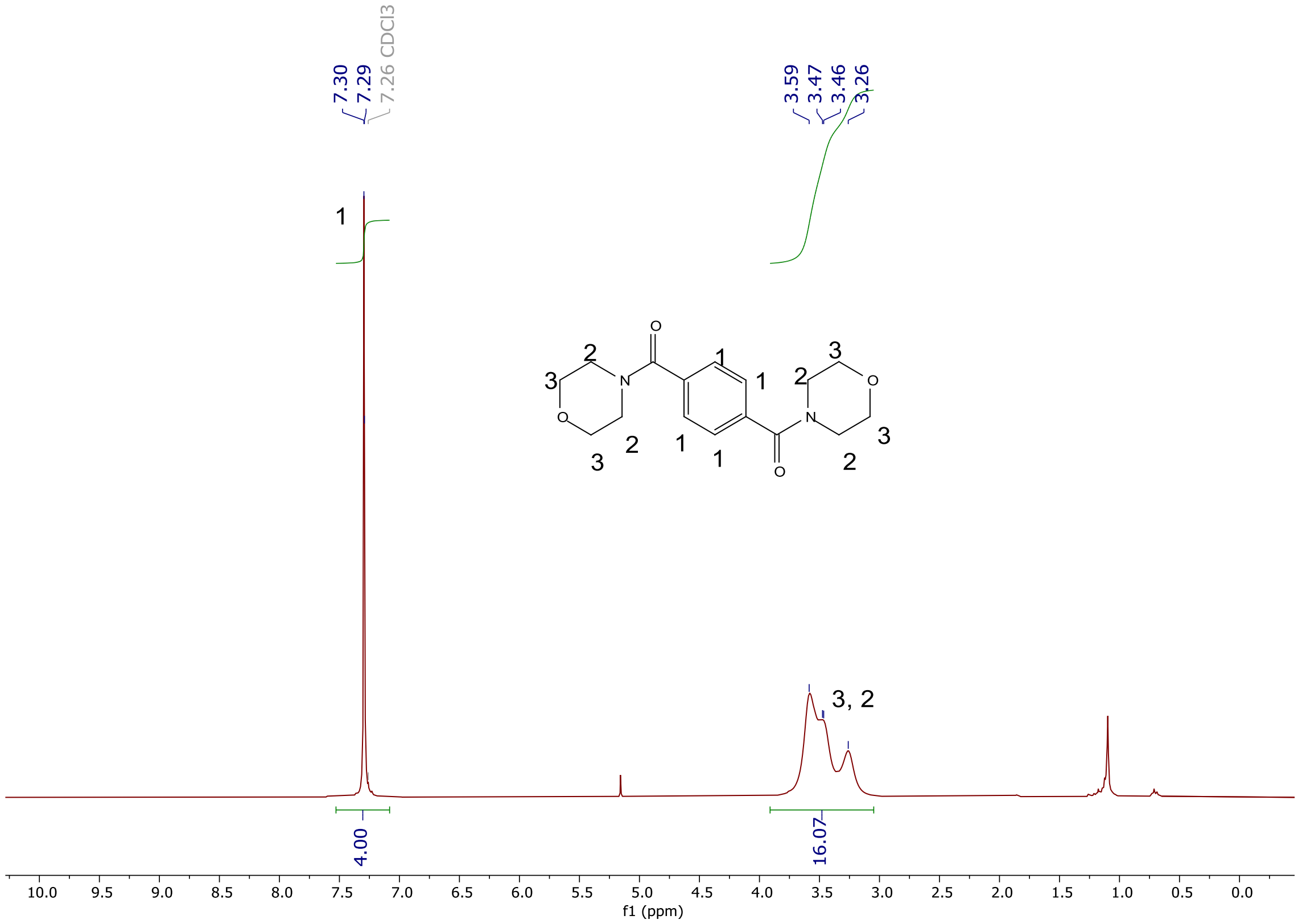

Figure S49. ^{1}H NMR spectrum ($CDCl_3$, 400 MHz, 297 K) of 1,4-phenylenebis(morpholinomethanone) (**5a**) from PET.

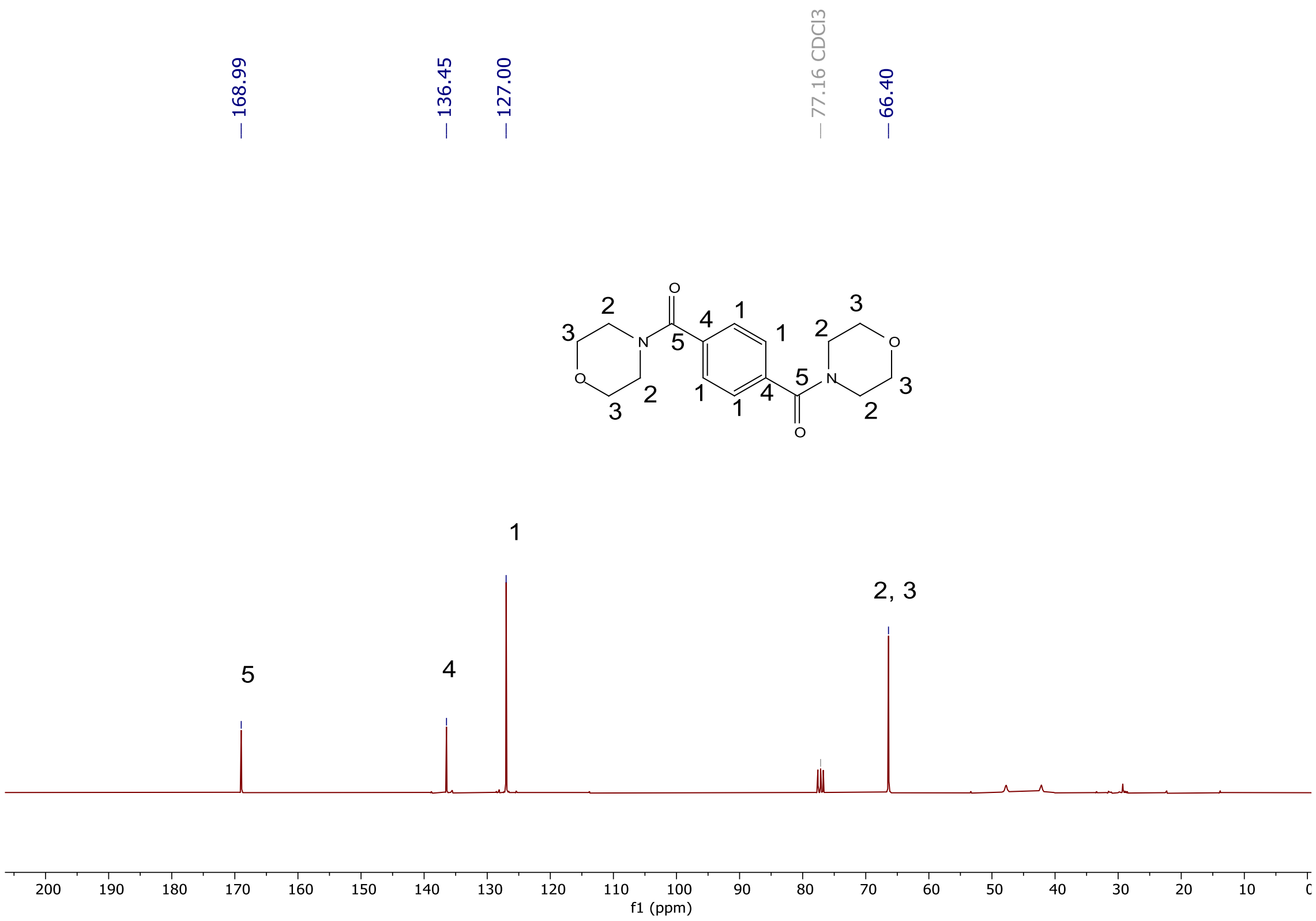

Figure S50. ^{13}C NMR spectrum ($CDCl_3$, 101 MHz, 297 K) of 1,4-phenylenebis(morpholinomethanone) (**5a**) from PET in $CDCl_3$.

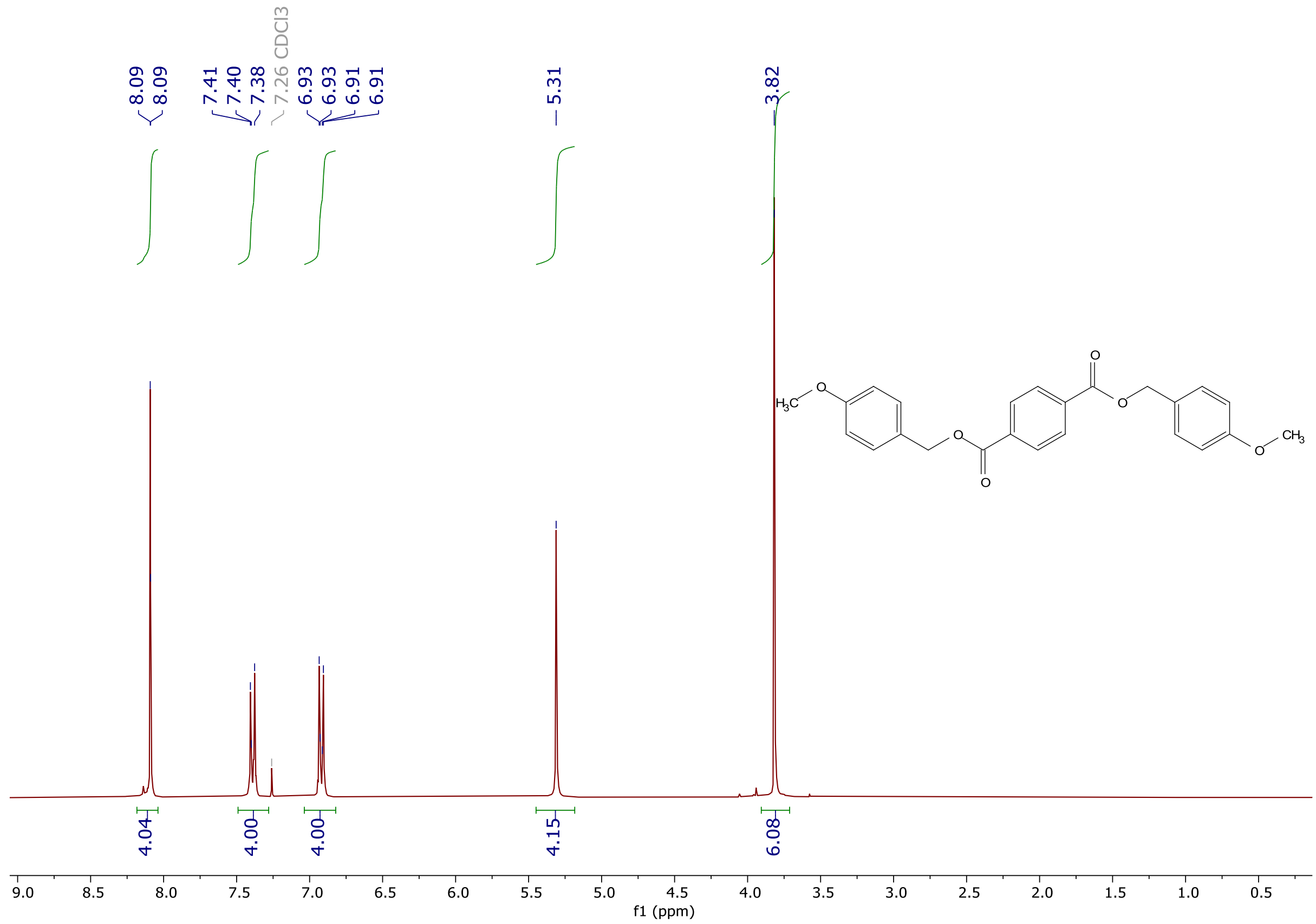

Figure S51. ^{1}H NMR spectrum ($CDCl_3$, 400 MHz, 297 K) of bis(4-methoxybenzyl) terephthalate (**5b**).

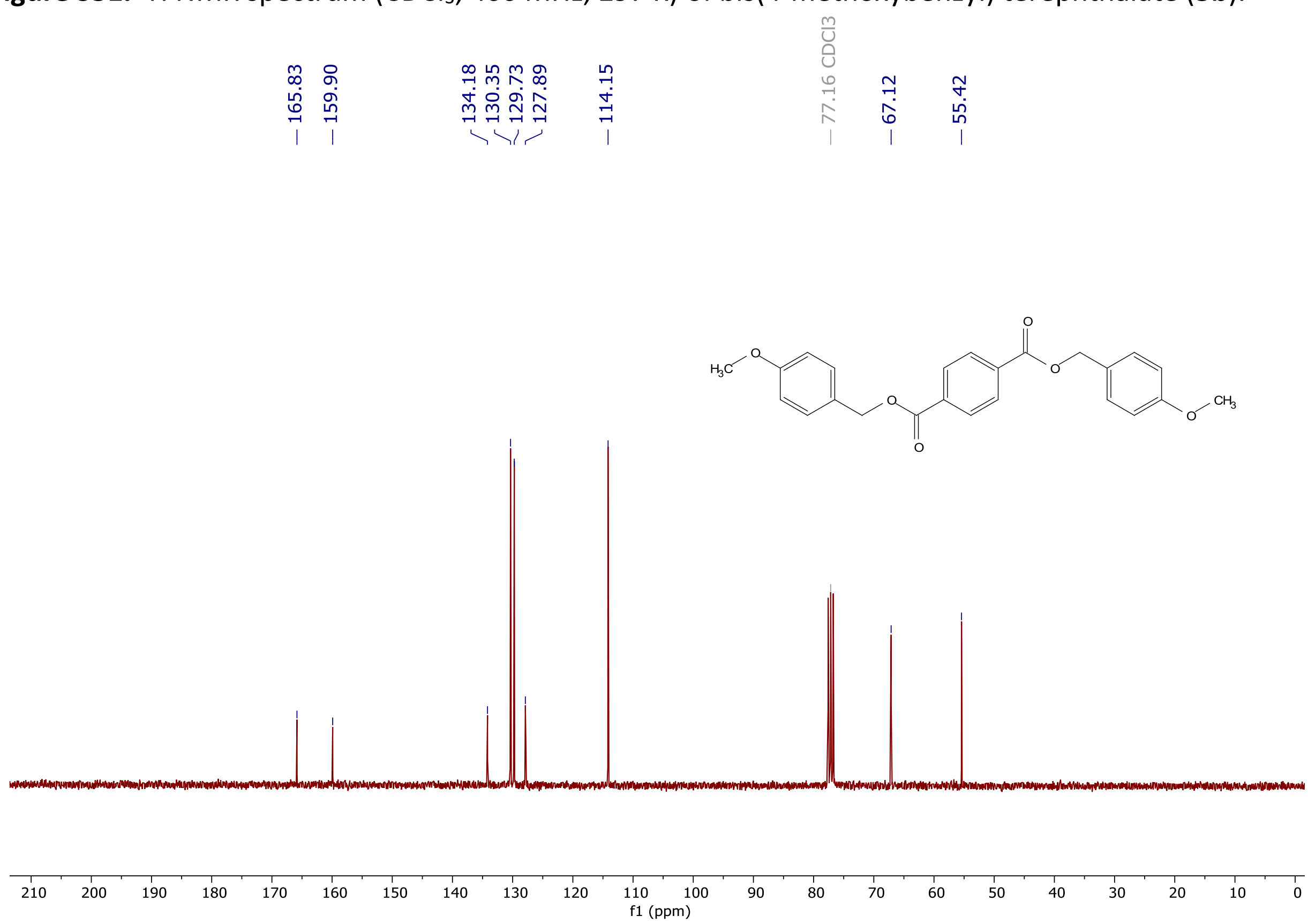

Figure S52. ^{13}C NMR spectrum ($CDCl_3$, 101 MHz, 297 K) of bis(4-methoxybenzyl) terephthalate (**5b**) in $CDCl_3$.

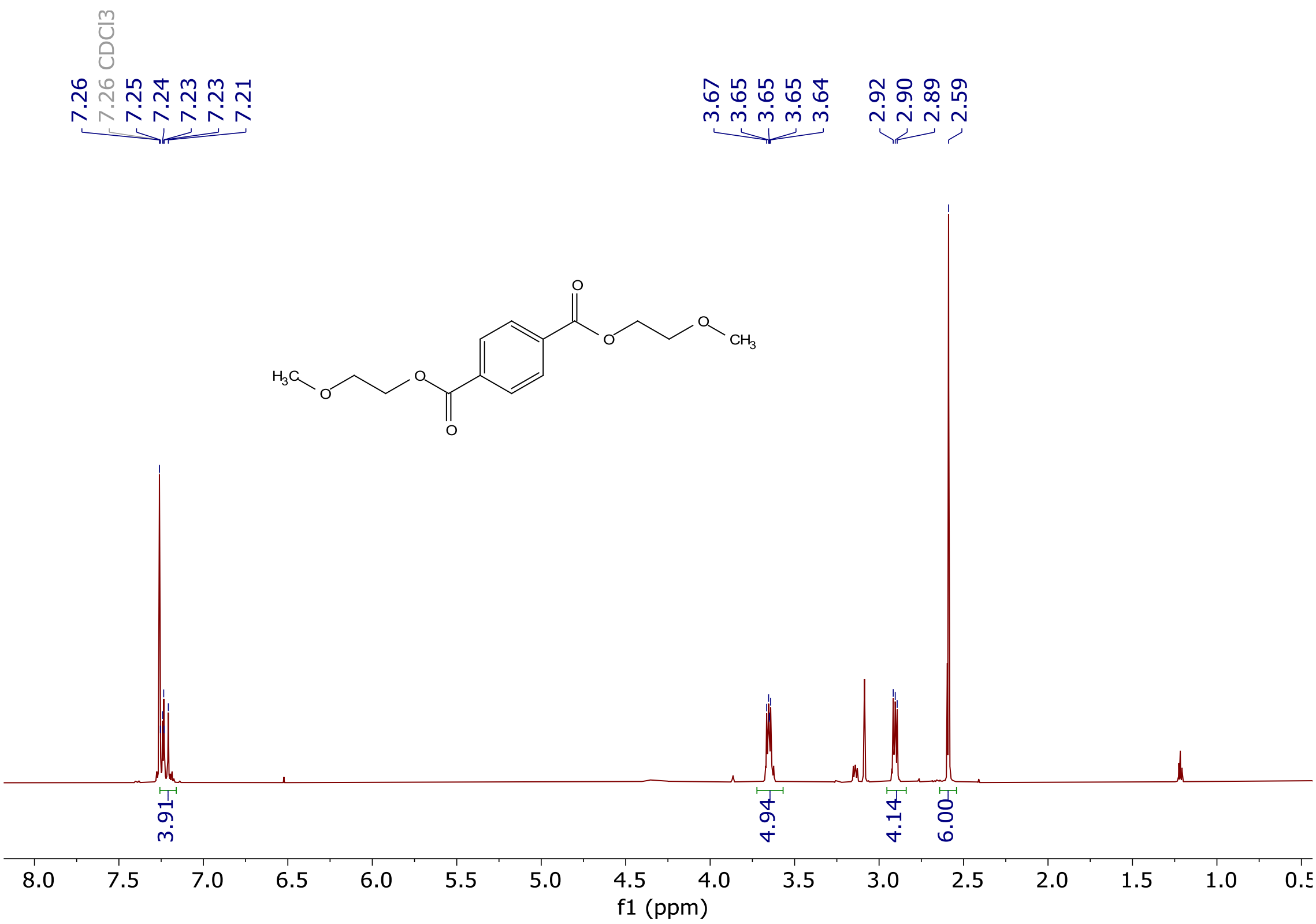

Figure S53. ^{1}H NMR spectrum ($CDCl_3$, 400 MHz, 297 K) of bis(2-methoxyethyl) terephthalate (**5c**).

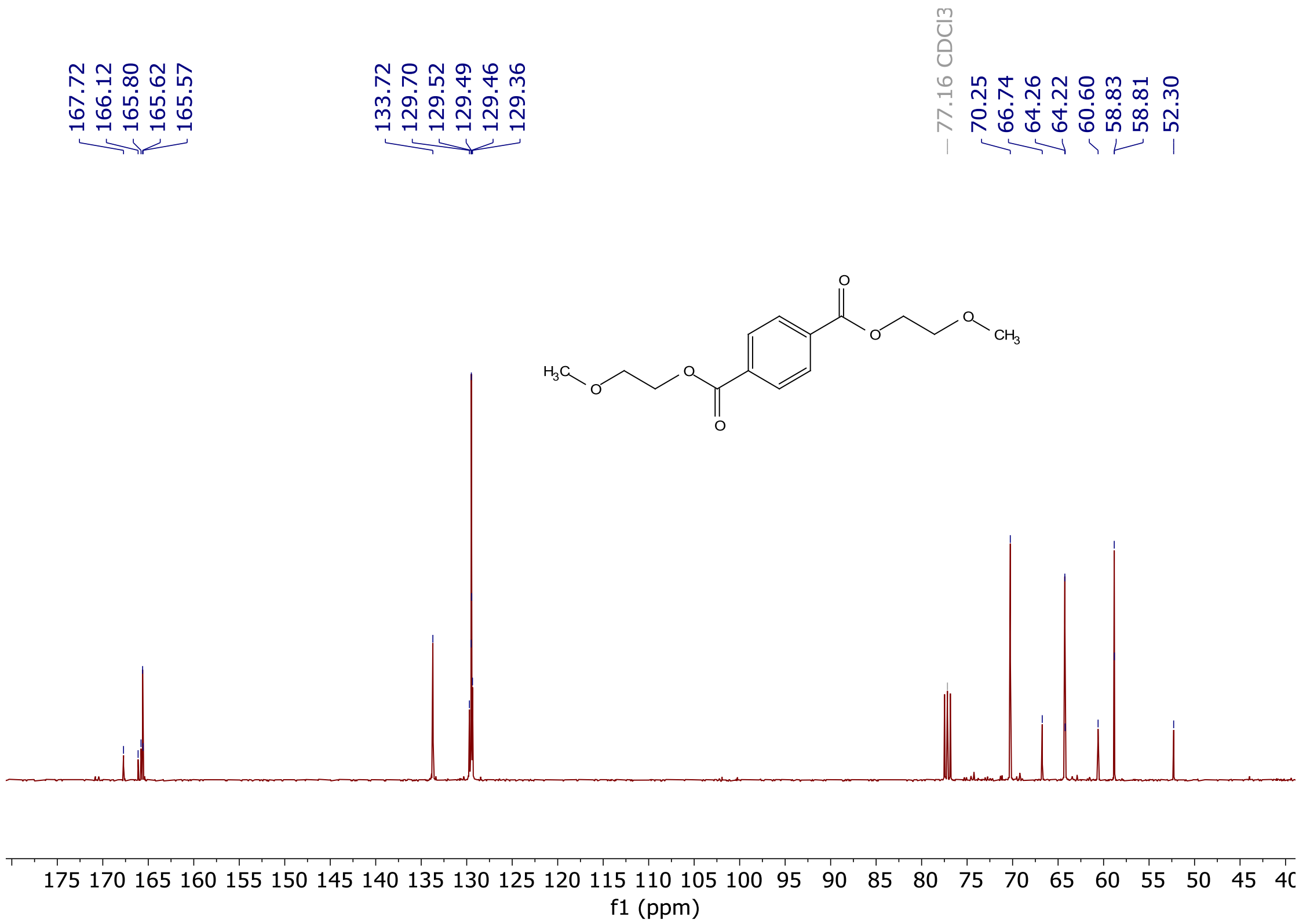

Figure S54. ^{13}C NMR spectrum ($CDCl_3$, 101 MHz, 297 K) of bis(2-methoxyethyl) terephthalate (**5c**) in CDCl3.

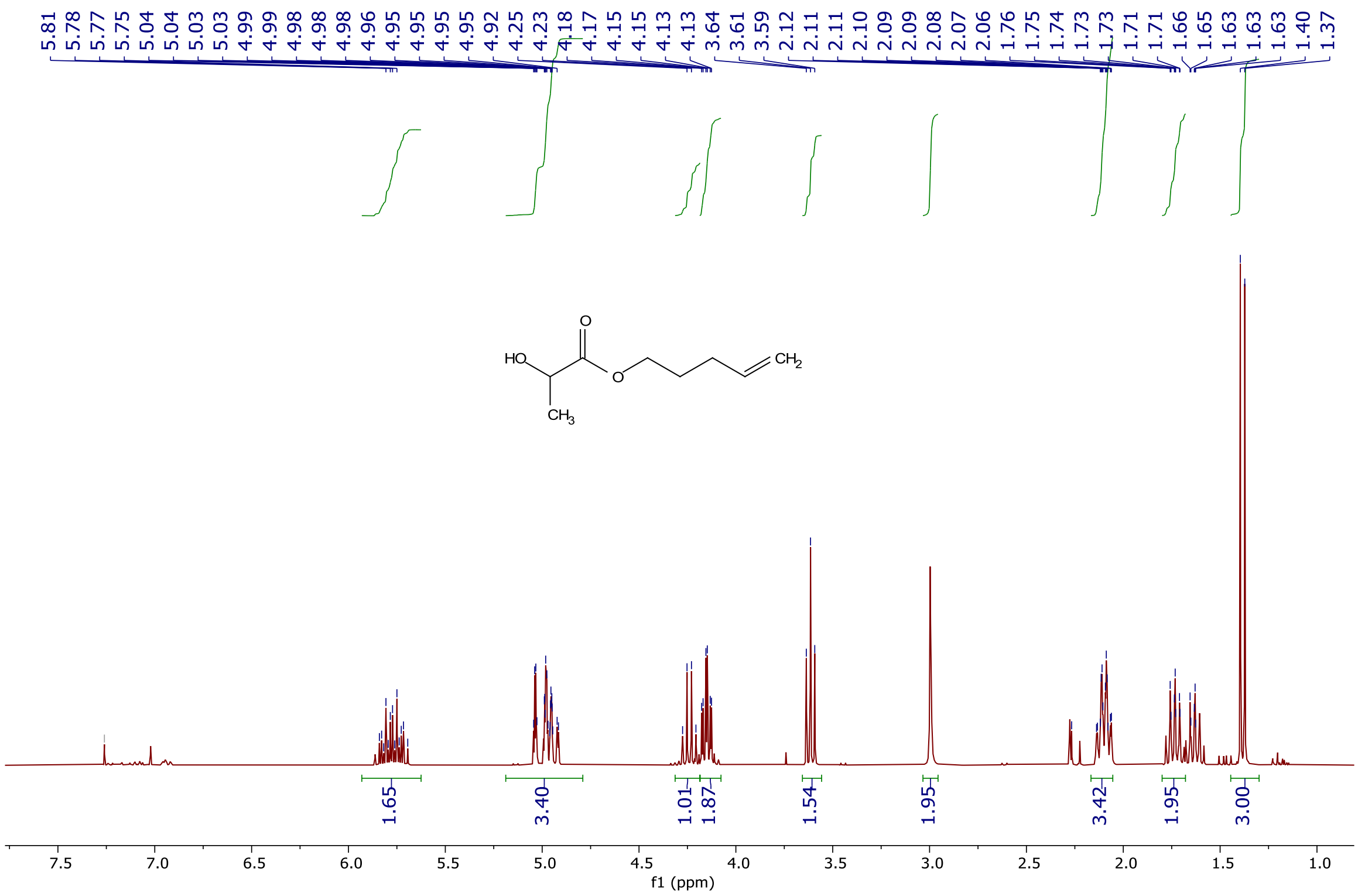

Figure S55. ^{1}H NMR spectrum ($CDCl_3$, 300 MHz, 297 K) of pent-4-en-1-yl 2-hydroxypropanoate (**6c**) from PLA. The NMR spectrum shows the presence of a single product with some remaining unreacted alcohol as this was used in excess. The absence of the quartet at 5.1 ppm indicates the full conversion of the PLA.

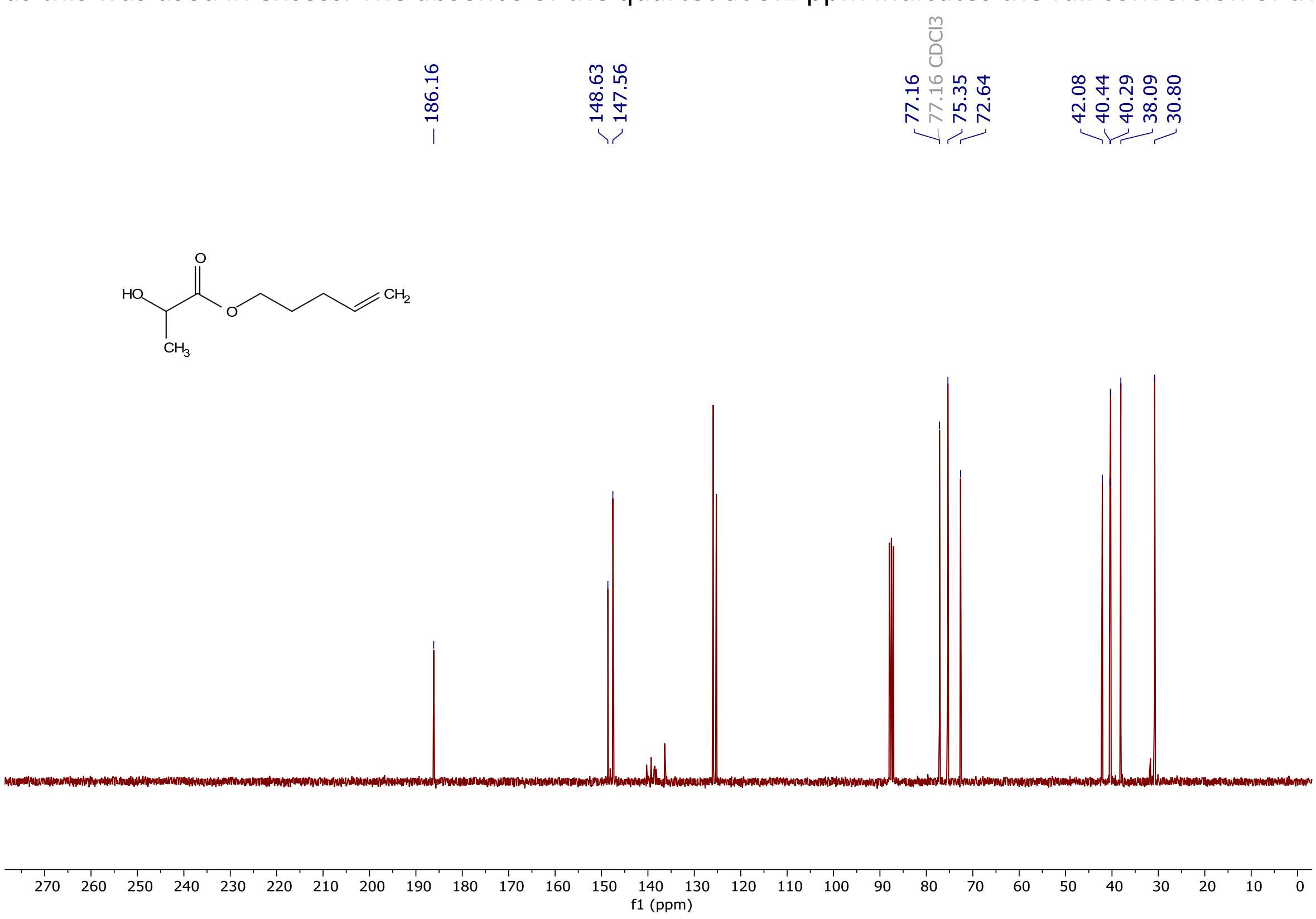

Figure S56. ^{13}C NMR spectrum ($CDCl_3$, 75 MHz, 297 K) of pent-4-en-1-yl 2-hydroxypropanoate(**6c**) from PLA. The presence of only one carbonyl resonance indicates the formation of a single product and the full conversion of PLA.

Crystallographic details

General information. X-ray quality crystals were selected in Fomblin YR-1800 perfluoroether (Alfa Aesar) at low temperature. Diffraction data were collected at 150(2) K on a Bruker Kappa APEX II Duo diffractometer using Mo-K_α radiation for compounds **3a** and **3c** or Cu-K_α radiation for compounds **3b**, **3i** and **5b**. The structures were solved by iterative (SHELXT) or direct methods (SHELXS-97[9]) and refined by full matrix least square techniques against F^2 (SHELXL-2014[10]). Semi-empirical absorption corrections were applied (SADABS/Bruker). The non-hydrogen atoms were refined anisotropically. The hydrogen atoms, except for the hydrogens at the nitrogen atoms in **3i** (determination and refinement from electron density), were placed in the theoretical positions and were refined by using the riding model. The structure of **3b** is of poor quality due to the small size of the crystals that resulted in weak reflexes. However, the collected data provide sufficient structural evidence and allow for the visualisation of the connectivity of this compound.

DIAMOND (Crystal Impact GbR) was used for structure representations.[11] Crystallographic data (excluding structure factors) for the structures reported in this paper have been deposited at the Cambridge Crystallographic Data Centre. Copies of the data can be obtained free of charge on application to CCDC, 12 Union Road, Cambridge, CB21EZ, UK (fax: int. code + (1223) 336-033; e-mail: deposit@ccdc.cam.ac.uk.

Table S3. Crystallographic data.

**Compound**	**(3b)** (structural proof)	**(3a)**	**(3c)**
Chem. Formula	$C_{20}H_{18}N_2O_3$· DMSO	$C_{14}H_{18}N_2O_5$	$C_{22}H_{20}O_7$
Formula weight [g/mol]	412.49	294.30	396.38
Colour	colourless	colourless	colourless
Crystal system	monoclinic	orthorhombic	monoclinic
Space group	$P2_1/c$	$Pna2_1$	$P2_1/n$
a [Å]	11.1660(8)	18.797(2)	15.2847(12)
b [Å]	23.5393(16)	5.8947(6)	6.5211(5)
c [Å]	15.6636(11)	11.8438(13)	19.0434(15)
α [°]	90	90	90
β [°]	90.268(5)	90	90.708(2)
γ [°]	90	90	90
V [Å^3]	4117.0(5)	1312.3(2)	1898.0(3)
Z	8	4	4
$\rho_{calcd.}$ [g/cm^3]	1.331	1.490	1.387
μ [mm^{-1}]	1.657	0.114	0.104
Measured reflections	25069	29037	17081
Independent reflections	6602	2874	4155
Reflections with $I > 2\sigma(I)$	5141	2607	2679
R_{int}	0.0752	0.0340	0.0407
F(000)	1744	624	832
$R_1(R[F^2>2\sigma(F^2)])$	0.1428	0.0285	0.0396
$wR_2(F^2)$	0.4008	0.0727	0.1069
GooF	1.182	1.027	1.014
No. of Parameters	528	190	264
Flack parameter			
CCDC #	2486066	2486065	2486064

Table S3, continued.

**Compound**	**(3i)**	**(5b)**
Chem. Formula	$C_{18}H_{14}N_2O_3$	$C_{24}H_{22}O_6$
Formula weight [g/mol]	306.31	406.41
Colour	colourless	orthorhombic
Crystal system	monoclinic	$P2_12_12_1$
Space group	$P2_1/c$	$P2_1/n$
a [Å]	9.5768(4)	6.0306(2)
b [Å]	17.0804(7)	8.1075(3)
c [Å]	9.4838(4)	40.1401(15)
α [°]	90	90
β [°]	93.361(2)	90
γ [°]	90	90
V [Å^3]	1548.65(11)	1962.57(12)
Z	4	4
$\rho_{calcd.}$ [g/cm^3]	1.314	1.375
μ [mm^{-1}]	0.745	0.814
Measured reflections	17205	28157
Independent reflections	2630	3431
Reflections with $I > 2\sigma(I)$	2243	3275
R_{int}	0.0341	0.0277
$F(000)$	640	856
$R_1(R[F^2>2\sigma(F^2)])$	0.0314	0.0245
w$R_2(F^2)$	0.0849	0.0656
GooF	1.027	1.022
No. of Parameters	216	273
Flack parameter		0.08(4)
CCDC #	2486063	2486066

References

1 D. Jain, F. Cramer, P. Shamraienko, H.-J. Drexler, B. Voit and T. Beweries, Highly efficient mechanochemical depolymerisation of bio-based polyethylene furanoate and polybutylene furanoate, *RSC Sustainability* **2025**, *3*, 3513–3519.

2 R. Androsch, C. Schick, A. M. Rhoades, "Application of Tammann's Two-Stage Crystal Nuclei Development Method for Analysis of the Thermal Stability of Homogeneous Crystal Nuclei of Poly(ethylene terephthalate)" *Macromolecules* **2015**, 48, 22, 8082–8089.

3 D. Rabaud, P. Dussart, G. Ducouret, P.- A. Albouy, J. Forté, B. Isare, L. Bouteiller, "Development of furan-2,5-dicarboxylic acid (FDCA)-based organogelators." *Soft Matter*, **2023**,*19*, 6958-6967.

4 W. Luo, K. Jiang, Y. Li, H. Jiang, B. Yin, "Direct Alkoxycarbonylation of Heteroarenes via Cu-Mediated Trichloromethylation and In Situ Alcoholysis." *Org. Lett.* **2020**, *22*, 2093–2098.

5 Y. Ogiwara, K. Nomura, "Chemical Upcycling of PET into a Morpholine Amide as a Versatile Synthetic Building Block." *ACS Organic & Inorganic Au* **2023**, *3*, 377–383.

6 V. Prakash Reddy, Golam Rasul, G. K. Surya Prakash, George A. Olah. "13C NMR/DFT/GIAO Studies of Phenylene Bis(1,3-dioxolanium) Dications and 2,4,6-Triphenylene Tris(1,3-dioxolanium) Trication." *J. Org. Chem.* **2003**, *68*, 3507–3510.

7 L. Yuan, Y. Hu, Z. Zhao, G. Li, A. Wang, Y. Cong, F. Wang, T. Zhang, N. Li, "Production of Copolyester Monomers from Plant-Based Acrylate and Acetaldehyde." *Angew. Chem. Int. Ed*. **2022**, *61*, e202113471.

8 A. S. Makarov, M. Rueping, "Scalable depolymerizing transesterification and amidation of (poly)lactic acid (PLA) enabled by resonant acoustic mixing (RAM)." *Green Chem.* **2025**, *27*, 716–721.

9 G. M. Sheldrick, "A short history of SHELX." *Acta Cryst., Sect. A: Found. Crystallogr.* **2008**, *64*, 112-122.

10 G. Sheldrick, "Crystal structure refinement with SHELXL." *Acta Crystallographica Section C* **2015**, *71*, 3–8.

11 H. Putz, K. Brandenburg, *Diamond-Crystal and Molecular Structure Visualization*, Crystal Impact-GbR, Kreuzherrenstr. 102, 53227, Bonn, Germany, **2018**.
